# Genomic scans for selective sweeps through haplotype homozygosity and allelic fixation in 14 indigenous sheep breeds from Middle East and South Asia

**DOI:** 10.1038/s41598-021-82625-2

**Published:** 2021-02-02

**Authors:** Sirous Eydivandi, Mahmoud Amiri Roudbar, Mohammad Osman Karimi, Goutam Sahana

**Affiliations:** 1 Department of Animal Science, Behbahan Branch, Islamic Azad University, Behbahan, Iran; 2 Center for Quantitative Genetics and Genomics, Faculty of Technical Sciences, Aarhus University, 8830 Tjele, Denmark; 3 Department of Animal Science, Safiabad-Dezful Agricultural and Natural Resources Research and Education Center, Agricultural Research, Education and Extension Organization (AREEO), Dezful, Iran; 4 Department of Animal Science, Faculty of Agriculture, Herat University, Herat, Afghanistan

**Keywords:** Comparative genomics, Genetics, Animal breeding

## Abstract

The performance and productivity of livestock have consistently improved by natural and artificial selection over the centuries. Both these selections are expected to leave patterns on the genome and lead to changes in allele frequencies, but natural selection has played the major role among indigenous populations. Detecting selective sweeps in livestock may assist in understanding the processes involved in domestication, genome evolution and discovery of genomic regions associated with economically important traits. We investigated population genetic diversity and selection signals in this study using SNP genotype data of 14 indigenous sheep breeds from Middle East and South Asia, including six breeds from Iran, namely Iranian Balochi, Afshari, Moghani, Qezel, Zel, and Lori-Bakhtiari, three breeds from Afghanistan, namely Afghan Balochi, Arabi, and Gadik, three breeds from India, namely Indian Garole, Changthangi, and Deccani, and two breeds from Bangladesh, namely Bangladeshi Garole and Bangladesh East. The SNP genotype data were generated by the Illumina OvineSNP50 Genotyping BeadChip array. To detect genetic diversity and population structure, we used principal component analysis (PCA), admixture, phylogenetic analyses, and Runs of homozygosity. We applied four complementary statistical tests, F_ST_ (fixation index), xp-EHH (cross-population extended haplotype homozygosity), Rsb (extended haplotype homozygosity between-populations), and FLK (the extension of the Lewontin and Krakauer) to detect selective sweeps. Our results not only confirm the previous studies but also provide a suite of novel candidate genes involved in different traits in sheep. On average, F_ST_, xp-EHH, Rsb, and FLK detected 128, 207, 222, and 252 genomic regions as candidates for selective sweeps, respectively. Furthermore, nine overlapping candidate genes were detected by these four tests, especially TNIK, DOCK1, USH2A, and TYW1B which associate with resistance to diseases and climate adaptation. Knowledge of candidate genomic regions in sheep populations may facilitate the identification and potential exploitation of the underlying genes in sheep breeding.

## Introduction

Genetic diversity in livestock is important for improving productivity and addressing future challenges, including food security and mitigating climate change^1^. Sheep were probably first domesticated in the Fertile Crescent region approximately 10,000 years ago^2^. Asia has about 40 percent of the world’s sheep breeds and diverse agro-ecological conditions have led to the development of more than 80 native sheep breeds in different geographical districts of Iran, Afghanistan, India, and Bangladesh^3,4^. Sheep play an important role in the livelihood of many rural and nomadic families in these countries^4^. The number of sheep average from 2010 to 2018 were in India 64 million, Iran 42.7 million, Afghanistan 13.6 million, and Bangladesh 2 million.

Study of population structure gives information on anthropogenic activities and historical processes that have influenced recent gene pools and the genetic relationships among breeds (Ju et al. 2019). Population structure among breeds can be studied using principal component analysis (PCA), admixture and phylogenetic analyses. A range of demographic forces and evolutionary trends affects linkage disequilibrium (LD) patterns on the genome^5^. The LD patterns provide good historical information on the population demography.

Natural and artificial selections leave patterns on the genome that result in differences in allele frequencies among populations^6^. If the selection pressure is high at the level of an individual locus, the frequency of the selected variant increases. In addition, selection will change the diversity pattern around the selected variant through genetic hitchhiking, known as a selective sweep^7^. As a result, different genetic variations and various haplotype structures are fixed over time within separated subpopulations, leading to a wide range of farm animal breeds and distinct genetic populations^8^. Selective sweeps detected in livestock breeds can add to new information about their population history.

Several methods have been developed to scan genome-wide selective sweeps^9^. Most of the methods are based on: (1) increases in derived allele frequency and decreases in genetic variation near a selective sweep (hitchhiking) within a population, (2) haplotype length and structure measured by extended haplotype homozygosity (EHH) or EHH-derived statistics, and (3) the differentiation of genetic populations measured by F_ST_ or related statistics^10^.

To capture any signal in the genome, depending on the number of populations, temporal context scale, and type of selection signatures more than one method is often needed^6^. Therefore, we implemented four complementary statistical tests, F_ST_, FLK (the extension of the Lewontin and Krakauer), xp-EHH (cross-population extended haplotype homozygosity), and Rsb (extended haplotype homozygosity between-populations). We studied selection signature in 14 indigenous sheep breeds from Iran, Afghanistan, India and Bangladesh, the four neighboring countries located in the Middle East and South Asia having more than 80 indigenous sheep breeds adapted to diverse ecological conditions. The selection signatures can illuminate selection patterns at the genome level of these indigenous sheep breeds, from adaptation to local environment and selection by breeders to improve production.

## Materials and methods

### Populations and genotypic data

We employed 50 K SNP genotype data on 453 individuals from 14 indigenous sheep breeds located in Iran, Afghanistan, India, and Bangladesh. Unpublished genotype data from three indigenous Iranian sheep breed, Iranian Balochi (IBL), Lori-Bakhtiari (LOR), Zel (ZEL) were used along with publicly available genotype data on another three Iranian sheep breeds, namely Afshari (AFS), Moghani (MOG), and Qezel (QEZ). We included data on three unpublished genotype data of Afghan sheep breeds, Arabi (ARB), Afghan Balochi (BLO), and Gadik (GDK). From South Asia, we included three Indian sheep breeds, Changthangi (CHA), Indian Garole (GAR), Deccani (IDC), and two Bengal sheep breeds, Bangladeshi Garole (BGA) and Bangladesh East (BGE)^11^. Information on these 14 breeds is summarized in Table 1.

**Table 1:** Breed names and the corresponding code used throughout the manuscript, the country of origin, sample size, and data source.

Breed	Acronym	Geographic origin	Category	Sample size	Data source	Dominant color	Tail status	Product	Climate adaptation
Afshari	AFS	Northwest of Iran	IR^a^	37	HapMap	Dark brown	Fat-tailed	Meat-wool	Cold and dry climate
Moghani	MOG	Northwest of Iran	IR	34	HapMap	White	Fat-tailed	Meat-wool	Cold and dry climate
Qezel	QEZ	Northwest of Iran	IR	35	HapMap	Red	Fat-tailed	Meat-wool	Cold and dry climate
Zel	ZEL	North of Iran	IR	44	Unpublished data	White	Thin-tailed	Meat-milk	Mild and forest
Lori-Bakhtiari	LOR	West of Iran	IR	46	Unpublished data	White	Fat-tailed	Meat	Mild and cold mountainous
Iranian Balochi	IBL	Southeast of Iran	AF^b^	87	Unpublished data	White	Fat-tailed	Wool-meat	Arid subtropical areas
Arabi	ARB	West of Afghanistan	AF	14	Unpublished data	Brown	Fat-tailed	Meat-milk	Arid areas
Afghan Balochi	BLO	Southwest of Afghanistan	AF	15	Unpublished data	White	Fat-tailed	Wool-meat	Arid subtropical areas
Gadik	GDK	North of Afghanistan	AF	14	Unpublished data	White	Fat-tailed	Wool-meat	Mild and cold mountainous
Bangladeshi Garole	BGA	West of Bangladesh	IN^c^	24	HapMap	Light brown	Thin-tailed	Meat	Hot and humid
Bangladesh East	BGE	East of Bangladesh	IN	24	HapMap	Light brown	Thin-tailed	Meat	Hot and humid
Indian Garole	GAR	Northeast of India	IN	26	HapMap	Light brown	Thin-tailed	Meat	Hot and humid
Changthangi	CHA	Northwest of India	IN	29	HapMap	White	Thin-tailed	Meat-wool	Mild and cold the mountainous
Deccani	IDC	South of India	IN	24	HapMap	Black	Thin-tailed	Meat	Semi-arid

^a^Contain Iranian sheep breeds exception Iranian Balouchi.

^b^Contain Afghan sheep breeds and Iranian Balouchi.

^c^Contain Indian and Bengal sheep breeds.

### Genotype quality control

OvineSNP50 BeadChip (Illumina, San Diego, CA, USA) was used to genotype animals. The SNP location information was taken from the Illumina Oar_v4 assembly, retrieved from SNPChiMp v.3^12^.

The genotype data from different breeds were merged using PLINK^13^. We excluded the SNPs located on sex chromosomes and those with unknown chromosomal position. The quality control was performed using PLINK^13^. SNPs that were genotyped in less than 90% of the animals, had a minor allele frequency (MAF) lower than 1%, or departed from Hardy–Weinberg proportions at a P-value < 10^–3^ were discarded. Furthermore, individuals with more than 10% missing genotypes were removed from the data set. After quality control, we used Beagle V5.0 software to impute sporadic missing genotypes^14^. The fcGENE v1.7 software was used to convert the PLINK formatted files to Beagle format and vice versa^15^.

### Genetic diversity and population structure

Individual genetic distances for the 14 sheep breeds were represented by a neighbor-joining tree and displayed using VCF-kit v0.1.6^16^ and FigTree.v1.4.4^17^.

We performed a PCA to investigate the population structure and to check whether samples for a breed came from a homogeneous population. PCA was done for the 14 sheep breeds using the smartpca program, which is part of EIGENSOFT 7.2.1^18^.

Linkage disequilibrium (mean of r^2^) among SNPs was estimated for the breeds using PopLDdecay v1.01 software, and a Perl script was applied to visualize the results^19^.

### Admixture analysis

For admixture analysis, quality filtered genotype data were pruned using PLINK based on LD. In a sliding window of 50 SNPs, LD pruning was carried out, moving the window in steps of 5 SNPs at a time, and removing all SNPs within each window exceeding the 1.7 variance inflation factor (*VIF*) threshold (–*indep* 50 5 1.7). VIF is known as 1/(1 − *r*^*2*^), with r^2^ being the correlation of the squared inter-variant allele count^20^.

We analyzed ancestry using ADMIXTURE v1.3.0 to infer breed origins and quantify the populations' admixture^21^. For a priori defined ancestry component (K), individual ancestry proportions were calculated with ADMIXTURE v1.3.0, which was an assumption of the number of ancestral populations^20^. Using 14-fold cross-validation for K values ranging from 2 to 14, admixture analysis was performed. To identify the most likely number of ancestral populations, the lowest 14-fold cross-validation error was applied. Finally, the admixture graphs were visualized using the R package BITE^22^.

### Runs of homozygosity

Runs of homozygosity (ROHs) were studied for all 14 breeds. Using the R package “detectRUNS”^23^, ROHs were calculated. The sliding window method was applied to calculate ROH segments^24^. Conditions used to detect segments of ROH were: sliding window size (windowSize = 15 SNPs), minimum number of homozygous SNPs in a run (minSNP) = 20, threshold of windows overlapping, homozygous (threshold) = 0.05, minimum number of SNP per kbps (minDensity) = 1/103, maximum distance between two SNPs (maxGap) = 106 bps, and the minimum length of a homozygous run (minLengthBps) = 250,000 bps. By default settings defined in the detectRUNS package, the ROHs detected were divided into five categories. (0 to $< { }$ 2 Mb, 2 to $<$ 4 Mb, 4 to $<$ 8 Mb, 8 to $<$ 16 Mb and $\ge$ 16 Mb). For each of the ROH length categories, the mean ROH sum per breed was determined by summing up all the ROHs per animal in that category and by averaging them per breed. The individual genomic inbreeding coefficient (FROH) was also determined as follows:

$$ F_{ROH = } \frac{{\Sigma L_{ROH} }}{Lgenome} $$

where $\Sigma L_{ROH}$ is the total length of all ROHs observed in an individual’s genome and $Lgenome$ is the sum of the length of the autosomes^23^.

### Selection sweep, gene annotation, and functional analysis

Neighbor-joining tree and PCA analysis divided the sheep populations in three distinct categories, IR (contains AFS, MOG, QEZ, ZEL, LOR breeds), IN (contains BGA, BGE, GAR, CHA, IDC breeds), and AF (contains IBL, ARB, BLO, GDK breeds) Table 1. Therefore, we compared pairwise these three categories for selective sweeps analysis.

### Selection sweep methods

We performed pairwise comparison for (a) IR vs. IN, (b) IR vs. AF, and (c) IN vs. AF to identify genomic regions under increasing differentiation using Fixation index (F_ST_), FLK (the extension of the Lewontin and Krakauer), xp-EHH (cross-population extended haplotype homozygosity), and Rsb (extended haplotype homozygosity between-populations).

The F_ST_ analysis is a widely used approach to identify genetic differentiations between populations compared to the within-population polymorphic frequency^25^. We performed the F_ST_ to identify genomic regions under increasing differentiation using VCFtools v0.1.15^26^. For each comparison, the mean of F_ST_ value was computed in all 39,348 SNPs. Z transformation of the mean of F_ST_ values (Z(F_ST_)) was performed using the “scale” command in R software.

The FLK test is an extension of the original Lewontin and Krakauer (LK) statistic^27^.

It calculates a population differentiation statistic, which includes a kinship matrix representing the relationship between populations^28^. This test accounts for population structure and differences in the effective population size by modeling the genetic divergence between populations as a result of drift and population division^29^.

For FLK analyses, p-values were computed as explained in the hapFLK software documentation^30^. For each comparison, the negative log p-value was calculated using the hapFLK R script^30^, and the candidate genomic regions under selection were plotted.

Extended haplotype homozygosity (EHH) detects selection signatures by comparing a high frequency and extended homozygosity based haplotype with other haplotypes at the selected locus^31^. Complete selective sweeps can be approached by using the cross-population EHH (xp-EHH) test, which compares each population regarding corresponding haplotypes to the other populations. The xp-EHH test compares the integrated EHH profiles between two populations at the same SNP^31^. The xp-EHH test has a high power to detect selection signatures in small sample sizes, and therefore grouping of genetically similar breeds may help in gaining power^32^^,^^31^.

Rsb test to identify selective sweeps is based on the same idea of estimation of EHH as xp-EHH test. However in contrast to xp-EHH test, it does not require phasing information^28^. Rsb compares the EHH patterns of the same allele between populations instead of comparing the EHH between alleles within one population, analogous to other statistics that are often focused on contrasting genetic variation between populations^33^.We used the xp-EHH and Rsb approaches^33,34^ to determine selected alleles with higher frequency than expected according to their haplotype length to obtain recent and generally segregating selective sweeps. The haplotypes were phased with Beagle^14^, and then xp-EHH and Rsb scores were calculated for each haplotype within a population. Haplotype frequencies were computed for 39,348 SNPs. For each locus, the xp-EHH and Rsb score were calculated using the rehh package^35^ in R and the candidate genomic regions under selection were obtained.

For each test, the genes that were considered as candidates were found within the intervals spanning the candidate genome regions and also overlapping candidate genes among the tests were captured using the Ovis Oar_v4 reference genome assembly in the Ensembl^36^. The candidate genes visualized using Venpainter tool (Lin et al. 2016).

Absolute correlation among four methods used to detect selection sweeps on: (a) IR vs. IN, (b) IR vs. AF, and (c) IN vs. AF sheep breeds were determined using R codes.

The biological enrichment and functional annotation of the genes under selective pressure were defined using Gene Ontology Consortium (http://geneontology.org).

## Results

### Populations and genotype data

After quality control and imputation of missing genotypes from 463 individuals genotype data for 39,531 SNPs from 14 sheep breeds Table 1, 453 individuals and 39,348 SNPs remained for analysis. In details 10 individuals removed due to missing genotype data (–mind), 180 SNPs removed due to missing genotype data (–geno), and 3 SNPs were removed due to minor allele frequency (–maf).

### Population genetic structure and linkage disequilibrium

**Figure 1:**
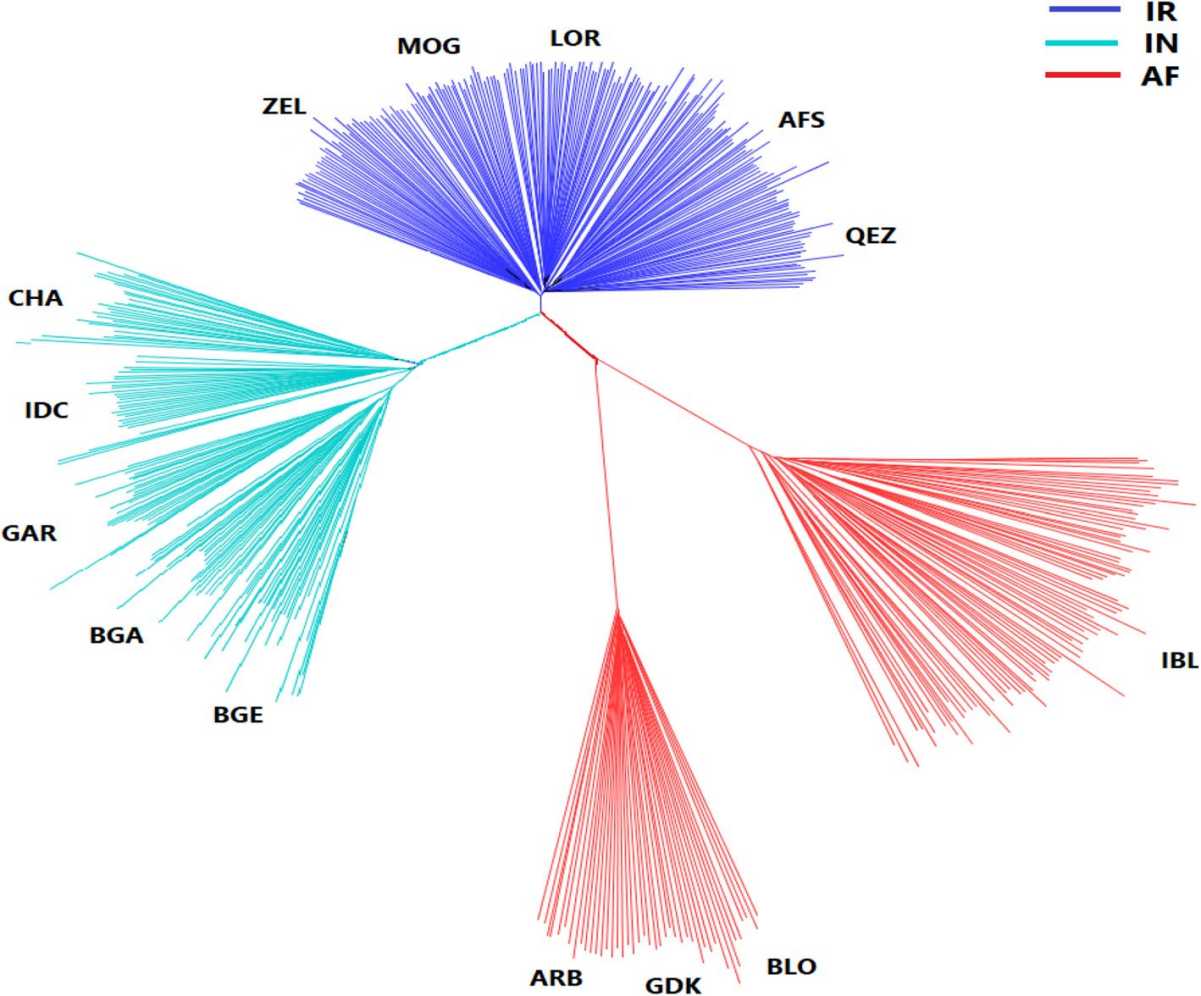
Neighbor-joining phylogenetic tree for 14 sheep breeds based on autosomal SNPs. For breed abbreviations, see Table 1.

The Neighbor-joining phylogenetic tree analysis divided the 14 breeds into three main branches, IR, IN, and AF. The IR group included AFS, MOG, QEZ, ZEL, and LOR, in a main branch Fig. 1, blue color), which illustrated close relationships in the blue branch. These five breeds are from mountainous and forest areas with cold and temperate climates of Iran. The AF group has two distinct sub-branches, one for the three Afghan breeds (ARB, BLO, GDK), and the other own for the Iranian IBL breed Fig. 1, red color). The IBL sheep is from a hot dry climate in the south-eastern deserts of Iran, bordering Afghanistan and Pakistan and therefore IBL is geographically closer to Afghan breeds than the other Iranian breeds in this study. The ARB, BLO, and GDK breeds formed a dense sub-branch that indicates their close genetic relationship. The IN branch included BGA, BGE, GAR, IDC, and CHA Fig. 1, green color). In this branch, two Bengal breeds (BGA and BGE) and GAR formed a distinct cluster, and two other Indian sheep breeds were placed in two separate clusters. The GAR and BGA which are both named Garole breed live in West Bengal state of India and Bangladesh, respectively however, some breeding isolation between them occurred. Therefore, a close genetic relationship between these two breeds is expected.

The LD patterns among the IR and IN groups indicated that the mean of correlation coefficient values (r^2^) in both groups dropped rapidly at approximately 10 Kb while the AF group showed a slower drop and its r^2^ values at 50 Kb was higher than the other groups (Supplementary Figure S3). The average r^2^ at 250 Kb for the IR, IN and AF breeds were 0.0351, 0.0230 and 0.0693, respectively. There was a big difference in r^2^ values more than 100 Kb between (IR and IN) and AF.

**Figure 2:**
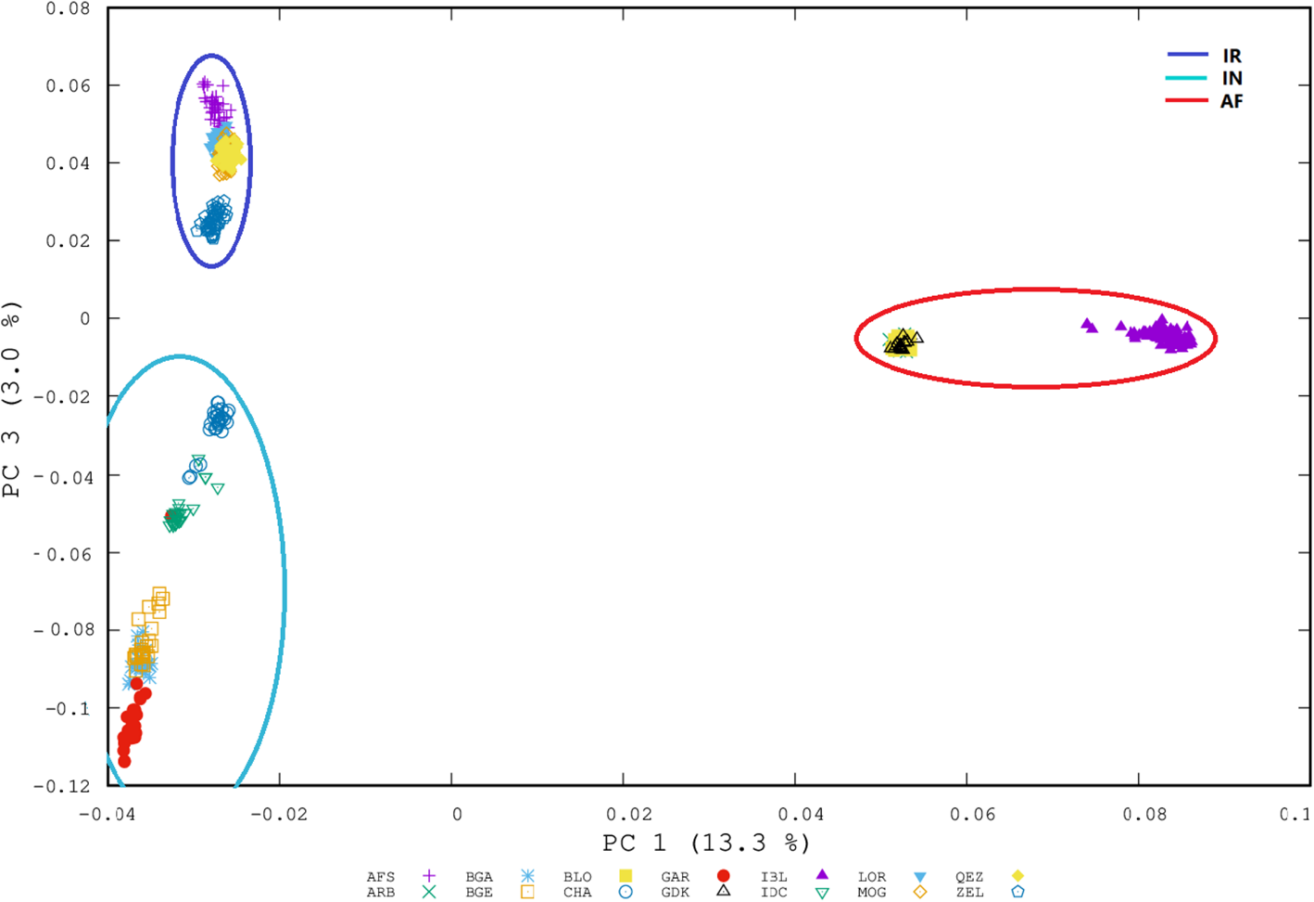
Principal components analysis (PC 1 and PC 3) of among 14 sheep breeds based on autosomal SNP markers. For breed abbreviations, see Table 1.

PCA results Fig. 2 also indicated close relationships within the IR, IN, and AF groups and supported separation into the three broad geographic groups that were identified by the neighbor-joining tree Fig. 1. Although the breeds clustered according to geographic origin, a gradient based on the geographic distance was less pronounced Fig. 2. In addition, the first principal component (PC1), explaining 13.3% of the total genetic variation among breeds, clearly separated the IR and IN breeds from the AF breeds, thus forming two clusters. Along with the PC1 projection spectra, both IBL and Afghan breeds formed the AF group but a large genetic variation are shown between them. Among the AF breeds, IBL is clearly distant from the other breeds and supported the phylogenetic results. The subclusters of MOG, GEZ, and AFS breeds overlapped, indicating a close relationship and possible admixture of these breeds from the same region in north-western Iran. The LOR breed clearly distant from the other IR breeds which show geographic distance between the LOR from the west and south-western of Iran and the other IR breeds from the north and north- western of Iran. The patterns of genetic variation observed for the AFS, MOG, and GEZ breeds suggested a recent admixture between these three Iranian breeds.

PC2, explaining 6.8% of the total genetic variation, separated the Afghan breeds from the other breeds, but it did not clearly show geographic distance between the IR and IN breeds (Supplementary Figure S1). PC3, explaining 3% of the total genetic variation, separated the IR from the IN and also showed close genetic relationship among two Bengal breeds and GAR, while genetic distances among IDC, CHA, and the other IN breeds Fig. 2. For a more clear assessment, we did PCA between the IR and IN breeds which were separated by PC1 (Supplementary Figure S2).

**Figure 3:**
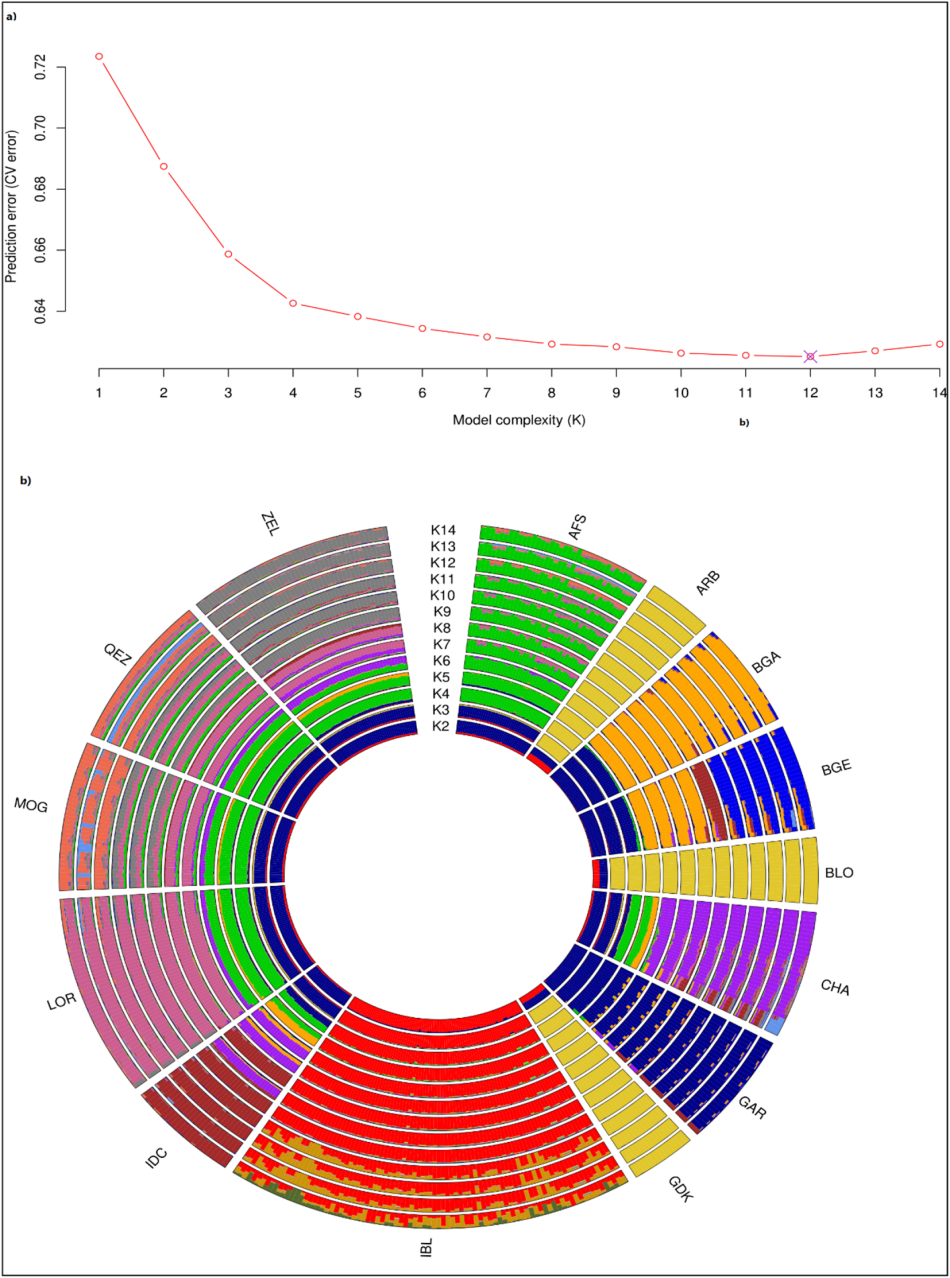
Prediction error (**a**) and circle admixture from K2 to K14 (**b**) plotted, respectively. For breed abbreviations, see Table 1.

The occurrence and extent of breed admixture were examined by estimating individual ancestry proportions from quality filtered and LD pruned genotype data. During pruning the dataset 13,257 of 39,348 SNPs removed and 26,091 SNPs were remained. Admixture analyses were carried out with up to 14 ancestral components (K) Fig. 3. Cross-validation (CV) errors were calculated to identify the most likely number of ancestral populations. The lowest CV error was detected for *K* = 12 Fig. 3a. Although at *K* = 10, CV errors had stagnated after a decline, ancestry components up to *K* = 10 separate breeds, and so it was recognized as the optimal value of *K* Fig. 3b.The results of Admixture were in general agreement as PCA. Although the AF breeds, especially the IBL from the IR and IN breeds, were separated at the first ancestry components (*K* = 2) and also at *K* = 4, the IR breeds were separated from the IN breeds but a substantial IR ancestry is observed in the CHA breed at K = 4. In addition, based on geographic origin the breeds were divided as follows: *K* = 2: the AF breeds,*K* = 4: the IN breeds,*K* = 7: the IR breeds. At *K* = 10. The breed-specific ancestry components were clearly defined by all breeds except MOG and QEZ and the Afghan breeds (ARB, BLO, and GDK). However, our finding showed that increasing the number of *K* above 10 did not yield a consistent MOG and QEZ separation. Therefore, four ancestral components distinguished the five IR breeds where AFS, ZEL, and LOR were unambiguously recognized. Similar to PCA findings, the fourth component was shared between MOG and QEZ, confirming a close genetic relationship. There were no differences among the ARB, BLO, and GDK Afghan breeds from *K* = 2 to *K* = 14 which indicated close genetic relation sheep of them, confirming results from PCA and the neighbor-joining tree. The Bengal breeds (BGA and BGE) separated from *K* = 9, but despite expectation, BGA and GAR with the common name and root separated from *K* = 5. In general, compared with the other IN breeds, closer genetic relationships were seen between BGA, BGE, and GAR confirming PCA and the neighbor-joining tree analyses.

### ROHs

A total of 34,587 ROH segments were identified in the 14 studied breeds. The results are summarized in Supplementary Table S5. The number of ROH segments was highest in IBL (5,407) and GAR (4,605) breeds, and lowest in ARB (1,000) and GDK (1,018). The majority of ROH segments were shorter than 8 Mb (32,260), followed by intermediate ROH segments ranging from 8 to 16 Mb (1,520) and large ROH segments exceeding 16 Mb (807). Based on ROH, levels of F_ROH_ for each breed were estimated separately (Supplementary Figure S4). Genomic inbreeding ranged from 0.008 (in the AFS breed) to 0.5 (in the GAR breed).

### Selective sweeps detection

**Figure 4:**
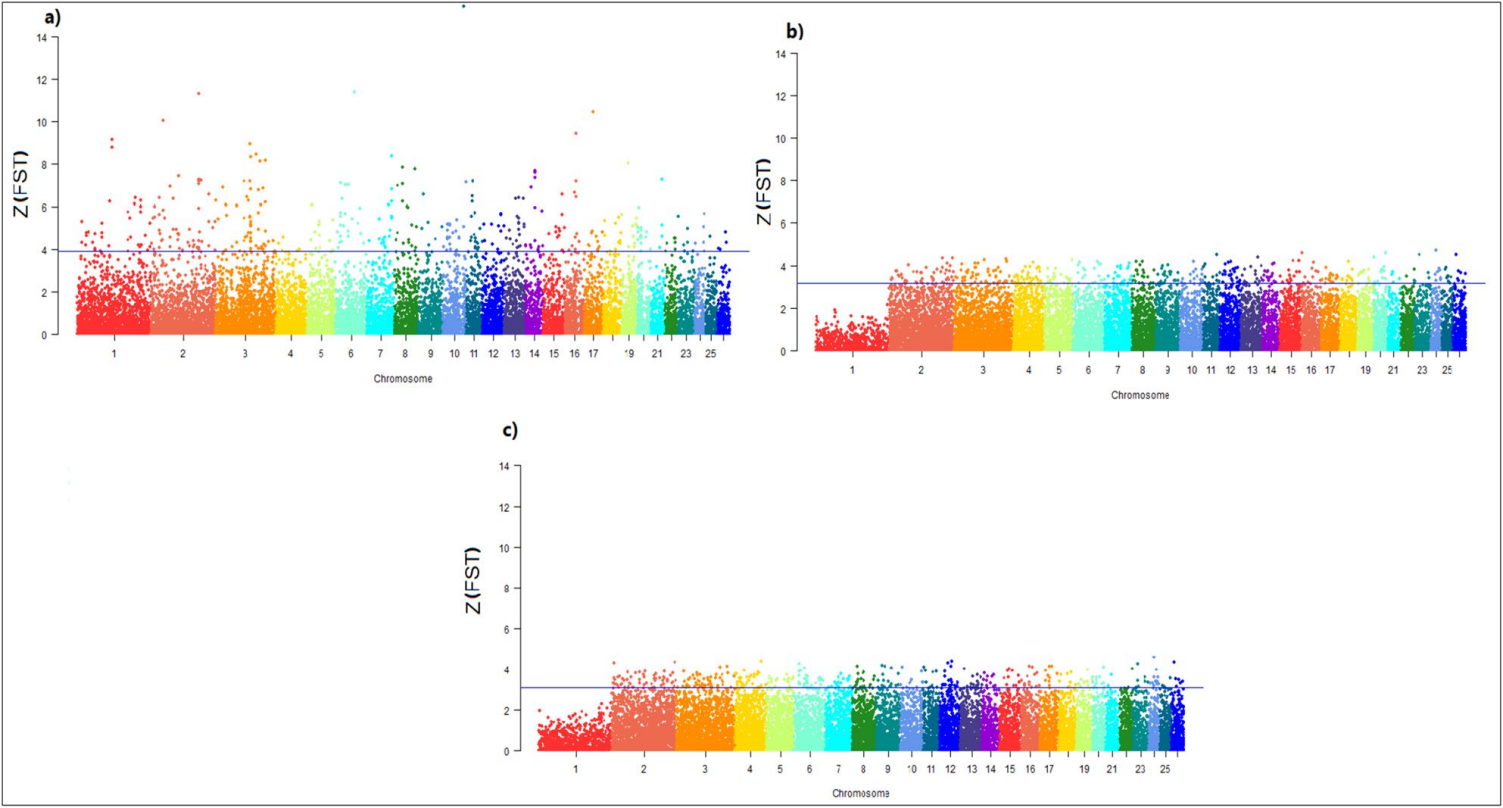
The distribution of absolute Z(F_ST_) values on 26 sheep autosomes: (**a**) IR vs. IN breeds (the horizontal blue line, Z(F_ST_) ≥ 3.93), (**b**) IR vs. AF breeds (the horizontal blue line, Z(F_ST_) ≥ 3.18), (**c**) IN vs. AF breeds (the horizontal blue line, Z(FST) ≥ 3.08). The data points above the horizontal line (blue line) are top 1% Z(FST) values. *FST* fixation index. For breed abbreviations, see Table 1.

Selective sweeps detection was performed using F_ST_^37^, FLK^27^, Rsb^33^, and xp-EHH^34^. Based on the PCA and the neighbor-joining tree results, these four different tests were conducted for selective sweeps detection on the three pairwise comparisons: (a) IR and IN breeds, (b) IR and AF breeds, (c) IN and AF breeds. The Z-transformation of F_ST_, Z(F_ST_), values of 39,348 SNPs were estimated Fig. 4. For these three pairwise comparison, the maximum of Z(F_ST_) values were 14.524 on chromosome 11 (IR vs. IN breeds), 4.744 on chromosome 24 (IR vs. AF breeds) and 4.556 located on chromosomes 24 (IN vs. AF breeds) Fig. 4. Based on the Z(F_ST_), a total of 131 genes as top 1% candidates for selective sweeps were detected in (a) IR and IN breeds, 131 genes in (b) IR and AF breeds, and 121 genes in (c) IN and AF breeds (Supplementary Table S1). Among these candidate genes, several of them are known for association with economic traits, for example, SLC27A6, ANXA13, ADCY2, HDAC9, TTC8, and WDR70 association with milk traits. HERC2, FTO, TP73, GRM3, KCNIP4, GRM7, and UBR2 are related to body weight and growth traits. TMEM132B, TMEM232, and SLC8A3 affect fertility traits. ADAMTS6, ADAMTS20, GALNT6, ATP2C1, TMPRSS3, PCDH15, MAGI2, TRPC4, DOCK1, DOCK4, DOCK10, MAPK10, ADAM7, PPA2, CHD3, ITGA4, NBEAL1, NFATC1, and ZNF609 involve in the immune system and environment adaptation.

**Figure 5:**
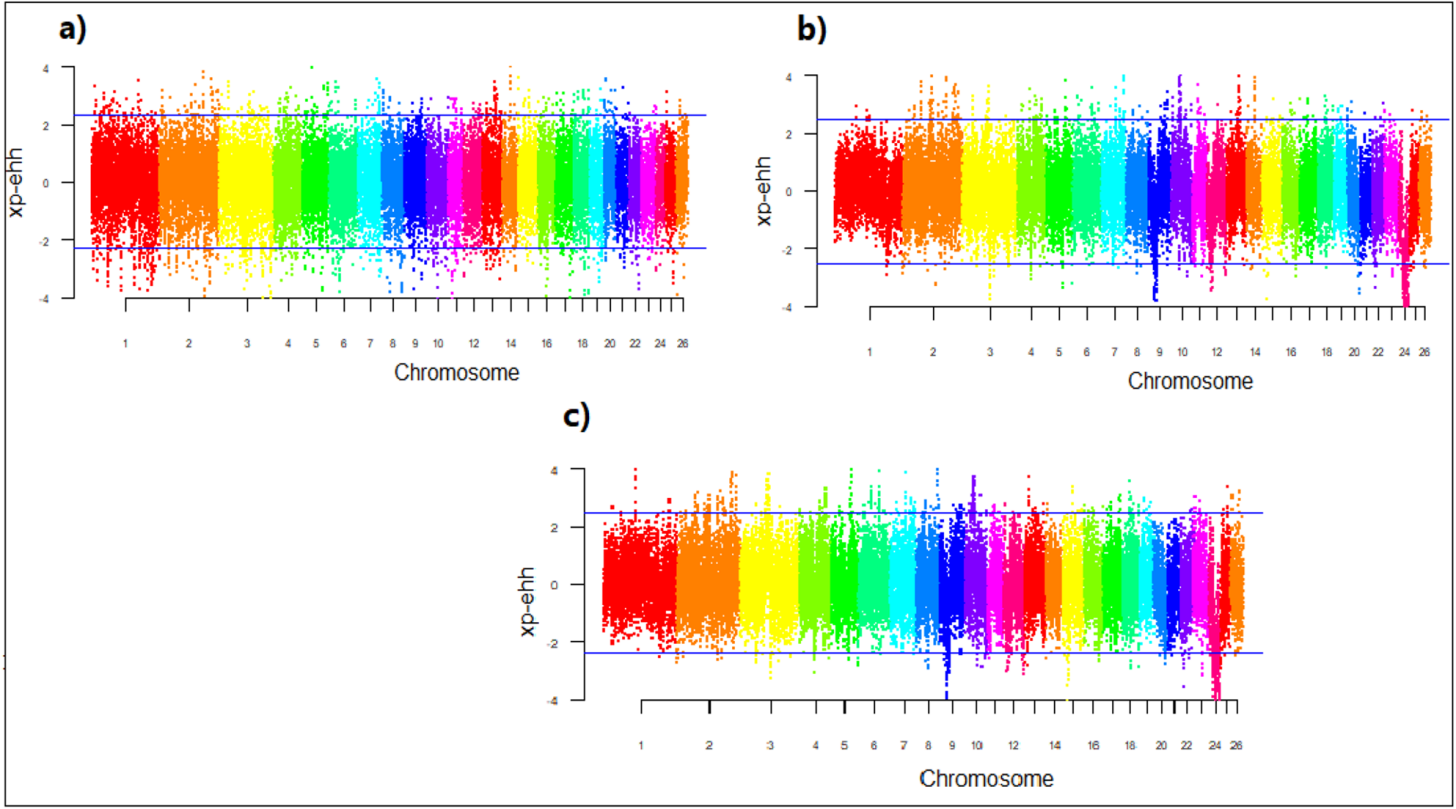
Genomic distribution of standardized cross-population extended haplotype homozygosity (xp-EHH) scores on 26 sheep autosomes pairwise: (**a**) IR and IN breeds, (**b**) IR and AF breeds, (**c**) IN and AF breeds. For breed abbreviations, see Table 1.

The xp-EHH scores were calculated for haplotype frequencies Fig. 5. The top 1% of xp-EHH, considered as selective sweeps, identified 164 genes for (a) IR and IN breeds,236 genes for (b) IR and AF breeds; and 221 genes for (c) IR and AF breeds (Supplementary Table S2). Many candidate genes found by the xp-EHH method are related to economic traits, such as, OXT, HSPB1, TBX6, GNA12, BMP7, MYH10, TRHDE, IL27, IL4R, and IL21R involved in heat stress; ATP2A1, ATP2B1, LRP12, CD19, MAPK3, PLCE1, VPS16, PTPRA, ADAM2, MYO18A, PCDH17, BBS9, NFATC2IP, RNF26, RNF139, ZNF572, ZNF655, and ZNF789 associated with immune system and environment adaptation; and MEF2C, TRHDE, FAM222B, FAM177A1, and SSC4D influenced body weight and growth traits.

**Figure 6:**
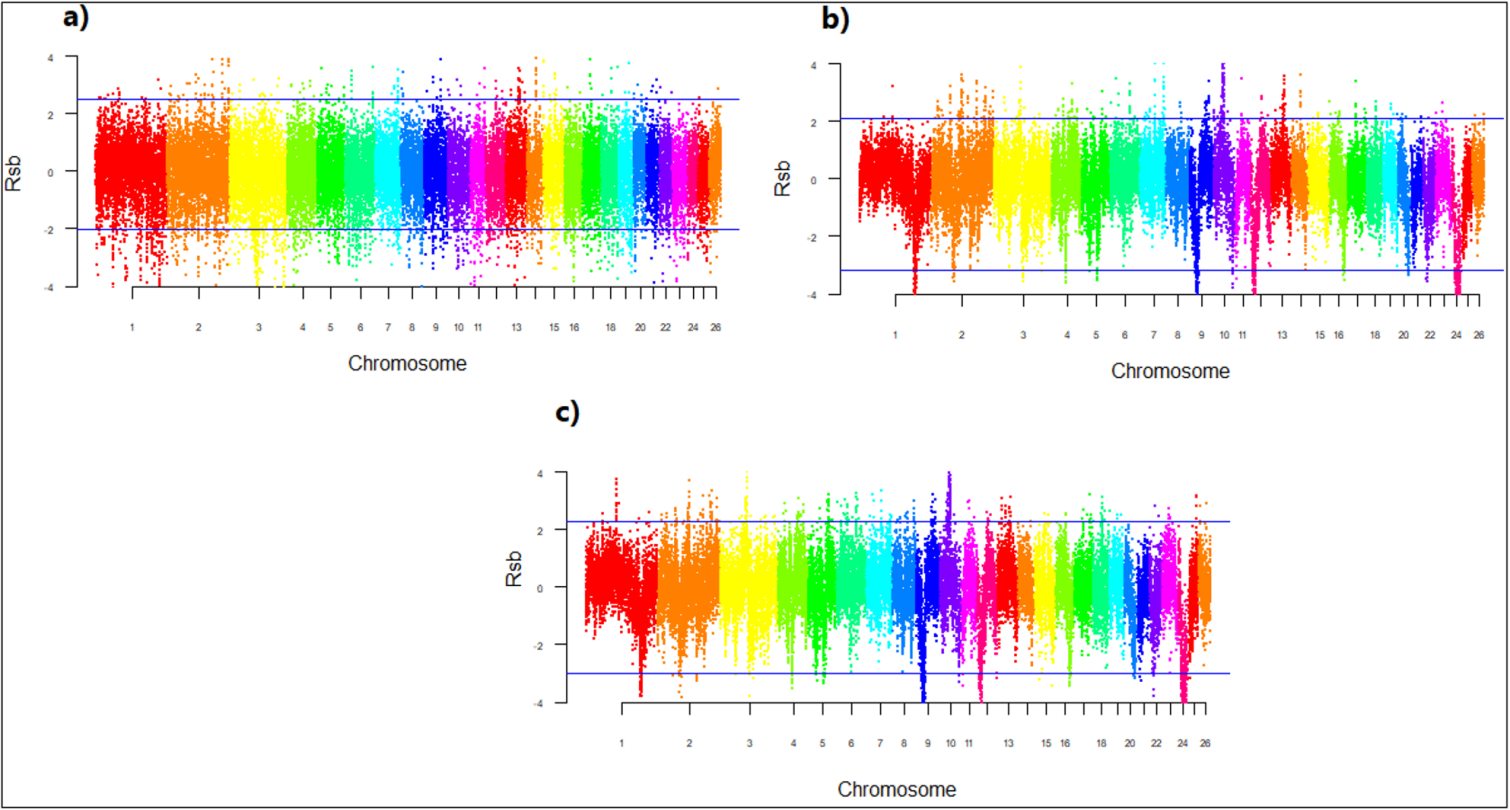
Genomic distribution of standardized haplotype differentiation (Rsb) scores on 26 sheep autosomes pairwise: (**a**) IR and IN breeds, (**b**) IR and AF breeds, (**c**) IN and AF breeds. For breed abbreviations, see Table 1.

The Rsb scores were calculated for haplotype frequencies Fig. 6. The top 1% of Rsb, considered as selective sweeps, identified 185 genes for (a) IR and IN breeds, 249 genes for (b) IR and AF breeds, and 233 genes for c) IR and AF breeds (Supplementary Table S3). Many candidate genes specially associated with immune response and heat stress were identified by Rsb test, such as, ATP2B1, ATP2C1, ATP6V1H, BMPR1B, PLCE1, LRP1B, CXCL1, CD19, DOCK1, DOCK4, PTPRA, MAPK3, UNC5C, ANKRD2, BBS9, NAFTC2IP, RNF139, and ZNF695 in immune system and environment adaptation, and IFT22, EIF2A, HSPB1, TBX6, TBX21, GNA12, BMP7, IL16, IL27, IL4R, and IL21R in heat stress. Furthermore, HOXD1, HEXD2, and MTX2 affect the horn traits,and PRLP, TBC1D10B, TMEM151A, TMEM65, TMEM225B, BMPRIB, and BMP7 genes associated with fertility traits were detected as candidate genes using Rsb.

**Figure 7:**
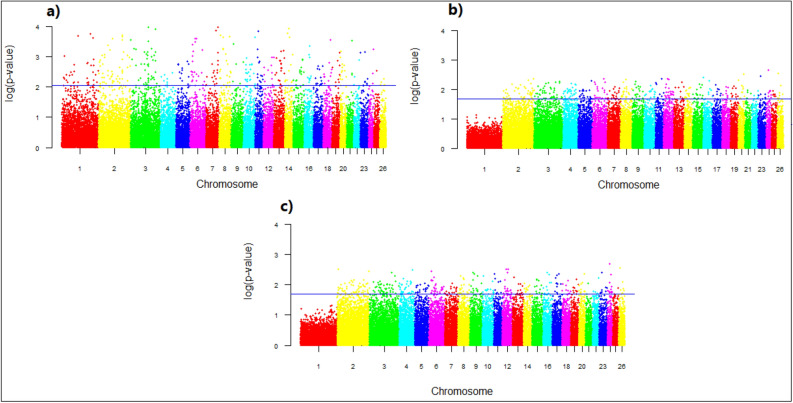
Genomic distribution of single marker statistic (FLK) scores on 26 sheep autosomes pairwise: (**a**) IR and IN breeds, (**b**) IR and AF breeds, (**c**) IN and AF breeds. For breed abbreviations, see Table 1.

The -log(p-value) values of 39,348 SNPs for the FLK test are presented in Fig. 7. Based on the -log(p-value), a total of 244 genes as top 1% candidates for selective signals were detected in (a) IR and IN breeds,265 genes in (b) IR and AF breeds; and 247genes in (c) IN and AF breeds (Supplementary Table S1). Several candidate genes identified using FLK test are related to economic traits, for example, FABP3, SLC27A6, ACP7, ANXA13, HEATR5B, ADCY2, BRD4, BRD8, HDAC9, TTC8, TTC23, WDR7, WDR31, WDR70, and POU6F1 related to milk traits as well as HERC2, FAM169A, FTO, TP73, GRM2, GRM3, and UBR2 for body weight and growth traits. Several candidate genes related to immune system and climate adaptation were detected by FLK, such as, ADAMTS6, ADAMTS20, ARHGAP26 GALNT6, GALNT13, GALNT18, ATP2C1, ADAM19, MAPK10, MAGI2 ADAM33, LRP1B, CXCL14, TMPRSS3, TRPC4, NBEAL1, CD34, COL12A1, PCDH15, DOCK1, DOCK4, DOCK10, UNC5B, BBS9, CDH6, CHD3, IRF6, ITGA, LRP1B, NAFATC1, RNF26, ZNF609, and ZNF692.

**Figure 8:**
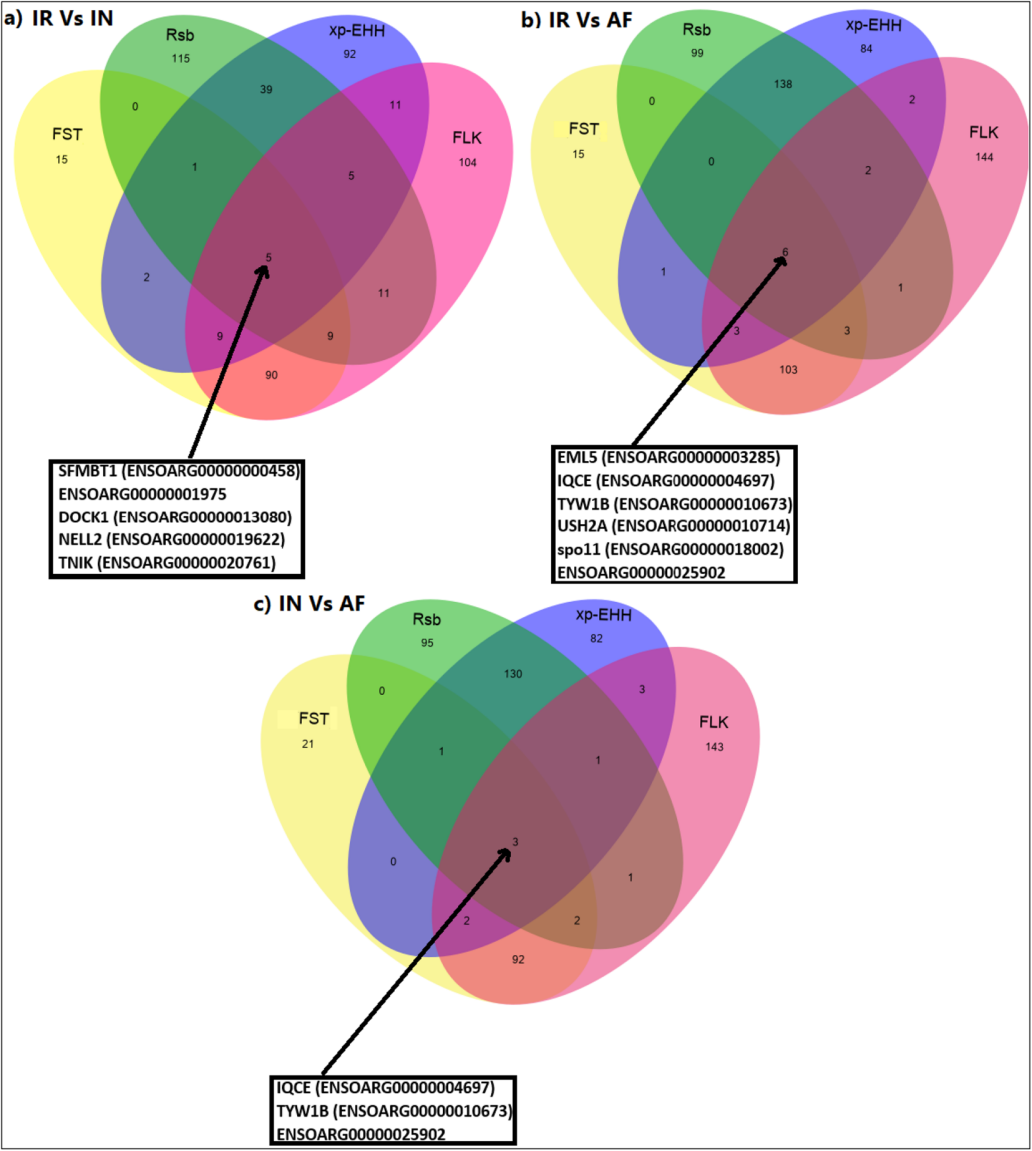
Venn diagram showing the unique and shared candidate genes for FST, Rsb, xp-EHH, and FLK tests on: (**a**) IR vs. IN, (**b**) IR vs. AF, and (**c**) IN vs. AF sheep breeds. For breed abbreviations, see Table 1.

The F_ST_ and FLK tests with average 128 and 252 genes showed the minimum and maximum captured genes among these four tests. Furthermore, five, six and three concordant genomic regions for (a) IR and IN breeds, (b) IR and AF breeds, (c) IN and AF breeds were identified by F_ST_, xp-EHH, Rsb, and FLK tests as candidates for selection signals, respectively Fig. 8. These overlapping candidate genes for (a) IR and IN breeds include the following genes,Scm-like with four MBT domains protein 1 (SFMBT1) on chromosome 19, plays a role during spermatogenesis. Dedicator of cytokinesis protein 1(DOCK1) on chromosome 22, has an essential role in embryonic development and involved immune response, Neural EGFL like 2 (NELL2) on chromosome three, involved in involved in pubertal development. NCK-interacting protein kinase (TNIK) on chromosome one, the protein encoded by this gene plays important role in embryonic development, especially during the early embryo to blastocyst stages, participates in the regulation of the inflammatory response against infections.

The overlapping candidate genes for (b) IR and AF breeds include, Echinoderm microtubule-associated protein-like 5 (EML5) on chromosome seven, may change the assembly dynamics of microtubules to make microtubules are slightly longer but more dynamic and it is possible that EML5 plays a role during neuronal development in the regulation of cytoskeletal rearrangements, IQ domain-containing protein E (IQCE) on chromosome 24, involved in body development, TRNA-YW Synthesizing Protein 1 Homolog B (TYW1B) on chromosome 24, influenced on the wybutosine biosynthesis pathway. Usherin (USH2A) on chromosome 12, may be involved in the function of synapses and plays an important role in the development and maintenance of cells in the inner ear and retina. SPO11 initiator of meiotic double-stranded breaks (SPO11) on chromosome 13, involved in the production of double-strand breaks (DSB) of DNA and it is specifically involved in the growth of the testis, maintenance of the male germ line, and maturation of sperm. Three overlapping candidate genes for c) IN and AF breeds were detected; the IQCE, TYW1B, and an unknown gene with Ensemble number (ENSOARG00000025902) which all of these three genes were detected before (IR vs. AF).

**Figure 9:**
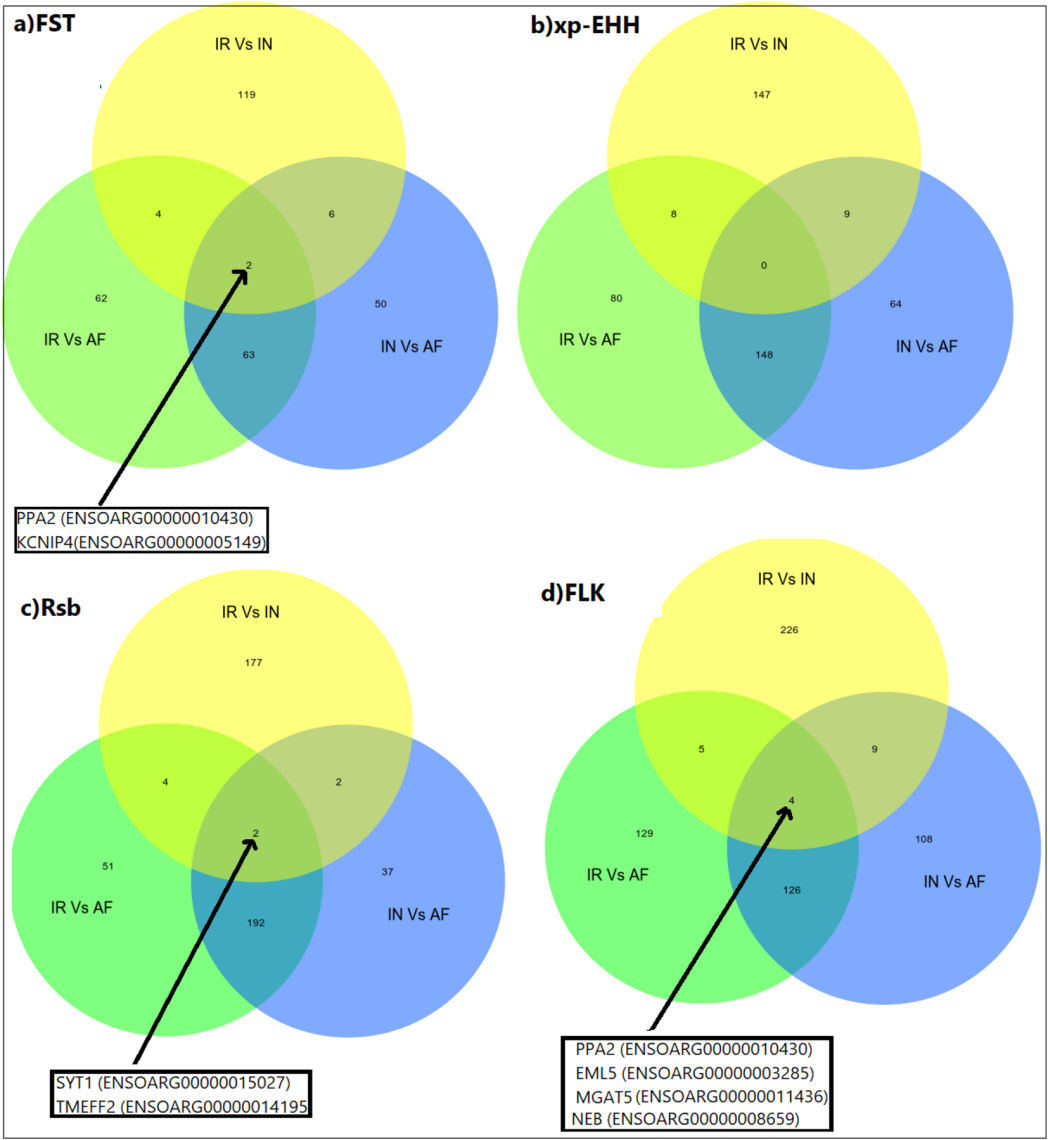
Venn diagram showing the unique and shared candidate genes for IR vs. IN, IR vs. AF, and IN vs. AF data on: (**a**) FST, (**b**) xp-EHH, (**c**) Rsb, and (**d**) FLK tests. For breed abbreviations, see Table 1.

We also detected overlapping candidate genes for IR vs. IN, IR vs. AF, and IN vs. AF data on the F_ST_, xp-EHH, Rsb, and FLK tests Fig. 9. For the F_ST_ test PPA2, involved in the immune system, and KCNIP4 plays important role in heart performance and it is related to skeletal muscle growth and also immune response. SYT1, associated with feeding behavior traits such as residual feed intake and TMEFF2, involved in a wide range of traits such as,immune response, milk production and sperm morphology, were detected as overlapping candidate genes for the Rsb test. For the FLK test, PPA2, EML5 genes, which have been found in the previous tests, MGAT5, associate with dry matter intake and NEB, involved in environment adaptation, were detected as overlapping candidate genes on the three different data. We did not find any overlapping candidate genes on the all data by the xp-EHH test.

Biological enrichment analysis of significant biological processes for candidate genes under positive selective pressure revealed 26 Gene Ontology (GO) terms Table 2. These GO terms reflected protein function and biosynthetic processes, including TNIK and DOCK1 genes associated with cytoskeleton organization (GO:0007010) and six other GOs related to the TNIK gene. Four other GOs were associated with DOCK1. The SFMBT1 gene associated with negative regulation of transcription (GO:0035556). Seven GOs were associated with the spo11. Four GOs were related to the USH2A gene. Finally, tRNA processing (GO:0008033) was associated with the TYW1B gene.

**Table 2:** Breed biological process of common candidate genes under selective pressure for F_ST_, Rsb, xp-EHH, and FLK tests on: (a) IR vs. IN, (b) IR vs. AF, (c) IN vs. AF.

Group	Biological process		Genes	p. adjust
a	Cytoskeleton organization	GO:0007010	DOCK1,TNIK	0.0027
	Regulation of dendrite morphogenesis	GO:0048814	TNIK	0.021
	Actin cytoskeleton reorganization	GO:0031532	TNIK	0.044
	Hematopoietic progenitor cell differentiation	GO:0002244	DOCK1	0.046
	Small GTPase mediated signal transduction	GO:0007264	DOCK1	0.046
	Protein localization to plasma membrane	GO:0072659	TNIK	0.046
	Cell migration	GO:0016477	DOCK1	0.046
	Positive regulation of protein phosphorylation	GO:0001934	TNIK	0.046
	Protein auto phosphorylation	GO:0046777	TNIK	0.046
	Positive regulation of GTPase activity	GO:0043547	DOCK1	0.065
	Intracellular signal transduction	GO:0035556	TNIK	0.081
	Negative regulation of transcription	GO:0045892	SFMBT1	0.097
b	Reciprocal meiotic recombination	GO:0007131	SPO11	0.007
	Sensory perception of light stimulus	GO:0050953	USH2A	0.007
	Synaptonemal complex assembly	GO:0007130	SPO11	0.007
	Male meiosis I	GO:0007141	SPO11	0.007
	DNA metabolic process	GO:0006259	SPO11	0.007
	Ovarian follicle development	GO:0001541	SPO11	0.007
	Oogenesis	GO:0048477	SPO11	0.007
	Synapsis	GO:0007129	SPO11	0.007
	Photoreceptor cell maintenance	GO:0045494	USH2A	0.007
	Establishment of protein localization	GO:0045184	USH2A	0.007
	tRNA processing	GO:0008033	TYW1B	0.011
	Spermatid development	GO:0007286	SPO11	0.013
	Sensory perception of sound	GO:0007605	USH2A	0.020
c	tRNA processing	GO:0008033	TYW1B	0.003

**Figure 10:**
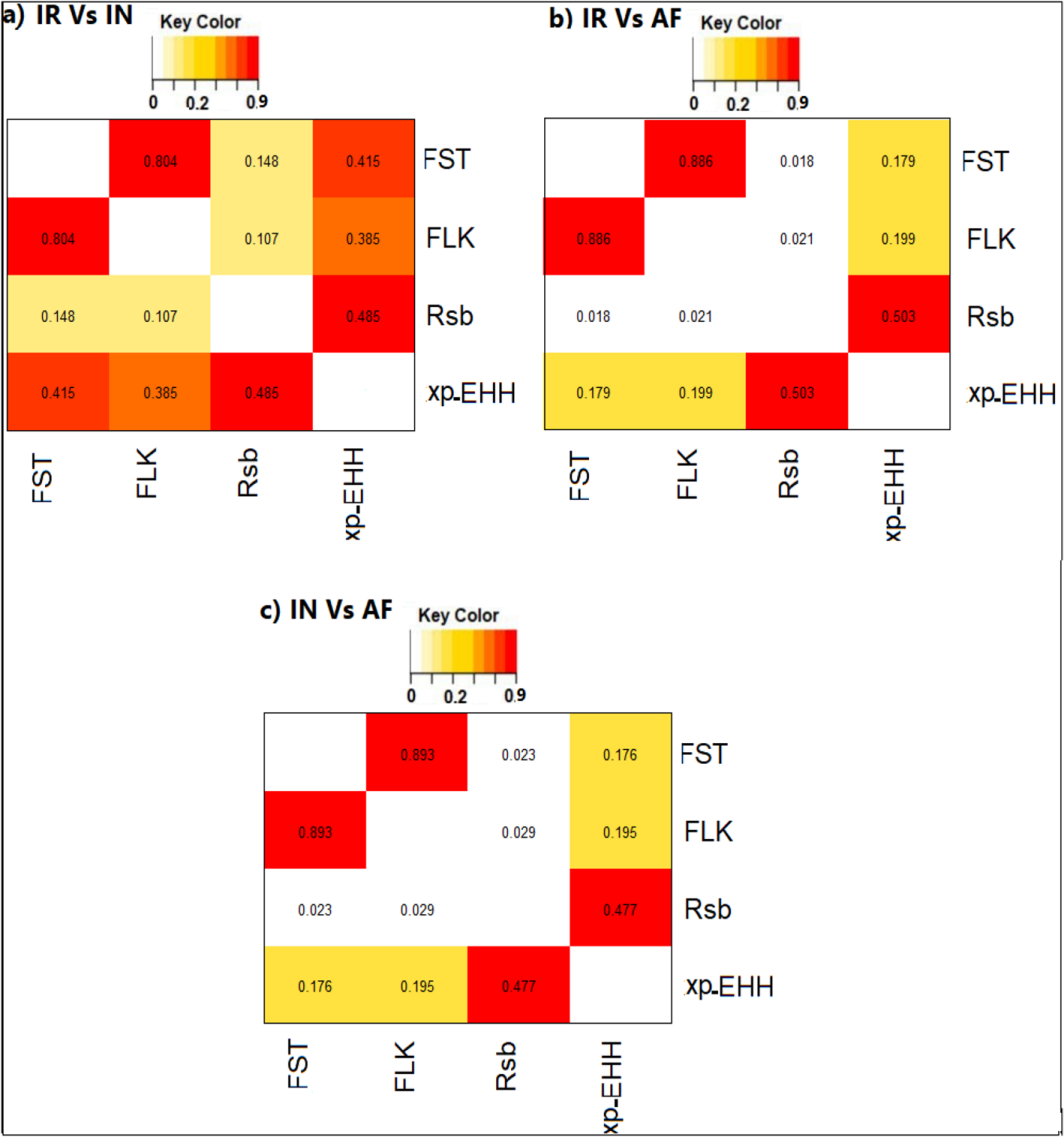
Absolute correlation among different methods used to detect selective sweeps on: (**a**) IR vs. IN, (**b**) IR vs. AF, and (**c**) IN vs. AF sheep breeds. For breed abbreviations, see Table 1.

Absolute correlation coefficients among these four tests on (a) IR vs. IN, (b) IR vs. AF, and (c) IN vs. AF sheep breeds showed the maximum correlation between F_ST_ and FLK on the all comparisons (average: 0.861) and the minimum correlation between FLK and Rsb on IR vs. IN (0.107) and F_ST_ and Rsb on IR vs. AF and IN vs. AF data (average: 0.021) Fig. 10.

## Discussion

The present study investigates the genetic diversity and selective sweeps of 14 sheep breeds from Iran, Afghanistan, India, and Bangladesh. The selective sweeps were studied using the F_ST,_ FLK, xp-EHH and Rsb statistical methods on the three cluster of breeds (IR, IN, and AF). Our goal in the current study was to search the genomes of these indigenous sheep breeds to highlight genetic variants that can be used in developing next-generation productive breeds, better suited to diverse Iran environments, in a comparative scale with Indian, Bengal, and Afghan sheep breeds. Furthermore, the other goal was using four comparable selective sweeps tests to cover all the regions of the genomes and capture maximum candidate genes, as well as review their biological function. The results showed that these breeds' genomes contain multiple regions under selection. These regions contain well-known economic trait-related candidate genes. This could help sheep breeders to: (1) improve adaptation in extant breeds; (2) develop native breeds that are better adapted to local agro-climatic conditions; (3) launch future research work on the genomes of Iranian, Afghan, Indian, and Bengal sheep, and highlight essential genetic variants or haplotypes that can be used in the production of higher productivity and efficiency next-generation breeds, better adapted to various Iranian environments, on a comparative scale with Afghan, Indian, and Bengal sheep breeds.

### Genetic relatedness and geographic origin

We demonstrated that the IR sheep breeds are genetically distinct from the breeds of IN and AF. Based on their geographic origins, the studied sheep breeds are well clustered. We categorized the IR, IN and AF breeds into three phylogeographic clades. Close connections between breeds originating in the same geographical region have been found. In fact, phylogenetic analysis showed a close genetic relationship among the IR breeds. These breeds are from cold and temperate climates of Iran. On the other hand, the IN breeds showed a closer relationship among BGA, BGE, and GAR breeds from the eastern region of India and Bangladesh. In contrast, IDC from the western peninsular region and CHA from northern Himalayan part of India formed two distinct sub clusters.

Furthermore, the AF cluster showed an IBL sub-cluster and a compact sub-cluster of three Afghan breeds, indicating a closer relationship among Afghan breeds and their genetic distance from IBL.

These findings are consistent with previous research on sheep^38^^,^^39^^,^^3^^,^^40^, which showed that individuals were separated by global population structure patterns according to their geographical origin.

In accordance with previous findings^2^^,^^41^^,^^38^ PCA results demonstrated that the genetic variation was associated with the separation among sheep breeds from different parts of the world. This was further supported by neighbour-joining tree analysis revealing that the population was split according to geographic origin (IR, IN, and AF). Population structure analyses of the IR, IN, and AF breeds clearly reflected the geographic distribution at PC1 and the separation of northern from southern breeds at PC3.

### Admixture and phylogenetic patterns

In accordance with the previous analyses, admixture results confirmed that the first few ancestral breed components (K = 2 to K = 5) were related to the geographic origins. High levels of breed admixture were detected among the Iranian (IR and IBL) breeds, and also among the IN breeds. A significant IR ancestry is observed in the CHA breed at K = 4, which probably due to the same climate between the CHA from Kashmir and the IR breeds from the northwest of Iran Table1, and also due to historical ties and neighborliness between Iran and India, especially in the Kashmir region. It is possible that the IR breeds and CHA have common ancestors. However, low levels of admixture events among the breeds originating from the different geographical regions were detected. For example, although the GAR and BGA from India and Bangladesh have a common breed name (Garole), they separated at k = 5 ancestral breed components, while BGA and BGE which are known as two different breeds in Bangladesh showed more relationship and they have been separated at k = 9 which confirm the effect of geographic origin in breeds separation. Admixture results confirmed genetic divergence identified through the neighbor-joining and PCA.

Inference based on population neighbour-joining trees based on genome-wide allele frequencies clustered the breeds into three monophyletic clades according to the geographical origin. The deepest population split among the AF breeds separated IBL from the other AF breeds. Among the IN breeds, IDC and CHA showed deeper population splits, in line with geographic clades detected by the PCA and admixture analysis. These results support the previous findings^2^^,^^41^^,^^38^.

### Runs of homozygosity

The history of inbreeding within a population can be estimated from the length distribution of ROH segments^42^. We estimated F_ROH_ to study genomic inbreeding. The average F_ROH_ levels estimated for the breeds was (0.09), which was almost the same as the previous study^3^. In the studied breeds, the range of FROH recorded 0.008 to 0.5, was higher than the previous maxima but the observed minima were consistent with previous findings. Mastrangelo et al.^43^ reported the range of F_ROH_ from 0.016 to 0.099 in domestic sheep breeds.

The IBL and IN breeds had the largest number of ROHs, and therefore showed highest FROH levels, indicating the relationship between the number of ROH segments and FROH levels^3^. The results of ROH segment also showed that more than 93% of the ROH segments were shorter than 8 Mb, which indicated older events of inbreeding and a board effective population size of sheep flocks^43^. The IN breeds displayed a higher FROH variance than the IR and AF breeds which could indicate more effective population size variation in the IN breeds. Inbreeding and extended ROH segments can be increased by small population size and intense selection, thereby continuing to express the deleterious alleles^44^.

### Genome-wide selective sweeps

The ability of specific genomic regions to detect selective sweeps depends on the selection of analytical tools appropriate to the biological situation but no single method can detect selective sweeps that are both starting and nearly completed. However, combining several tests increases significantly the power to recognize the region selected^6,7^. Therefore, we used F_ST_, FLK, xp-EHH, and Rsb test statistics to detect genome-wide selective sweeps in (a) IR and IN breeds, (b) IR and AF breeds, (c) IN and AF populations. F_ST_ was first implemented to measure the degree of genetic differentiation between populations based on variations in allele frequency^45^. The genomic variation information is provided by F_ST_ at a locus between the populations compared to within the populations. Therefore, the F_ST_ is an evidence of selection: high F_ST_ values indicate positive local adaptation^46^. The older selection events between populations are expected to be identified by F_ST_^47,48^. The xp-EHH test is an extension of EHH^31^, that incorporates information on the relationship between an allele's frequency and LD measurements with neighboring alleles. Therefore, this test may provide maximal statistical power and low ascertainment bias sensitivity^33^. The Rsb test is population comparison test to identify selective sweeps^33^. The test is based on the same idea as the XP-EHH, identifies loci similar to the XP-EHH test under selection, but can be implemented with unphased data^28^. Generally, the xp-EHH and Rsb tests are used to detect recent positive selection within population and between-populations, respectively^9^. The FLK (extended Lewontin and Krakauer test) test is based on the assumption that two new populations are formed by the splitting of a population,calculates a statistic of population differentiation, which incorporates a matrix of kinship describing the relationship between populations^27,28^. For each SNP, the FLK test calculates a global F_ST_, but allele frequencies are first rescaled using a matrix of population kinship. This matrix, which is estimated from the genome-wide data observed, measures the amount of genetic drift that can be predicted along all branches of the population tree under neutral evolution^27^. Therefore, the integration of these four complementary statistical tests provides a valuable tool for detecting, with greater confidence, positive selection of genomic regions.

For F_ST_ and FLK, only the top 1% Z(F_ST_) values and the top 1% -log(p-value) were considered, respectively to be representing selective sweeps as recommended in previous studies^29^^,^^40^.

Analyses of selective sweeps were reported for several international sheep populations from several countries, including China^49^, Europe^41,50^, Russia^51^, Egypt^52^, Brazil^53^, and New Zealand^54^. Furthermore, several studies of selective sweeps on sheep carried out using different tests, including the FLK and hapFLK^2,55^, hapFLK, FLK, F_ST_, and hapF_ST_^10^, REHH and xp-EHH^56^, F_ST_ and hapFLK^49^, F_ST_ and iHS^52^, F_ST_, Rsb, and iHS^53^.

This study, using F_ST_, xp-EHH, Rsb, and FLK, detected on average 128, 207, 222, and 252 genomic regions as candidates for selective sweeps, respectively. Although the selected candidate regions are narrow and are distributed across different chromosomes, however for F_ST_ and FLK tests, chromosome 1 showed a low value for IR vs. AF and IN vs. AF comparisons which may indicate the genome of two populations are the same in this region and many common genes were expected to be fixed in both populations^48^, Figs. 4 and 7. Several of these genes encode economically important traits. For example, genes that have directly or indirectly influence traits for adaptation to hot arid conditions and heat tolerance (TRHDE, IL4R,IL21R, and SLC4A4), which are reported as candidate genes involved in heat tolerance on sheep^57^. The heat shock protein B1 (HSPB1) gene which expresses both at mRNA and protein levels under heat stress on poultry^58^, reported in sheep^59^, and cattle^60^. All of these candidate genes were detected in IR vs. AF, and vs. AF clusters, where the AF breeds are common (Supplementary Tables S1, S2, and S3). This indicates that the AF breeds, which are from a hot dry climate, have undergone selection for heat tolerance.

Many of the candidate genes identified in this study are effective in genetic resistance to disease, immune response and climate adaptation, which indicates differential selections among the studied breeds. Since genetic resistance against diseases and harsh environmental conditions are important characteristics of indigenous animal breeds, the identification of a large number of genes in this study points toward the associated genes have been under selection pressure over time due to the natural selection of immune response traits^61,62^. For example, we detected the DOCK family (DOCK1, DOCK4, DOCK10)^63,64^, ZNF family (ZNF572, ZNF655, ZNF609, ZNF692, and ZNF789)^65,66^, ATP family (ATP2A1, ATP2B1, and ATP2C1)^67^, TMEFF2^68^, CXCL1^69^, PCDH15^69^, and (COL12A1, COL15A1, COL27A1)^69^, VPS16 and PTPRA^70^, PLCE1^71^, ATAMTS20^72^, TPRC4^73^, candidate genes involved in the immune response and climate adaptation (Supplementary Tables S1, S2, and S3). Almost all these genes were detected in all three clusters, a (IR and IN), b (IR and AF), and c (IN and AF), which may indicate genetic resistance and high immune response against diseases and harsh environmental conditions in these native breeds.

We detected some genes involved in production traits and indirectly related to climate adaptation, such as, FABP3. Calvo et al.^74^ showed linkage disequilibrium between FABP3 gene and quantitative trait loci (QTL) for milk fat content. Other related milk traits candidate genes included the LRP1B and CNTN4 which previously reported on sheep^75^ and cattle^76^. The ITPR2 and SLC27A6 are also two examples of important candidate genes detected by Li et al.^75^ on sheep and both have been proposed to be candidate genes for milk and fat production in cattle and indirectly involved in climate adaptation^77^^,^^78^. All of these genes were found in b (IR and AF), and c (IN and AF) clusters, indicating AF breeds may be under selection pressure related to milk traits but it needs further research to conclude. (Supplementary Tables S1, S2, and S3).

We found several candidate genes involved in body weight and growth traits specially post-weaning gain in all population clusters, such as the TRHD, UBR2, GRM2, GRM3 which also related to climate adaptation indirectly^79^^,^^80^.

Furthermore, 11 overlapping candidate genome regions were detected for F_ST_, Rsb, xp-EHH, and FLK tests on: (a) IR vs. IN, (b) IR vs. AF, and (c) IN vs. AF sheep breeds Fig. 8. The number of overlapping unique candidate genes are consistent with the previous results using F_ST_, Rsb, and iHS tests^81^, F_ST_, xp-EHH, and iHS tests^48^, ROH, F_ST_, and xp-EHH^82^.

Four of the genes (DOCK1, TYW1B, USH2A, and TNIK) play important roles in resistance against diseases and climate adaptation. DOCK1 located on chromosome 22 is involved in immune response^63,64^. TNIK on chromosome 1 plays several functions in embryonic development, especially during the early embryo to blastocyst stages, participates in the regulation of the inflammatory response against infections^83^, TYW1B on chromosome 24 influences artery disease and blood pressure in human^84^, USH2A on chromosome 12 may be involved in the function of synapses and plays an important role in the development and maintenance of cells in the inner ear and retina^85^.

In total, seven unique candidate genes were detected for IR vs. IN, IR vs. AF, and IN vs. AF comparisons by F_ST_, Rsb, and FLK analysis, but no overlapping candidate gene was found for the xp-EHH method Fig. 9.

PPA2 on chromosome 6 is associated with immune response and disease resistance in cattle^86^. KCNIP4 gene on chromosome 6 is directly involved in processes related to muscle growth and fat deposit and indirectly climate adaptation in sheep^87^ and was reported in cattle involved in bovine growth and calcium metabolism^88^. SYT1 gene on chromosome 3 is associated with feeding behavior traits related to local adaptation^89^, and TMEFF2 gene on chromosome 2 is involved in a wide range of traits such as, immune response, milk production and, sperm morphology^68,90^. For the FLK test, PPA2, EML5, MGAT5, and NEB genes were detected, which PPA2 and EML5 have been found in the previous tests Fig. 9. NEB gene on chromosome 2 is involved in environmental adaptation. Among 1262 selected genomic regions reported by Yudin and Larkin^91^, only NEB gene was a shared candidate gene among cattle, sheep, mammoth, polar bear, and whale genomes.

GO classifications of the candidate genes were performed to enable a better understanding of their molecular functions. Based on the GO biological process (BP) for a significant threshold (p ≤ 0.05), we implemented the GO on 11 overlapping candidate genes. Only six genes (TNIK, DOCK1, SFMBT1, SPO11, USH2A, and TYW1B) associated with the 26 GO terms were identified. In total 11 GOs were related to TNIK, USH2A, TYW1B, and DOCK1, which are associated with local adaptation (resistance against diseases).

In confirmation of our results, Nie et al.^92^, reported different GO terms associated with the TNIK gene in human^92^. Four other GOs associated with DOCK1. DOCK family genes have several biological functions^63,64^.

Absolute correlation among the F_ST_, FLK, xp-EHH, and Rsb tests were calculated Fig. 10. The xp-EHH, and Rsb are based on the frequency of extended haplotypes between two populations^33,34^, whereas F_ST_ and FLK are based on allele frequencies^6,37^. So as expected, maximum correlations were observed between F_ST_ and FLK, as well as between xp-EHH and Rsb. On the other hand, we detected minimum correlations between haplotype based tests (xp-EHH, and Rsb) and allele based tests (F_ST_ and FLK). These findings are consistent with the previous reports^8^^,^^28^.

## Conclusions

Our results showed the population structure and selective candidate genomic regions of the 14 indigenous sheep breeds from Middle East and South Asia. This information would be valuable in future study on genetic basis for local adaptation of indigenous breeds. In F_ST,_ FLK, xp-EHH, and Rsb complementary statistical tests, some candidate genomic regions under selective pressure were detected in indigenous sheep breeds and these candidate genomic regions may facilitate identification of the underlying genes and possible exploitation in future sheep breeding.

## Supplementary Information

Supplementary Information

## Data Availability

Genotype data from the sheep breeds (Afshari, Moghani, Qezel, Bangladeshi Garole, Bangladesh East, Indian Garole, Changthangi, and Deccani) are available through the Sheep HapMap project^11^. The ZEL, Lori-Bakhtiari, Iranian Balochi, Arabi, Afghan Balochi, and Gadik breeds data are part of the Iranian national genetic evaluations of economic traits conducted at the Animal Breeding Center of Iran. Any request for data should be addressed to the corresponding author.
