# Supplementary material for: Genomic scans for selective sweeps through haplotype homozygosity and allelic fixation in 14 indigenous sheep breeds from Middle East and South Asia
Source: Sci Rep. 2021 Feb 2;11:2834. doi: 10.1038/s41598-021-82625-2 (PMC7854752; doi:10.1038/s41598-021-82625-2)

Genomic scans for selective sweeps through haplotype homozygosity and allelic fixation in 14 indigenous sheep breeds from Middle East and South Asia

Running Head: Selection signatures in indigenous sheep breeds

Sirous Eydivandi[1,2], Mahmoud Amiri Roudbar [3], Mohammad Osman Karimi[4], and Goutam Sahana[2]

[1] Department of Animal Science, Behbahan Branch, Islamic Azad University, Behbahan, Iran

[2] Center for Quantitative Genetics and Genomics, Faculty of Technical Sciences, Aarhus University, 8830 Tjele, Denmark

[3] Department of Animal Science, Safiabad-Dezful Agricultural and Natural Resources Research and Education Center, Agricultural Research, Education & Extension Organization (AREEO), Dezful, Iran

[4] Department of Animal Science, Faculty of Agriculture, Herat University, Herat, Afghanistan

*Correspondence:

Sirous Eydivandi

sirous.eidivandi@qgg.au.dk

sirouseidivandi@gmail.com

Supplementary Table S1. Top 1% genes as candidates for selective signals using Z(FST) test on 26 sheep autosomes for: a) IR and IN breeds, b) IR and AF breeds, c) IN and AF breeds.

a

Gene.stable.ID	Gene.name	Gene.start	Gene.end	Chr.name
ENSOARG00000000493		18080828	18124219	1
ENSOARG00000000515	ST3GAL3	18120931	18347967	1
ENSOARG00000001069	GOLIM4	217358248	217440402	1
ENSOARG00000008995	DNAJC13	255172444	255307652	1
ENSOARG00000009414	ATP2C1	256985186	257068039	1
ENSOARG00000009979		258241417	258581681	1
ENSOARG00000010761	TMPRSS3	260732616	260756933	1
ENSOARG00000016027	USP25	139564863	139699601	1
ENSOARG00000017549	DPYD	73433343	74364967	1
ENSOARG00000020761	TNIK	214087639	214487796	1
ENSOARG00000021060	RORC	100648111	100672007	1
ENSOARG00000021065		100680890	100831184	1
ENSOARG00000025511		17971643	18117954	1
ENSOARG00000002545	EPB41	237305059	237487293	2
ENSOARG00000006134	AMBP	9583152	9609521	2
ENSOARG00000008634		31239795	31476634	2
ENSOARG00000008659	NEB	156650358	156853665	2
ENSOARG00000008930	RIF1	156861839	156919308	2
ENSOARG00000009251	DNAI1	36819526	36890350	2
ENSOARG00000009977	ADAM7	41012453	41069859	2
ENSOARG00000011436	MGAT5	175253426	175595136	2
ENSOARG00000012027		52695717	52765806	2
ENSOARG00000014195	TMEFF2	193228532	193510876	2
ENSOARG00000015036	HMBOX1	102231442	102383638	2
ENSOARG00000016867	ITGA4	127163497	127254486	2
ENSOARG00000017979	NBEAL1	204016478	204166655	2
ENSOARG00000022859	U6	91862325	91862426	2
ENSOARG00000025733		954711	1233648	2
ENSOARG00000025798		114046190	114223286	2
ENSOARG00000001844	VRK2	65858703	65955917	3
ENSOARG00000002277		1442982	2818577	3

b

Gene.stable.ID	Gene.name	Gene.start	Gene.end	Chr.name
ENSOARG00000004384	RPS6KA1	239443298	239467559	2
ENSOARG00000007715	KDM1A	242660932	242714290	2
ENSOARG00000007814	SYK	25701083	25759453	2
ENSOARG00000008084	WNK2	28059615	28213657	2
ENSOARG00000009010	UBQLN1	35845653	35897664	2
ENSOARG00000010998	COL15A1	48487007	48588324	2
ENSOARG00000011504	ZCCHC7	51076086	51317595	2
ENSOARG00000015710	HERC2	112478388	112720769	2
ENSOARG00000025847		215173693	215317344	2
ENSOARG00000002894	RTN4	68869891	68943954	3
ENSOARG00000007419	ABL1	5731767	5765473	3
ENSOARG00000008162	MAP4K3	84680681	84866029	3
ENSOARG00000008932	ANO2	208302342	208641090	3
ENSOARG00000010376		91050821	91234495	3
ENSOARG00000011771	TESPA1	164960462	164984444	3
ENSOARG00000014173	ANO4	169622065	170061610	3
ENSOARG00000014722	UTP20	170228637	170315908	3
ENSOARG00000015343	GNPTAB	170615182	170667987	3
ENSOARG00000017314	GALNT6	134693949	134722893	3
ENSOARG00000018354		32668067	32874601	3
ENSOARG00000019050	NCF4	180081443	180103379	3
ENSOARG00000019095	CSF2RB	180166727	180179868	3
ENSOARG00000019458	BABAM2	34767758	35168619	3
ENSOARG00000019564	TMTC1	184510212	184836655	3
ENSOARG00000019999	KRAS	189578775	189614533	3
ENSOARG00000001140		112262485	112438114	4
ENSOARG00000002931	DOCK4	56152727	56624017	4
ENSOARG00000006085		95239604	95287750	4
ENSOARG00000006901	THSD7A	19499637	19777463	4
ENSOARG00000007096	PDE1C	64359063	64669363	4
ENSOARG00000008903		66590322	66929661	4

c

Gene.stable.ID	Gene.name	Gene.start	Gene.end	Chr.name
ENSOARG00000004384	RPS6KA1	239443298	239467559	2
ENSOARG00000007440	GRIN3A	21252157	21459411	2
ENSOARG00000007814	SYK	25701083	25759453	2
ENSOARG00000008084	WNK2	28059615	28213657	2
ENSOARG00000009010	UBQLN1	35845653	35897664	2
ENSOARG00000009898	GTDC1	164635825	164985339	2
ENSOARG00000010706	UBXN4	173931295	173953446	2
ENSOARG00000010998	COL15A1	48487007	48588324	2
ENSOARG00000011027	RAB3GAP1	174458565	174694534	2
ENSOARG00000011436	MGAT5	175253426	175595136	2
ENSOARG00000012588	CARNMT1	61274631	61304613	2
ENSOARG00000015655	OCA2	112126827	112463751	2
ENSOARG00000015710	HERC2	112478388	112720769	2
ENSOARG00000016969		129907617	130149171	2
ENSOARG00000019232	ERBB4	212081308	212484401	2
ENSOARG00000020437	DOCK10	225910579	226106181	2
ENSOARG00000025766		50388292	50508316	2
ENSOARG00000002894	RTN4	68869891	68943954	3
ENSOARG00000007419	ABL1	5731767	5765473	3
ENSOARG00000009846	GPATCH11	86879693	86887627	3
ENSOARG00000012360	ELK3	165522205	165579438	3
ENSOARG00000014722	UTP20	170228637	170315908	3
ENSOARG00000015054	PPP1R12A	115651729	115819054	3
ENSOARG00000015343	GNPTAB	170615182	170667987	3
ENSOARG00000018354		32668067	32874601	3
ENSOARG00000019050	NCF4	180081443	180103379	3
ENSOARG00000019095	CSF2RB	180166727	180179868	3
ENSOARG00000019300	PFKM	138238132	138280543	3
ENSOARG00000019458	BABAM2	34767758	35168619	3
ENSOARG00000019622	NELL2	141536582	141928837	3
ENSOARG00000019909	ITPR2	187771324	188372241	3

ENSOARG00000015027	SYT1	114906496	115404501	3	ENSOARG00000013495		2255574	2448893	4	ENSOARG00000019999	KRAS	189578775	189614533	3
ENSOARG00000015359	LRRIQ1	121148785	121363705	3	ENSOARG00000013517		3629296	3769877	4	ENSOARG00000002931	DOCK4	56152727	56624017	4
ENSOARG00000015702	EPYC	127257338	127295253	3	ENSOARG00000014780	ELAPOR2	33294019	33512918	4	ENSOARG00000006085		95239604	95287750	4
ENSOARG00000016814	TXNRD1	172982661	173042110	3	ENSOARG00000014903	GRM3	33520799	33628582	4	ENSOARG00000006427	PLXNA4	95788312	96204055	4
ENSOARG00000017296	NUAK1	174550166	174621290	3	ENSOARG00000017010	AKAP9	9183142	9351553	4	ENSOARG00000008198	AGMO	23650918	24047746	4
ENSOARG00000017344	BIN2	134750526	134787072	3	ENSOARG00000017240	HECW1	77592533	77851748	4	ENSOARG00000009409	SNX13	26371787	26467169	4
ENSOARG00000019300	PFKM	138238132	138280543	3	ENSOARG00000017420	GLI3	79029288	79131016	4	ENSOARG00000009641	HDAC9	27257256	27548397	4
ENSOARG00000019311	SENP1	138282207	138331370	3	ENSOARG00000017524	MAGI2	42637311	43299968	4	ENSOARG00000013517		3629296	3769877	4
ENSOARG00000019534	IPO8	183547488	183614465	3	ENSOARG00000018237	TCAF1	106458935	106491976	4	ENSOARG00000014026	CROT	32985882	33035073	4
ENSOARG00000019578	ANO6	140942833	141115777	3	ENSOARG00000025211		3683214	3745148	4	ENSOARG00000014903	GRM3	33520799	33628582	4
ENSOARG00000019622	NELL2	141536582	141928837	3	ENSOARG00000001932	COMMD10	35086709	35265384	5	ENSOARG00000016384	CDK14	7873876	8511553	4
ENSOARG00000019717	ADAMTS20	142961377	143166195	3	ENSOARG00000013615	FSTL4	42210002	42682576	5	ENSOARG00000017240	HECW1	77592533	77851748	4
ENSOARG00000020390	SLCO1A2	193610779	193663029	3	ENSOARG00000015922	ICAM3	12546272	12554248	5	ENSOARG00000017420	GLI3	79029288	79131016	4
ENSOARG00000020802	GRIN2B	200689548	201197462	3	ENSOARG00000017050	SLC27A6	22582095	22657624	5	ENSOARG00000025211		3683214	3745148	4
ENSOARG00000026040		174577386	174723159	3	ENSOARG00000017454	SMARCA4	13107775	13181922	5	ENSOARG00000013341		11795634	12084118	5
ENSOARG00000014574	DMTF1	33148768	33195015	4	ENSOARG00000018880	MAN2A1	105984860	106179347	5	ENSOARG00000015922	ICAM3	12546272	12554248	5
ENSOARG00000018572	CDK6	9837145	10086661	4	ENSOARG00000005149	KCNIP4	40156628	40399467	6	ENSOARG00000016502	ADGRV1	87500637	88022830	5
ENSOARG00000012274	TBXA2R	17893368	17899126	5	ENSOARG00000006066	JAKMIP1	102735767	102808907	6	ENSOARG00000017050	SLC27A6	22582095	22657624	5
ENSOARG00000016418		20379609	20597764	5	ENSOARG00000010430	PPA2	19864955	19945670	6	ENSOARG00000005149	KCNIP4	40156628	40399467	6
ENSOARG00000017521	HBEGF	48917000	48951593	5	ENSOARG00000012378	SLC4A4	86143053	86457688	6	ENSOARG00000006066	JAKMIP1	102735767	102808907	6
ENSOARG00000018880	MAN2A1	105984860	106179347	5	ENSOARG00000014034	DNAJB14	24829721	24873057	6	ENSOARG00000009491	DTHD1	55697169	55784117	6
ENSOARG00000018924	TMEM232	106642215	106911809	5	ENSOARG00000014880	MTHFD2L	88783856	88905590	6	ENSOARG00000010289	ARHGEF38	19650992	19793944	6
ENSOARG00000025296		17857816	17992134	5	ENSOARG00000003285	EML5	97190353	97312451	7	ENSOARG00000010430	PPA2	19864955	19945670	6
ENSOARG00000000537	ANK2	12362227	12719843	6	ENSOARG00000018551		15384728	15392155	7	ENSOARG00000010632	TET2	20055989	20147275	6
ENSOARG00000003887	MAPK10	100456410	100650949	6	ENSOARG00000020735	ZNF609	42475435	42642760	7	ENSOARG00000014880	MTHFD2L	88783856	88905590	6
ENSOARG00000005149	KCNIP4	40156628	40399467	6	ENSOARG00000020996	ATP8B4	57145756	57436036	7	ENSOARG00000003285	EML5	97190353	97312451	7
ENSOARG00000007185	STK32B	103332684	103713145	6	ENSOARG00000021121	RTN1	68766907	68890740	7	ENSOARG00000020893	PRTG	51635068	51773448	7
ENSOARG00000010430	PPA2	19864955	19945670	6	ENSOARG00000021191	SLC8A3	78697982	78837399	7	ENSOARG00000003278	CCDC170	75143193	75234117	8
ENSOARG00000010632	TET2	20055989	20147275	6	ENSOARG00000026710		57124667	57275633	7	ENSOARG00000004895	RPS6KA2	88034242	88188079	8
ENSOARG00000018214	CWH43	67594632	67647488	6	ENSOARG00000001332	VTA1	66565201	66637042	8	ENSOARG00000009159		21064326	21097808	8
ENSOARG00000003122	SPATA7	96996666	97038856	7	ENSOARG00000004895	RPS6KA2	88034242	88188079	8	ENSOARG00000012911	RNGTT	48260082	48482195	8
ENSOARG00000003198		97148925	97186147	7	ENSOARG00000006899	PHIP	5690526	5800596	8	ENSOARG00000027019		44471402	44496409	8
ENSOARG00000003343	TTC8	97364708	97423931	7	ENSOARG00000009159		21064326	21097808	8	ENSOARG00000027025		48627836	48706773	8
ENSOARG00000016888	IQGAP2	7424541	7731473	7	ENSOARG00000011435	HACE1	32254404	32381662	8	ENSOARG00000009003	TRIQK	83920585	84012541	9

ENSOARG00000020851		48514282	48670343	7	ENSOARG00000012246		44791678	45077954	8	ENSOARG00000009412	FER1L6	28725879	28908736	9
ENSOARG00000020856	LIPC	48680866	48853721	7	ENSOARG00000012911	RNGTT	48260082	48482195	8	ENSOARG00000009876	ANXA13	29008574	29052918	9
ENSOARG00000021121	RTN1	68766907	68890740	7	ENSOARG00000001692	RGS22	76870092	77006581	9	ENSOARG00000010674	EIF3H	60424730	60523273	9
ENSOARG00000021126	PPM1A	69387661	69436582	7	ENSOARG00000009003	TRIQK	83920585	84012541	9	ENSOARG00000011267	CSMD3	63608850	65007715	9
ENSOARG00000002348	STXBP5	71462635	71627320	8	ENSOARG00000009876	ANXA13	29008574	29052918	9	ENSOARG00000000718	TGDS	69480374	69590150	10
ENSOARG00000002626	ZC3H12D	73543092	73565983	8	ENSOARG00000010674	EIF3H	60424730	60523273	9	ENSOARG00000006244	TDRD3	1835291	2065511	10
ENSOARG00000004773	AGPAT4	83449093	83583173	8	ENSOARG00000000718	TGDS	69480374	69590150	10	ENSOARG00000008433	TMCO3	86021273	86037791	10
ENSOARG00000004788		83818709	84599387	8	ENSOARG00000006515	COL4A2	84344246	84506524	10	ENSOARG00000002872	MBTD1	35042066	35112724	11
ENSOARG00000007878	NCOA7	12446609	12579375	8	ENSOARG00000008433	TMCO3	86021273	86037791	10	ENSOARG00000007848		7641415	8038744	11
ENSOARG00000027033		55303220	55340228	8	ENSOARG00000007848		7641415	8038744	11	ENSOARG00000017855	CHD3	27132435	27152030	11
ENSOARG00000004022	TRAPPC9	16049980	16369216	9	ENSOARG00000018887	ANKFY1	24333248	24399136	11	ENSOARG00000002070	RGL1	62892208	63060293	12
ENSOARG00000008448	CAB39L	19236825	19286656	10	ENSOARG00000026389		54096210	54271589	11	ENSOARG00000010714	USH2A	16999001	17981947	12
ENSOARG00000009964	TRPC4	24289442	24435384	10	ENSOARG00000026390		54225864	54263405	11	ENSOARG00000012654	RAB3GAP2	21760318	21865369	12
ENSOARG00000005519	PIPOX	20029543	20041627	11	ENSOARG00000002070	RGL1	62892208	63060293	12	ENSOARG00000015833	TP73	47455866	47502821	12
ENSOARG00000010799	FBXL20	39315577	39409307	11	ENSOARG00000002600	C1orf21	63602428	63754041	12	ENSOARG00000018748	MORN1	48507947	48556384	12
ENSOARG00000011855		45762020	45802167	11	ENSOARG00000009688		42897047	43151892	12	ENSOARG00000025437		23567301	23687517	12
ENSOARG00000012131	IKZF3	39697587	39784473	11	ENSOARG00000010714	USH2A	16999001	17981947	12	ENSOARG00000025438		23570015	23657488	12
ENSOARG00000015860		18748955	18836988	11	ENSOARG00000012654	RAB3GAP2	21760318	21865369	12	ENSOARG00000010095	PIGU	63488367	63582164	13
ENSOARG00000016763		23686551	23829312	11	ENSOARG00000012918	TRAF3IP3	71661157	71680824	12	ENSOARG00000011555		53619140	53734463	13
ENSOARG00000026373		40159758	40312440	11	ENSOARG00000018748	MORN1	48507947	48556384	12	ENSOARG00000018399	TTI1	66454593	66484327	13
ENSOARG00000013714	CD34	73589869	73605431	12	ENSOARG00000002785	KIAA1217	24120977	24476577	13	ENSOARG00000018960	RALGAPB	66983273	67058489	13
ENSOARG00000013891		73700588	73737242	12	ENSOARG00000006091		73855033	73868280	13	ENSOARG00000011682		54063550	54086186	14
ENSOARG00000002139	TTLL9	60717466	60772689	13	ENSOARG00000011461	SEL1L2	6917382	7009037	13	ENSOARG00000017939	FTO	21524991	21953995	14
ENSOARG00000002481	XKR7	60797386	60822789	13	ENSOARG00000015405	CAMK1D	16052740	16366434	13	ENSOARG00000005911	INSC	36936849	37000817	15
ENSOARG00000002820	HCK	60874932	60921921	13	ENSOARG00000017498	PCK1	57900057	57905307	13	ENSOARG00000008069	TEAD1	39369981	39631064	15
ENSOARG00000008288	NECAB3	62540336	62561533	13	ENSOARG00000018002	SPO11	58100842	58115189	13	ENSOARG00000011592	GLYATL2	79280017	79288198	15
ENSOARG00000008520		62551469	62552383	13	ENSOARG00000018399	TTI1	66454593	66484327	13	ENSOARG00000015999	IMMP1L	60430839	60496840	15
ENSOARG00000015308	CNBD2	64673075	64723487	13	ENSOARG00000007080		2787215	3071357	14	ENSOARG00000018423	UBASH3B	32853387	33003019	15
ENSOARG00000016562	GNAS	56774752	56830745	13	ENSOARG00000011682		54063550	54086186	14	ENSOARG00000006371	ADAMTS6	14045920	14324177	16
ENSOARG00000018635	DZANK1	37599912	37653541	13	ENSOARG00000017939	FTO	21524991	21953995	14	ENSOARG00000010187	WDR70	36614984	36892613	16
ENSOARG00000018960	RALGAPB	66983273	67058489	13	ENSOARG00000005819	SOX6	35537489	36100232	15	ENSOARG00000014702	ADCY2	65280195	65721184	16
ENSOARG00000026245		56795870	56916181	13	ENSOARG00000005911	INSC	36936849	37000817	15	ENSOARG00000017566		71881383	72067452	17
ENSOARG00000026248		64684046	64849522	13	ENSOARG00000007202	SPON1	38044689	38354554	15	ENSOARG00000004861	STXBP6	33919740	34146066	18
ENSOARG00000008717	XRCC1	50782808	50805182	14	ENSOARG00000011592	GLYATL2	79280017	79288198	15	ENSOARG00000006557	AKAP6	41629326	42119054	18

ENSOARG00000011355	BICRA	53602524	53676728	14	ENSOARG00000015999	IMMP1L	60430839	60496840	15	ENSOARG00000005786		48617258	48622991	19
ENSOARG00000026909		23102469	23275357	14	ENSOARG00000017917	GRIK4	30791427	31136076	15	ENSOARG00000007668	GRM7	18544027	19149524	19
ENSOARG00000026929		50701621	50891922	14	ENSOARG00000006371	ADAMTS6	14045920	14324177	16	ENSOARG00000014531	APPL1	43854675	43876438	19
ENSOARG00000010872	OR9I1	78493970	78662782	15	ENSOARG00000014702	ADCY2	65280195	65721184	16	ENSOARG00000016371	ARPP21	9569024	9683317	19
ENSOARG00000011446		79226138	79261515	15	ENSOARG00000001443	FSTL5	35976685	36901504	17	ENSOARG00000003926	CARMIL1	31188499	31494237	20
ENSOARG00000013240	ACER3	54380118	54544096	15	ENSOARG00000004146	FBXW7	4965419	5185723	17	ENSOARG00000005603	KHDRBS2	204311	1038981	20
ENSOARG00000016714	CCDC73	61520959	61671777	15	ENSOARG00000018865	KIAA1671	64862406	64986780	17	ENSOARG00000005654	PRIM2	2673361	2977511	20
ENSOARG00000019097	IFTAP	65231458	65301531	15	ENSOARG00000019041	GRK3	65098471	65205105	17	ENSOARG00000010300	SUPT3H	18310799	18706618	20
ENSOARG00000008401	PARP8	28331163	28521803	16	ENSOARG00000001046	CHRNA5	29994614	30020726	18	ENSOARG00000005183	DLG2	10779194	11743008	21
ENSOARG00000001443	FSTL5	35976685	36901504	17	ENSOARG00000004861	STXBP6	33919740	34146066	18	ENSOARG00000007919		23117728	23624748	21
ENSOARG00000011798	INPP4B	15088639	15543935	17	ENSOARG00000026453		48601602	48649268	18	ENSOARG00000002088	GRK5	38323057	38552331	22
ENSOARG00000017120	TMEM132B	49885384	50186678	17	ENSOARG00000007668	GRM7	18544027	19149524	19	ENSOARG00000005288	KIAA1328	20111910	20525120	23
ENSOARG00000009990		6726371	6750105	18	ENSOARG00000009293	CNTN3	27107120	27401680	19	ENSOARG00000004697	IQCE	40511246	40551800	24
ENSOARG00000000458	SFMBT1	47861509	47970530	19	ENSOARG00000014531	APPL1	43854675	43876438	19	ENSOARG00000010673		28285509	28426315	24
ENSOARG00000007465	PLXNA1	60171949	60201198	19	ENSOARG00000016371	ARPP21	9569024	9683317	19	ENSOARG00000011276		32699946	32762145	24
ENSOARG00000008494		49530241	49701797	19	ENSOARG00000003926	CARMIL1	31188499	31494237	20	ENSOARG00000011868		33115721	33286879	24
ENSOARG00000009813	SLC6A20	53424967	53471152	19	ENSOARG00000005654	PRIM2	2673361	2977511	20	ENSOARG00000015554		19838976	20025969	24
ENSOARG00000003613	UBR2	16358563	16459598	20	ENSOARG00000006418	HMGCLL1	4979745	5104686	20	ENSOARG00000015653		19931674	19979917	24
ENSOARG00000006418	HMGCLL1	4979745	5104686	20	ENSOARG00000009595	CDKAL1	35755504	36365789	20	ENSOARG00000025891		33169760	33290472	24
ENSOARG00000010300	SUPT3H	18310799	18706618	20	ENSOARG00000013835	PHACTR1	42250178	42485827	20	ENSOARG00000025902		40413481	40551726	24
ENSOARG00000002815	PACS1	43458201	43604405	21	ENSOARG00000007919		23117728	23624748	21	ENSOARG00000004227		15389097	15444034	25
ENSOARG00000003549	CNIH2	43635150	43640422	21	ENSOARG00000006805	CFAP43	23775242	23873559	22	ENSOARG00000003976		36739622	36795389	26
ENSOARG00000013080	DOCK1	45364051	45875506	22	ENSOARG00000005288	KIAA1328	20111910	20525120	23					
ENSOARG00000013216	PCDH15	4372641	5337209	22	ENSOARG00000005981	PIGN	60716730	60819102	23					
ENSOARG00000023496		45509628	45509742	22	ENSOARG00000004697	IQCE	40511246	40551800	24					
ENSOARG00000004215	NFATC1	748323	799990	23	ENSOARG00000010673		28285509	28426315	24					
ENSOARG00000006534	DSG4	26023321	26063151	23	ENSOARG00000011276		32699946	32762145	24					
ENSOARG00000026144		657340	807916	23	ENSOARG00000015554	POLR3E	19838976	20025969	24					
ENSOARG00000018283	CYP3A24	36495420	36645659	24	ENSOARG00000015653	EEF2K	19931674	19979917	24					
ENSOARG00000001975		33053238	33156974	26	ENSOARG00000025902		40413481	40551726	24					
ENSOARG00000004073	PSD3	37045901	37438136	26	ENSOARG00000004227	CCDC6	15389097	15444034	25					
ENSOARG00000006518	GPM6A	6098874	6137326	26	ENSOARG00000026405	RPS6KA1	18960060	19006408	25					

Supplementary Table S2. Top 1% genes as candidates for selective signals using xp-EHH test on 26 sheep autosomes for: a) IR and IN breeds, b) IR and AF breeds, c) IN and AF breeds.

a					b					c				
Gene.stable.ID	Gene.name	Gene.start	Gene.end	Chr.name	Gene.stable.ID	Gene.name	Gene.start	Gene.end	Chr.name	Gene.stable.ID	Gene.name	Gene.start	Gene.end	Chr.name
ENSOARG00000000049		217546133	217572906	1	ENSOARG00000006594	SLC4A10	146726620	146970354	2	ENSOARG00000014127	HLCS	267077034	267288456	1
ENSOARG00000000446	RAB3B	26126958	26230771	1	ENSOARG00000009240		158267452	158268171	2	ENSOARG00000010724	ACTL8	247660078	247663496	2
ENSOARG00000000458	BTF3L4	26248871	26277392	1	ENSOARG00000014059	BNC2	84290339	84688738	2	ENSOARG00000014195	TMEFF2	193228532	193510876	2
ENSOARG00000000541	HPS3	237051700	237092922	1	ENSOARG00000014183	CAVIN2	193088535	193102584	2	ENSOARG00000017452	MTX2	132661611	132737409	2
ENSOARG00000000558	SPSB4	245928191	245928902	1	ENSOARG00000014195	TMEFF2	193228532	193510876	2	ENSOARG00000020485	RHBDD1	228158186	228272756	2
ENSOARG00000000931	BRWD1	257369779	257471383	1	ENSOARG00000016675	FSIP2	122472091	122615086	2	ENSOARG00000021673		169649546	169649656	2
ENSOARG00000001137	AK5	53123782	53390133	1	ENSOARG00000016691		123263616	123264542	2	ENSOARG00000023420		169648692	169648802	2
ENSOARG00000001311	MORC3	266660290	266687301	1	ENSOARG00000017321	PRKRA	130564419	130579423	2	ENSOARG00000023757	U4	227996793	227996875	2
ENSOARG00000001975	CHAF1B	266698148	266724750	1	ENSOARG00000017335	OSBPL6	130606050	130693174	2	ENSOARG00000024578	U6	193361627	193361713	2
ENSOARG00000002120	CLDN14	266776355	266777907	1	ENSOARG00000017378		130949077	131370341	2	ENSOARG00000025024		169649900	169650014	2
ENSOARG00000002157	SIM2	267018135	267070595	1	ENSOARG00000017403	AGPS	131461739	131588288	2	ENSOARG00000014716	TRHDE	108235641	108685027	3
ENSOARG00000002217	HLCS	267077034	267288456	1	ENSOARG00000019628		131444363	131446354	2	ENSOARG00000015027	SYT1	114906496	115404501	3
ENSOARG00000002316	ERG	268709089	268782327	1	ENSOARG00000020485	RHBDD1	228158186	228272756	2	ENSOARG00000026012		108802884	108807540	3
ENSOARG00000002371	DPYD	73433343	74364967	1	ENSOARG00000020677		231918490	231924826	2	ENSOARG00000004800	EEPD1	60964689	61086480	4
ENSOARG00000002564	UGT1A1	7058779	7192169	1	ENSOARG00000020680	PSMD1	231939813	232016729	2	ENSOARG00000021411		81981748	81981878	4
ENSOARG00000002820	NGF	91785986	91843134	1	ENSOARG00000020689	HTR2B	231964788	231978154	2	ENSOARG00000025260		107947753	107958927	4
ENSOARG00000003100	KALRN	186773857	187401074	1	ENSOARG00000022968		130640657	130640789	2	ENSOARG00000014867	MSH3	78070508	78262288	5
ENSOARG00000003295	HIVEP3	15830095	15885701	1	ENSOARG00000023757	U4	227996793	227996875	2	ENSOARG00000016099	MEF2C	85606652	85706150	5
ENSOARG00000003385	ATP13A4	192150191	192276258	1	ENSOARG00000024578	U6	193361627	193361713	2	ENSOARG00000024846	MIR9-2	85551918	85552004	5
ENSOARG00000003685	TNIK	214087639	214487796	1	ENSOARG00000014716	TRHDE	108235641	108685027	3	ENSOARG00000011735	RUFY3	85751041	85822914	6
ENSOARG00000003798	S100A10	100863640	100867559	1	ENSOARG00000015027	SYT1	114906496	115404501	3	ENSOARG00000011975	MOB1B	85922030	85945597	6
ENSOARG00000003806	U6	129078476	129078582	1	ENSOARG00000015171	LIN7A	116695035	116841814	3	ENSOARG00000012101	DCK	85956079	85979964	6
ENSOARG00000003986		26283852	26283956	1	ENSOARG00000019622	NELL2	141536582	141928837	3	ENSOARG00000012378	SLC4A4	86143053	86457688	6
ENSOARG00000004112	U6	74164892	74164995	1	ENSOARG00000020137	COMMD1	45943376	46116851	3	ENSOARG00000000723		25137864	25138799	7
ENSOARG00000004131		214113907	214113981	1	ENSOARG00000004361	ANLN	60803769	60856028	4	ENSOARG00000002333	TMEM63C	85357829	85416748	7
ENSOARG00000004162		245911769	245917368	1	ENSOARG00000007333		20466277	20468265	4	ENSOARG00000002351	NGB	85426613	85430447	7
ENSOARG00000004334		266854180	266866713	1	ENSOARG00000018446	PTPRZ1	86318331	86517588	4	ENSOARG00000002382	POMT2	85438073	85471886	7
ENSOARG00000004595	SCN2A	143024729	143165286	2	ENSOARG00000025221		20439819	20599507	4	ENSOARG00000019856		25147983	25157854	7

ENSOARG00000004656	TANC1	149352369	149535503	2	ENSOARG00000025260		107947753	107958927	4	ENSOARG00000019861		25167333	25168919	7
ENSOARG00000004737	EPC2	159603473	159677031	2	ENSOARG00000010861	TACR3	21484074	21564709	6	ENSOARG00000021037	SQOR	61798658	61834331	7
ENSOARG00000004739		165154368	165495812	2	ENSOARG00000012378	SLC4A4	86143053	86457688	6	ENSOARG00000021039	BLOC1S6	61879421	61912418	7
ENSOARG00000004752	GFRA2	43555323	43658786	2	ENSOARG00000016735		116816991	116817494	6	ENSOARG00000021040	SLC30A4	61937980	61964478	7
ENSOARG00000004773	TBC1D2	49199068	49241910	2	ENSOARG00000016756		116844195	116845890	6	ENSOARG00000021078	GCH1	64275291	64331048	7
ENSOARG00000004895		201095999	201136885	2	ENSOARG00000026634		116805686	116807320	6	ENSOARG00000026714		61937799	62039205	7
ENSOARG00000004931	PARD3B	205957943	206670334	2	ENSOARG00000026635		116826888	116831975	6	ENSOARG00000004872	MPC1	87995562	87996759	8
ENSOARG00000004948	CAB39	231654998	231705560	2	ENSOARG00000026636		116830864	116831975	6	ENSOARG00000004895	RPS6KA2	88034242	88188079	8
ENSOARG00000004955	ITM2C	231740173	231752466	2	ENSOARG00000003122	SPATA7	96996666	97038856	7	ENSOARG00000012275		45307555	45359566	8
ENSOARG00000005112	SPATA3	231875615	231882882	2	ENSOARG00000003163	PTPN21	97057628	97126110	7	ENSOARG00000027012		33968090	33980976	8
ENSOARG00000005149		231918490	231924826	2	ENSOARG00000003198		97148925	97186147	7	ENSOARG00000027023		45307555	45506270	8
ENSOARG00000005226	PSMD1	231939813	232016729	2	ENSOARG00000003285	EML5	97190353	97312451	7	ENSOARG00000027024		45402145	45418001	8
ENSOARG00000005392	U6	201028585	201028685	2	ENSOARG00000021037	SQOR	61798658	61834331	7	ENSOARG00000027048		88033168	88034308	8
ENSOARG00000005519		15532406	15580456	2	ENSOARG00000021039	BLOC1S6	61879421	61912418	7	ENSOARG00000008657	ZNF572	28044608	28046904	9
ENSOARG00000005659		61818671	61896589	3	ENSOARG00000021040	SLC30A4	61937980	61964478	7	ENSOARG00000008715	MTSS1	28261745	28421317	9
ENSOARG00000005689	RANBP2	61917327	61963772	3	ENSOARG00000021043	SPATA5L1	62019680	62037987	7	ENSOARG00000008825	NDUFB9	28424418	28431883	9
ENSOARG00000006006	NOTO	95162096	95169384	3	ENSOARG00000021067	DDHD1	62633121	62701157	7	ENSOARG00000008916	TATDN1	28431947	28462592	9
ENSOARG00000006163	SMYD5	95171419	95181929	3	ENSOARG00000023637		97026422	97026507	7	ENSOARG00000009072	RNF139	28463518	28485500	9
ENSOARG00000006550	PRADC1	95184038	95187679	3	ENSOARG00000026714		61937799	62039205	7	ENSOARG00000011105		31854222	31870373	9
ENSOARG00000006564	CCT7	95188823	95204670	3	ENSOARG00000015035	PDE7B	61071786	61228713	8	ENSOARG00000014234		35135645	35277560	9
ENSOARG00000006579	ATP2B1	125851175	125982479	3	ENSOARG00000005919	NDUFAF6	81883016	81926044	9	ENSOARG00000015243	BPNT2	36711270	36729969	9
ENSOARG00000006783	KIF3C	33109792	33144386	3	ENSOARG00000006180	CCNE2	82068020	82079302	9	ENSOARG00000016046	RAB2A	39008886	39065425	9
ENSOARG00000006793		137514279	137544861	3	ENSOARG00000006512	INTS8	82077576	82125587	9	ENSOARG00000021727	U6	27276187	27276292	9
ENSOARG00000006876	NELL2	141536582	141928837	3	ENSOARG00000006836	DPY19L4	82149729	82199476	9	ENSOARG00000022168		36737787	36737865	9
ENSOARG00000007087	IAPP	193566307	193571714	3	ENSOARG00000008715	MTSS1	28261745	28421317	9	ENSOARG00000026514		27103578	27106919	9
ENSOARG00000007177	SLCO1A2	193610779	193663029	3	ENSOARG00000008825	NDUFB9	28424418	28431883	9	ENSOARG00000002377	UGGT2	72496105	72638712	10
ENSOARG00000007206		193676598	193750422	3	ENSOARG00000008916	TATDN1	28431947	28462592	9	ENSOARG00000012462		32430686	32431560	10
ENSOARG00000007299	U6	193729019	193729125	3	ENSOARG00000009072	RNF139	28463518	28485500	9	ENSOARG00000015029		42906101	43141913	10
ENSOARG00000007429	BBS9	63241800	63694790	4	ENSOARG00000011105		31854222	31870373	9	ENSOARG00000015071		43075598	43181328	10
ENSOARG00000007585	SLC35B4	97934780	97969641	4	ENSOARG00000011206	SPIDR	31974008	32251760	9	ENSOARG00000017100		39646385	39646768	10
ENSOARG00000007971		81543240	81854539	4	ENSOARG00000014234		35135645	35277560	9	ENSOARG00000017107		40068359	40068595	10
ENSOARG00000008018		54490731	54690430	4	ENSOARG00000014406		35794086	35795583	9	ENSOARG00000010714	USH2A	16999001	17981947	12

ENSOARG00000008076	OR2M4	39387987	39552662	5	ENSOARG00000015243	BPNT2	36711270	36729969	9	ENSOARG00000000049		19780214	20142442	13
ENSOARG00000009451	S1PR2	12433629	12435269	5	ENSOARG00000015579	LRP12	72670390	72737660	9	ENSOARG00000017847		19659509	19713910	13
ENSOARG00000009615	MRPL4	12455973	12464459	5	ENSOARG00000021727	U6	27276187	27276292	9	ENSOARG00000015418		44272723	44336115	15
ENSOARG00000009618	ICAM1	12500037	12509283	5	ENSOARG00000022168		36737787	36737865	9	ENSOARG00000015520		44463621	44464961	15
ENSOARG00000009910	MEF2C	85606652	85706150	5	ENSOARG00000022430	SNORA14B	81895388	81895522	9	ENSOARG00000015570	OVCH2	44494449	44517443	15
ENSOARG00000010632	PFDN1	48825562	48896702	5	ENSOARG00000026514		27103578	27106919	9	ENSOARG00000015682	CYB5R2	44541659	44549244	15
ENSOARG00000010642	U6	12448037	12448140	5	ENSOARG00000002377	UGGT2	72496105	72638712	10	ENSOARG00000015882	PPFIBP2	44557277	44676789	15
ENSOARG00000010729		12439622	12439686	5	ENSOARG00000006560	PCDH17	5437832	5545654	10	ENSOARG00000016441		29897938	29900669	15
ENSOARG00000010799		16405363	16601603	5	ENSOARG00000015029		42906101	43141913	10	ENSOARG00000017190		44365815	44366747	15
ENSOARG00000011100	KCNIP4	40156628	40399467	6	ENSOARG00000015071		43075598	43181328	10	ENSOARG00000017200		44447648	44448583	15
ENSOARG00000011404	TET2	20055989	20147275	6	ENSOARG00000015136	KLHL1	44398314	44721829	10	ENSOARG00000024850	U6	44524030	44524133	15
ENSOARG00000011462	GPR65	96636242	96637267	7	ENSOARG00000017100		39646385	39646768	10	ENSOARG00000004468	THOC5	68236030	68263906	17
ENSOARG00000011506		25055731	25112579	7	ENSOARG00000001311	NDEL1	27586115	27616932	11	ENSOARG00000004838	NIPSNAP1	68267050	68281030	17
ENSOARG00000011697		25087233	25088353	7	ENSOARG00000001617	MYH10	27625138	27747562	11	ENSOARG00000004984	NF2	68293690	68350562	17
ENSOARG00000011712		48514282	48670343	7	ENSOARG00000001933	CCDC42	27821246	27834755	11	ENSOARG00000005225		68313915	68314802	17
ENSOARG00000011809	SMOC1	78597451	78673983	7	ENSOARG00000004334		27553456	27553671	11	ENSOARG00000018082	SART3	64042329	64081333	17
ENSOARG00000011826	SLC8A3	78697982	78837399	7	ENSOARG00000009542	DCAKD	44146007	44170968	11	ENSOARG00000018146	FICD	64084833	64087190	17
ENSOARG00000012272	U6	78600847	78600956	7	ENSOARG00000009593		44173169	44203948	11	ENSOARG00000004861	STXBP6	33919740	34146066	18
ENSOARG00000012935	MAP3K4	83369733	83440796	8	ENSOARG00000023893		27736355	27736449	11	ENSOARG00000005909	AP4S1	40603299	40621837	18
ENSOARG00000013080	AGPAT4	83449093	83583173	8	ENSOARG00000010714	USH2A	16999001	17981947	12	ENSOARG00000006027	HECTD1	40626204	40697245	18
ENSOARG00000013382	RPS6KA2	88034242	88188079	8	ENSOARG00000011227		44885279	45057801	12	ENSOARG00000008679		68343351	68355744	18
ENSOARG00000013914	HS3ST5	23301492	23469323	8	ENSOARG00000012422	ESPN	45017490	45050484	12	ENSOARG00000008742		68378108	68383612	18
ENSOARG00000013952	LIN28B	32059283	32174641	8	ENSOARG00000012956	MARK1	22148091	22285882	12	ENSOARG00000024702		34383351	34383431	18
ENSOARG00000013992		33968090	33980976	8	ENSOARG00000002481	XKR7	60797386	60822789	13	ENSOARG00000026481		68373351	68376670	18
ENSOARG00000014030		88033168	88034308	8	ENSOARG00000002564		60835911	60855548	13	ENSOARG00000008743	CNTN4	23126508	23921247	19
ENSOARG00000014072	CPQ	79583307	80022406	9	ENSOARG00000017418		57854973	57864452	13	ENSOARG00000011479	CRTAC1	18239819	18367698	22
ENSOARG00000014127	NKAIN3	40545562	40801846	9	ENSOARG00000017498	PCK1	57900057	57905307	13	ENSOARG00000021318		18433127	18433187	22
ENSOARG00000014488		41066742	41066915	9	ENSOARG00000017818	CTCFL	57946183	57970701	13	ENSOARG00000000089	GTF3C1	25010620	25089541	24
ENSOARG00000015308	NDEL1	27586115	27616932	11	ENSOARG00000017959	RAE1	58073348	58092870	13	ENSOARG00000000347	TRRAP	37140796	37227404	24
ENSOARG00000015375	NEK8	19779100	19786873	11	ENSOARG00000018002	SPO11	58100842	58115189	13	ENSOARG00000000439	KIAA0556	25089816	25321497	24
ENSOARG00000015424	TRAF4	19788399	19794104	11	ENSOARG00000018039	BMP7	58173131	58255337	13	ENSOARG00000000845		25333567	25379060	24
ENSOARG00000015562	FAM222B	19799477	19808342	11	ENSOARG00000009501		8152352	8162610	14	ENSOARG00000001158	LAT	25866851	25870792	24
ENSOARG00000015602		27553456	27553671	11	ENSOARG00000000818	TRIM68	48828042	48836776	15	ENSOARG00000001555	NFATC2IP	25890362	25899904	24

ENSOARG00000015656	PIPOX	20029543	20041627	11	ENSOARG00000006555	OR51D1	48804671	48805642	15	ENSOARG00000001710	CD19	25905178	25915071	24
ENSOARG00000015673	MYO18A	20058835	20151305	11	ENSOARG00000001443	FSTL5	35976685	36901504	17	ENSOARG00000001825	RABEP2	25921006	25932126	24
ENSOARG00000015680	CRYBA1	20215842	20222612	11	ENSOARG00000004861	STXBP6	33919740	34146066	18	ENSOARG00000002132	ATP2A1	25931953	25950361	24
ENSOARG00000015828	TAOK1	20329392	20426865	11	ENSOARG00000004978	TRAF3	66251073	66299780	18	ENSOARG00000002503	ATXN2L	25982899	25992849	24
ENSOARG00000015942	FBXL20	39315577	39409307	11	ENSOARG00000006557	AKAP6	41629326	42119054	18	ENSOARG00000002826		25997816	25998752	24
ENSOARG00000016099	BRIP1	10781894	10963192	11	ENSOARG00000010549	LRIG1	34975963	35089024	19	ENSOARG00000002892		26017951	26038745	24
ENSOARG00000016110		38822779	38822875	11	ENSOARG00000010656	SLC25A26	35091509	35232652	19	ENSOARG00000002994		26052066	26063735	24
ENSOARG00000016442	5S_rRNA	19809497	19809594	11	ENSOARG00000003301	EXOC2	50814198	50938070	20	ENSOARG00000003124		26065424	26069206	24
ENSOARG00000016708	SNORA72	20171313	20171444	11	ENSOARG00000016571		44982565	45218014	20	ENSOARG00000003180	IL27	26070181	26075232	24
ENSOARG00000017054		20164604	20214384	11	ENSOARG00000018445	F13A1	47734507	47872375	20	ENSOARG00000003465	RADIL	39012202	39064415	24
ENSOARG00000017418		4582491	4601032	12	ENSOARG00000026890		50789331	50794993	20	ENSOARG00000003582	AP5Z1	39064376	39072830	24
ENSOARG00000017471		19780214	20142442	13	ENSOARG00000010436	MMS19	17890450	17925365	22	ENSOARG00000003652	FOXK1	39087521	39135075	24
ENSOARG00000017549		60835911	60855548	13	ENSOARG00000021318		18433127	18433187	22	ENSOARG00000003718	MAPK3	26187757	26198895	24
ENSOARG00000017573	HCK	60874932	60921921	13	ENSOARG00000000089	GTF3C1	25010620	25089541	24	ENSOARG00000003771	GDPD3	26195548	26200790	24
ENSOARG00000017701	C20orf194	51137982	51282854	13	ENSOARG00000000110	SMURF1	37110700	37133651	24	ENSOARG00000003856		39315644	39568156	24
ENSOARG00000018039	SLC4A11	51295540	51304552	13	ENSOARG00000000347	TRRAP	37140796	37227404	24	ENSOARG00000003882	YPEL3	26202421	26205022	24
ENSOARG00000018082	ITPA	51306908	51319485	13	ENSOARG00000000439	KIAA0556	25089816	25321497	24	ENSOARG00000004040	TBX6	26206479	26210145	24
ENSOARG00000018146	DDRGK1	51322212	51335874	13	ENSOARG00000000602	NPTX2	37374225	37384919	24	ENSOARG00000004100	PPP4C	26211200	26218665	24
ENSOARG00000018381	OXT	51437893	51438654	13	ENSOARG00000000845		25333567	25379060	24	ENSOARG00000004143		26218820	26227471	24
ENSOARG00000018463		51453752	51455892	13	ENSOARG00000001158	LAT	25866851	25870792	24	ENSOARG00000004277		39599497	39872553	24
ENSOARG00000018718		51127715	51128704	13	ENSOARG00000001555	NFATC2IP	25890362	25899904	24	ENSOARG00000004356	CARD11	40316370	40355402	24
ENSOARG00000019217	PTPRA	51465912	51530581	13	ENSOARG00000001710	CD19	25905178	25915071	24	ENSOARG00000004437	GNA12	40358096	40462023	24
ENSOARG00000019251	VPS16	51640703	51674356	13	ENSOARG00000001825	RABEP2	25921006	25932126	24	ENSOARG00000004504	AMZ1	40472597	40484725	24
ENSOARG00000019622	CNBD2	64673075	64723487	13	ENSOARG00000002132	ATP2A1	25931953	25950361	24	ENSOARG00000004594		40504639	40511497	24
ENSOARG00000019850		57854973	57864452	13	ENSOARG00000002503	ATXN2L	25982899	25992849	24	ENSOARG00000004697	IQCE	40511246	40551800	24
ENSOARG00000019853	BMP7	58173131	58255337	13	ENSOARG00000002826		25997816	25998752	24	ENSOARG00000004887	TTYH3	40571285	40593681	24
ENSOARG00000020138		48604903	48851165	13	ENSOARG00000002892		26017951	26038745	24	ENSOARG00000004992	LFNG	40603433	40611924	24
ENSOARG00000020197		48820759	48877389	13	ENSOARG00000002994		26052066	26063735	24	ENSOARG00000005030	KCTD13	26354516	26367538	24
ENSOARG00000020214		64684046	64849522	13	ENSOARG00000003124		26065424	26069206	24	ENSOARG00000005092	ASPHD1	26368344	26371923	24
ENSOARG00000020369		59732746	59746310	14	ENSOARG00000003180	IL27	26070181	26075232	24	ENSOARG00000005130	SEZ6L2	26373702	26393367	24
ENSOARG00000020378	NCR1	59754651	59759266	14	ENSOARG00000003465	RADIL	39012202	39064415	24	ENSOARG00000005487		26443971	26493101	24
ENSOARG00000020390		59783522	59939846	14	ENSOARG00000003582	AP5Z1	39064376	39072830	24	ENSOARG00000005716	QPRT	26506332	26507999	24
ENSOARG00000020420		59876096	59883028	14	ENSOARG00000003652	FOXK1	39087521	39135075	24	ENSOARG00000005724		26526804	26528279	24

ENSOARG00000020433		59902860	59913735	14	ENSOARG00000003718	MAPK3	26187757	26198895	24	ENSOARG00000005865	CD2BP2	26558468	26560111	24
ENSOARG00000020671		48886844	48889584	15	ENSOARG00000003771	GDPD3	26195548	26200790	24	ENSOARG00000005979	TBC1D10B	26563335	26573288	24
ENSOARG00000020673		48886985	48887446	15	ENSOARG00000003856		39315644	39568156	24	ENSOARG00000006071	MYLPF	26577862	26579570	24
ENSOARG00000020675		48921683	48922636	15	ENSOARG00000003882	YPEL3	26202421	26205022	24	ENSOARG00000006135	SEPTIN1	26580136	26583513	24
ENSOARG00000020677	C1QTNF5	29389458	29390834	15	ENSOARG00000004040	TBX6	26206479	26210145	24	ENSOARG00000006258		26603368	26610707	24
ENSOARG00000020680	MFRP	29390596	29396511	15	ENSOARG00000004100	PPP4C	26211200	26218665	24	ENSOARG00000006284	DCTPP1	26614394	26617494	24
ENSOARG00000020761	USP2	29405834	29421267	15	ENSOARG00000004143		26218820	26227471	24	ENSOARG00000006294		26629126	26630460	24
ENSOARG00000020851	RNF26	29385024	29386343	15	ENSOARG00000004356	CARD11	40316370	40355402	24	ENSOARG00000006423	ITGAL	26679858	26721693	24
ENSOARG00000021077	AK6	10361305	10375165	16	ENSOARG00000004437	GNA12	40358096	40462023	24	ENSOARG00000006854	PRR14	26793302	26798414	24
ENSOARG00000021190	CCDC125	10390597	10411746	16	ENSOARG00000004504	AMZ1	40472597	40484725	24	ENSOARG00000006922	FBRS	26801050	26811701	24
ENSOARG00000021191	ARL15	24832234	25070666	16	ENSOARG00000004594		40504639	40511497	24	ENSOARG00000007922	BCL7C	26980759	26985417	24
ENSOARG00000021335	SLC1A3	37483723	37563198	16	ENSOARG00000004697	IQCE	40511246	40551800	24	ENSOARG00000008040		26987973	26990899	24
ENSOARG00000021510	AGXT2	39050049	39092566	16	ENSOARG00000004887	TTYH3	40571285	40593681	24	ENSOARG00000008120	FBXL19	27052748	27071677	24
ENSOARG00000021555	DNAJC21	39118001	39143364	16	ENSOARG00000004992	LFNG	40603433	40611924	24	ENSOARG00000008151	ORAI3	27073875	27081653	24
ENSOARG00000021704	5S_rRNA	37411219	37411332	16	ENSOARG00000005030	KCTD13	26354516	26367538	24	ENSOARG00000008207		27082568	27104615	24
ENSOARG00000022105		4541467	4572685	17	ENSOARG00000005092	ASPHD1	26368344	26371923	24	ENSOARG00000008255	HSD3B7	27106043	27108526	24
ENSOARG00000022151	SART3	64042329	64081333	17	ENSOARG00000005130	SEZ6L2	26373702	26393367	24	ENSOARG00000008744	KAT8	27211624	27221382	24
ENSOARG00000022911	FICD	64084833	64087190	17	ENSOARG00000005487		26443971	26493101	24	ENSOARG00000008811		27222406	27226048	24
ENSOARG00000023060	BAZ1A	44016642	44094936	18	ENSOARG00000005716	QPRT	26506332	26507999	24	ENSOARG00000008863		27229167	27231806	24
ENSOARG00000023104		44166626	44194878	18	ENSOARG00000005724		26526804	26528279	24	ENSOARG00000008869		27259119	27265626	24
ENSOARG00000023268	FAM177A1	44252555	44262354	18	ENSOARG00000005865	CD2BP2	26558468	26560111	24	ENSOARG00000008908	PYCARD	27279014	27280168	24
ENSOARG00000023496	PPP2R3C	44263406	44291760	18	ENSOARG00000005979	TBC1D10B	26563335	26573288	24	ENSOARG00000008958	TRIM72	27290363	27297934	24
ENSOARG00000023679	PSMA6	44447953	44471341	18	ENSOARG00000006071	MYLPF	26577862	26579570	24	ENSOARG00000009000	ITGAM	27341076	27384550	24
ENSOARG00000024268	PDE8A	21759426	21904086	18	ENSOARG00000006135	SEPTIN1	26580136	26583513	24	ENSOARG00000009130	ITGAX	27400568	27419288	24
ENSOARG00000024270		44025256	44026309	18	ENSOARG00000006258		26603368	26610707	24	ENSOARG00000009250		27446741	27473467	24
ENSOARG00000024949	SFMBT1	47861509	47970530	19	ENSOARG00000006284	DCTPP1	26614394	26617494	24	ENSOARG00000009432	COX6A2	27474854	27475391	24
ENSOARG00000025234		47978524	48028706	19	ENSOARG00000006294		26629126	26630460	24	ENSOARG00000009484	ARMC5	27490512	27497969	24
ENSOARG00000025294	ERC2	44591299	45551630	19	ENSOARG00000006423	ITGAL	26679858	26721693	24	ENSOARG00000009817	SEPTIN14	27566765	27582185	24
ENSOARG00000025626	U5	45181051	45181168	19	ENSOARG00000006854	PRR14	26793302	26798414	24	ENSOARG00000009864		27613500	27618310	24
ENSOARG00000025645	FKBP5	9692040	9766541	20	ENSOARG00000006922	FBRS	26801050	26811701	24	ENSOARG00000010011		27708661	27717935	24
ENSOARG00000025743		48062157	48322346	20	ENSOARG00000007922	BCL7C	26980759	26985417	24	ENSOARG00000010109		27719967	27730172	24
ENSOARG00000026237		37107927	37116861	21	ENSOARG00000008040		26987973	26990899	24	ENSOARG00000010150	PHKG1	27731323	27736862	24
ENSOARG00000026238	PLCE1	14970716	15337763	22	ENSOARG00000008120	FBXL19	27052748	27071677	24	ENSOARG00000010228		27743597	27746528	24

ENSOARG00000026248	DOCK1	45364051	45875506	22	ENSOARG00000008151	ORAI3	27073875	27081653	24	ENSOARG00000010380	CRCP	27932583	27968175	24
ENSOARG00000026357	INSYN2A	45535724	45574150	22	ENSOARG00000008207		27082568	27104615	24	ENSOARG00000010453	TPST1	28032989	28099175	24
ENSOARG00000026451		45509628	45509742	22	ENSOARG00000008255	HSD3B7	27106043	27108526	24	ENSOARG00000010484		16387268	16391608	24
ENSOARG00000027012	USP31	20777411	20855050	24	ENSOARG00000008744	KAT8	27211624	27221382	24	ENSOARG00000010507	RABGEF1	28157577	28180855	24
ENSOARG00000027048		33053238	33156974	26	ENSOARG00000008811		27222406	27226048	24	ENSOARG00000010552	ARL6IP1	16399732	16404458	24
					ENSOARG00000008863		27229167	27231806	24	ENSOARG00000010673		28285509	28426315	24
					ENSOARG00000008869		27259119	27265626	24	ENSOARG00000010710	CALN1	28550611	29016799	24
					ENSOARG00000008908	PYCARD	27279014	27280168	24	ENSOARG00000010716		16421625	16421954	24
					ENSOARG00000008958	TRIM72	27290363	27297934	24	ENSOARG00000010731		29062550	29089745	24
					ENSOARG00000009000	ITGAM	27341076	27384550	24	ENSOARG00000010753		23768794	23769432	24
					ENSOARG00000009130	ITGAX	27400568	27419288	24	ENSOARG00000010767		26561711	26562259	24
					ENSOARG00000009250		27446741	27473467	24	ENSOARG00000010866		17182255	17182446	24
					ENSOARG00000009432	COX6A2	27474854	27475391	24	ENSOARG00000010928	SMG1	16411396	16483879	24
					ENSOARG00000009484	ARMC5	27490512	27497969	24	ENSOARG00000012249	VPS35L	17113331	17243756	24
					ENSOARG00000010011		27708661	27717935	24	ENSOARG00000013593		34135113	34165507	24
					ENSOARG00000010109		27719967	27730172	24	ENSOARG00000013650	MDH2	34164879	34177170	24
					ENSOARG00000010150	PHKG1	27731323	27736862	24	ENSOARG00000013722	SRRM3	34217112	34247460	24
					ENSOARG00000010228		27743597	27746528	24	ENSOARG00000013748	HSPB1	34256246	34257717	24
					ENSOARG00000010453	TPST1	28032989	28099175	24	ENSOARG00000013833	SSC4D	34325067	34335720	24
					ENSOARG00000010484		16387268	16391608	24	ENSOARG00000014682		19098694	19358510	24
					ENSOARG00000010507	RABGEF1	28157577	28180855	24	ENSOARG00000015168		35432755	35438155	24
					ENSOARG00000010552	ARL6IP1	16399732	16404458	24	ENSOARG00000017677	CACNG3	21924568	22022189	24
					ENSOARG00000010673		28285509	28426315	24	ENSOARG00000017950	TNRC6A	22384968	22479794	24
					ENSOARG00000010710	CALN1	28550611	29016799	24	ENSOARG00000018283	CYP3A24	36495420	36645659	24
					ENSOARG00000010732	ITPRIPL2	16817916	16819445	24	ENSOARG00000018306	ARHGAP17	22575927	22630268	24
					ENSOARG00000010743		23023376	23024338	24	ENSOARG00000018442	LCMT1	22670317	22755114	24
					ENSOARG00000010753		23768794	23769432	24	ENSOARG00000018457		36569069	36595967	24
					ENSOARG00000010767		26561711	26562259	24	ENSOARG00000018461		22672598	22676620	24
					ENSOARG00000010866		17182255	17182446	24	ENSOARG00000018497	TMEM225B	36704386	36711882	24
					ENSOARG00000010928	SMG1	16411396	16483879	24	ENSOARG00000018512		36718853	36726417	24
					ENSOARG00000012008	GDE1	17049328	17074699	24	ENSOARG00000018518	ZNF655	36733005	36743576	24
					ENSOARG00000012037		17063475	17064629	24	ENSOARG00000018521	ZNF789	36764572	36773601	24
					ENSOARG00000012082	CCP110	17081831	17098590	24	ENSOARG00000018538		36782276	36797011	24

ENSOARG00000012249	VPS35L	17113331	17243756	24	ENSOARG00000018676		36858253	36867852	24
ENSOARG00000013722	SRRM3	34217112	34247460	24	ENSOARG00000018686		23284024	23287342	24
ENSOARG00000013748	HSPB1	34256246	34257717	24	ENSOARG00000018696	PDAP1	36877950	36883201	24
ENSOARG00000014682		19098694	19358510	24	ENSOARG00000018709		24665014	24665858	24
ENSOARG00000015117		35409415	35412574	24	ENSOARG00000018731	ARPC1B	36885477	36891494	24
ENSOARG00000015168		35432755	35438155	24	ENSOARG00000018781		36903067	36926200	24
ENSOARG00000017677	CACNG3	21924568	22022189	24	ENSOARG00000018824	KPNA7	36986738	37026203	24
ENSOARG00000017741	RBBP6	22202339	22237519	24	ENSOARG00000018832	IL4R	24897923	24921149	24
ENSOARG00000017950	TNRC6A	22384968	22479794	24	ENSOARG00000018894	IL21R	24987772	25002160	24
ENSOARG00000018283	CYP3A24	36495420	36645659	24	ENSOARG00000022049		23643906	23643986	24
ENSOARG00000018306	ARHGAP17	22575927	22630268	24	ENSOARG00000022216	SNORD14	30871659	30871748	24
ENSOARG00000018442	LCMT1	22670317	22755114	24	ENSOARG00000022461		27711344	27711477	24
ENSOARG00000018457		36569069	36595967	24	ENSOARG00000022869	Metazoa_SRP	36737619	36737895	24
ENSOARG00000018461		22672598	22676620	24	ENSOARG00000023381		27715209	27715348	24
ENSOARG00000018497	TMEM225B	36704386	36711882	24	ENSOARG00000023493	U6	26483547	26483653	24
ENSOARG00000018512		36718853	36726417	24	ENSOARG00000024511		26985376	26985458	24
ENSOARG00000018518	ZNF655	36733005	36743576	24	ENSOARG00000024633	5S_rRNA	23323009	23323126	24
ENSOARG00000018521	ZNF789	36764572	36773601	24	ENSOARG00000025884		24661530	24663614	24
ENSOARG00000018538		36782276	36797011	24	ENSOARG00000025885		27237144	27238017	24
ENSOARG00000018676		36858253	36867852	24	ENSOARG00000025900		38964478	39079933	24
ENSOARG00000018686		23284024	23287342	24	ENSOARG00000025901		39157209	39160570	24
ENSOARG00000018696	PDAP1	36877950	36883201	24	ENSOARG00000025902		40413481	40551726	24
ENSOARG00000018709		24665014	24665858	24	ENSOARG00000025903		40462329	40463477	24
ENSOARG00000018731	ARPC1B	36885477	36891494	24	ENSOARG00000025906		40565957	40580901	24
ENSOARG00000018781		36903067	36926200	24	ENSOARG00000025907		40590851	40620365	24
ENSOARG00000018797	NSMCE1	24802986	24837628	24	ENSOARG00000001975		33053238	33156974	26
ENSOARG00000018824	KPNA7	36986738	37026203	24	ENSOARG00000002025		33169749	33171167	26
ENSOARG00000018832	IL4R	24897923	24921149	24	ENSOARG00000002101	ADAM2	33174931	33293614	26
ENSOARG00000018894	IL21R	24987772	25002160	24	ENSOARG00000006518	GPM6A	6098874	6137326	26
ENSOARG00000022461		27711344	27711477	24	ENSOARG00000014127				
ENSOARG00000022869	Metazoa_SRP	36737619	36737895	24	ENSOARG00000010724				
ENSOARG00000023381		27715209	27715348	24	ENSOARG00000014195				

ENSOARG00000023493	U6	26483547	26483653	24	ENSOARG00000017452
ENSOARG00000024511		26985376	26985458	24	ENSOARG00000020485
ENSOARG00000024633	5S_rRNA	23323009	23323126	24	ENSOARG00000021673
ENSOARG00000025882		22192111	22201388	24	ENSOARG00000023420
ENSOARG00000025884		24661530	24663614	24	ENSOARG00000023757
ENSOARG00000025885		27237144	27238017	24	ENSOARG00000024578
ENSOARG00000025900		38964478	39079933	24	ENSOARG00000025024
ENSOARG00000025901		39157209	39160570	24	ENSOARG00000014716
ENSOARG00000025902		40413481	40551726	24	ENSOARG00000015027
ENSOARG00000025903		40462329	40463477	24	ENSOARG00000026012
ENSOARG00000025906		40565957	40580901	24	ENSOARG00000004800
ENSOARG00000025907		40590851	40620365	24	

Supplementary Table S3. Top 1% genes as candidates for selective signals using Rsb test on 26 sheep autosomes for: a) IR and IN breeds, b) IR and AF breeds, c) IN and AF breeds.

a					b					c				
Gene.stable.ID	Gene.name	Gene.start	Gene.end	Chr.name	Gene.stable.ID	Gene.name	Gene.start	Gene.end	Chr.name	Gene.stable.ID	Gene.name	Gene.start	Gene.end	Chr.name
ENSOARG00000001069	GOLIM4	217358248	217440402	1	ENSOARG00000003449	KPNA7	231368088	231455602	1	ENSOARG00000003449	GPR149	231368088	231455602	1
ENSOARG00000006715	PXYLP1	245708917	245795592	1	ENSOARG00000003740	IL4R	234108818	234120665	1	ENSOARG00000003740	SUCNR1	234108818	234120665	1
ENSOARG00000013878	DOP1B	266507332	266631255	1	ENSOARG00000003815	IL21R	234266541	234289075	1	ENSOARG00000004273	CLRN1	235210034	235254936	1
ENSOARG00000014030	CLDN14	266776355	266777907	1	ENSOARG00000003857		234351940	234380044	1	ENSOARG00000004317	MINDY4B	235291097	235319185	1
ENSOARG00000014072	SIM2	267018135	267070595	1	ENSOARG00000004273	U6	235210034	235254936	1	ENSOARG00000004352	SIAH2	235410094	235429513	1
ENSOARG00000014127	HLCS	267077034	267288456	1	ENSOARG00000004317		235291097	235319185	1	ENSOARG00000004604	TSC22D2	235714439	235757736	1
ENSOARG00000014488	ERG	268709089	268782327	1	ENSOARG00000004352	Metazoa_SRP	235410094	235429513	1	ENSOARG00000024241	5S_rRNA	235700178	235700288	1
ENSOARG00000014986	CYYR1	128046415	128160518	1	ENSOARG00000004459		235554016	235579046	1	ENSOARG00000010057	LRP1B	167894700	168966473	2
ENSOARG00000017506	TLCD4	71439658	71503934	1	ENSOARG00000004511	U6	235602581	235641111	1	ENSOARG00000013044		66897804	66998532	2
ENSOARG00000017518	RWDD3	71552433	71562707	1	ENSOARG00000004604		235714439	235757736	1	ENSOARG00000014195	TMEFF2	193228532	193510876	2
ENSOARG00000017549	DPYD	73433343	74364967	1	ENSOARG00000004641	5S_rRNA	236245001	236250588	1	ENSOARG00000014874	SCARA5	101069669	101198536	2
ENSOARG00000019204		6977241	7027543	1	ENSOARG00000005319	U6	237392618	237431369	1	ENSOARG00000014887	NUGGC	101230656	101280194	2
ENSOARG00000019205		7030143	7053778	1	ENSOARG00000005357	KPNA7	237771275	237771944	1	ENSOARG00000017452	MTX2	132661611	132737409	2
ENSOARG00000019217	UGT1A1	7058779	7192169	1	ENSOARG00000005395	IL4R	237781410	237783997	1	ENSOARG00000017462	HOXD1	132820016	132821208	2
ENSOARG00000019229		7116948	7118037	1	ENSOARG00000015385	IL21R	237523905	237524984	1	ENSOARG00000017472	HOXD3	132836522	132855765	2
ENSOARG00000020205	CCDC14	186571189	186616335	1	ENSOARG00000024241		235700178	235700288	1	ENSOARG00000015027	SYT1	114906496	115404501	3
ENSOARG00000020214	KALRN	186773857	187401074	1	ENSOARG00000014195	U6	193228532	193510876	2	ENSOARG00000019182	ADCY6	137206277	137224517	3
ENSOARG00000020748	FNDC3B	213189221	213466453	1	ENSOARG00000014874		101069669	101198536	2	ENSOARG00000019185	TEX49	137227325	137254969	3
ENSOARG00000020756	PLD1	213745436	214029215	1	ENSOARG00000014887	Metazoa_SRP	101230656	101280194	2	ENSOARG00000024346		137221947	137222007	3
ENSOARG00000020761	TNIK	214087639	214487796	1	ENSOARG00000017403		131461739	131588288	2	ENSOARG00000003585	ELMO1	59996352	60466308	4
ENSOARG00000025645		266854180	266866713	1	ENSOARG00000017452	U6	132661611	132737409	2	ENSOARG00000003703	AOAH	60565422	60759733	4
ENSOARG00000002229	PTPRU	237110418	237179460	2	ENSOARG00000019628		131444363	131446354	2	ENSOARG00000004665	KIAA0895	60877099	60931558	4
ENSOARG00000009910		165154368	165495812	2	ENSOARG00000019843	5S_rRNA	130839077	130839523	2	ENSOARG00000024107	U6	60610415	60610521	4
ENSOARG00000010642	GFRA2	43555323	43658786	2	ENSOARG00000024578	U6	193361627	193361713	2	ENSOARG00000003387		35847680	35979412	5
ENSOARG00000011082	GABBR2	48929785	49175240	2	ENSOARG00000015027	KPNA7	114906496	115404501	3	ENSOARG00000024846	MIR9-2	85551918	85552004	5
ENSOARG00000014059	BNC2	84290339	84688738	2	ENSOARG00000003703	IL4R	60565422	60759733	4	ENSOARG00000011975	MOB1B	85922030	85945597	6
ENSOARG00000014195	TMEFF2	193228532	193510876	2	ENSOARG00000024107	IL21R	60610415	60610521	4	ENSOARG00000012101	DCK	85956079	85979964	6
ENSOARG00000014501	CAAP1	94480790	94556081	2	ENSOARG00000012101		85956079	85979964	6	ENSOARG00000000713		25034849	25035784	7
ENSOARG00000015828		201095999	201136885	2	ENSOARG00000003122	U6	96996666	97038856	7	ENSOARG00000019846		25018237	25019457	7
ENSOARG00000017403	AGPS	131461739	131588288	2	ENSOARG00000003163		97057628	97126110	7	ENSOARG00000021039	BLOC1S6	61879421	61912418	7

ENSOARG00000017442	HNRNPA3	131764126	131772092	2	ENSOARG00000003198	Metazoa_SRP	97148925	97186147	7	ENSOARG00000008715	MTSS1	28261745	28421317	9
ENSOARG00000018381	PARD3B	205957943	206670334	2	ENSOARG00000003285		97190353	97312451	7	ENSOARG00000008825	NDUFB9	28424418	28431883	9
ENSOARG00000019628		131444363	131446354	2	ENSOARG00000021037	U6	61798658	61834331	7	ENSOARG00000008916	TATDN1	28431947	28462592	9
ENSOARG00000020671	CAB39	231654998	231705560	2	ENSOARG00000021039		61879421	61912418	7	ENSOARG00000009072	RNF139	28463518	28485500	9
ENSOARG00000020673	ITM2C	231740173	231752466	2	ENSOARG00000021040	5S_rRNA	61937980	61964478	7	ENSOARG00000009158	TMEM65	28577331	28591415	9
ENSOARG00000020675	SPATA3	231875615	231882882	2	ENSOARG00000026714	U6	61937799	62039205	7	ENSOARG00000011105		31854222	31870373	9
ENSOARG00000020677		231918490	231924826	2	ENSOARG00000008715	KPNA7	28261745	28421317	9	ENSOARG00000011169		31967164	31967945	9
ENSOARG00000021976		131772093	131772166	2	ENSOARG00000009072	IL4R	28463518	28485500	9	ENSOARG00000011206	SPIDR	31974008	32251760	9
ENSOARG00000022347	5S_rRNA	43652479	43652596	2	ENSOARG00000009158	IL21R	28577331	28591415	9	ENSOARG00000013119	SNTG1	33284698	33536484	9
ENSOARG00000023938		159692299	159692370	2	ENSOARG00000011105		31854222	31870373	9	ENSOARG00000013523		34288898	34292049	9
ENSOARG00000002277		1442982	2818577	3	ENSOARG00000011169	U6	31967164	31967945	9	ENSOARG00000013762	OPRK1	34559945	34587699	9
ENSOARG00000009844	RAD51AP1	209543164	209559806	3	ENSOARG00000011206		31974008	32251760	9	ENSOARG00000013787	ATP6V1H	34692808	34737156	9
ENSOARG00000009922		209562723	209603814	3	ENSOARG00000013119	Metazoa_SRP	33284698	33536484	9	ENSOARG00000013873	RGS20	34741563	34788644	9
ENSOARG00000013205	RFX8	100064145	100112455	3	ENSOARG00000013523		34288898	34292049	9	ENSOARG00000013906	TCEA1	34792204	34809864	9
ENSOARG00000015027	SYT1	114906496	115404501	3	ENSOARG00000013762	U6	34559945	34587699	9	ENSOARG00000013994	LYPLA1	34828218	34843128	9
ENSOARG00000015037		115491774	115599738	3	ENSOARG00000013787		34692808	34737156	9	ENSOARG00000014089		34852382	34856945	9
ENSOARG00000015054	PPP1R12A	115651729	115819054	3	ENSOARG00000013873	5S_rRNA	34741563	34788644	9	ENSOARG00000014234		35135645	35277560	9
ENSOARG00000015089	OTOGL	116081170	116251642	3	ENSOARG00000013906	U6	34792204	34809864	9	ENSOARG00000014406		35794086	35795583	9
ENSOARG00000015220	PPFIA2	117165907	117673453	3	ENSOARG00000013994	KPNA7	34828218	34843128	9	ENSOARG00000015464	CYP7A1	37492107	37500880	9
ENSOARG00000015266	METTL25	118244609	118352705	3	ENSOARG00000014089	IL4R	34852382	34856945	9	ENSOARG00000015568	SDCBP	37556752	37589709	9
ENSOARG00000015680	ATP2B1	125851175	125982479	3	ENSOARG00000014234	IL21R	35135645	35277560	9	ENSOARG00000015741	NSMAF	37590850	37637144	9
ENSOARG00000019622	NELL2	141536582	141928837	3	ENSOARG00000014406		35794086	35795583	9	ENSOARG00000016001	CA8	38775707	38843863	9
ENSOARG00000020369	IAPP	193566307	193571714	3	ENSOARG00000015346	U6	37432080	37451172	9	ENSOARG00000020172	NPBWR1	34449585	34450325	9
ENSOARG00000020420		193676598	193750422	3	ENSOARG00000015464		37492107	37500880	9	ENSOARG00000020188		37270733	37271020	9
ENSOARG00000022423	U6	112901292	112901402	3	ENSOARG00000015568	Metazoa_SRP	37556752	37589709	9	ENSOARG00000021727	U6	27276187	27276292	9
ENSOARG00000023666		115367304	115367384	3	ENSOARG00000015741		37590850	37637144	9	ENSOARG00000024287	U6	37370449	37370555	9
ENSOARG00000024268	U6	193729019	193729125	3	ENSOARG00000020172	U6	34449585	34450325	9	ENSOARG00000026519		33752330	33767686	9
ENSOARG00000025922		40336878	40475522	3	ENSOARG00000020188		37270733	37271020	9	ENSOARG00000026523		38811461	38972090	9
ENSOARG00000025940		2018023	2203956	3	ENSOARG00000021727	5S_rRNA	27276187	27276292	9	ENSOARG00000005401		79668729	79786381	10
ENSOARG00000002931	DOCK4	56152727	56624017	4	ENSOARG00000024287	U6	37370449	37370555	9	ENSOARG00000012459		32337738	32342084	10
ENSOARG00000006163	BBS9	63241800	63694790	4	ENSOARG00000005404	KPNA7	80812888	81064030	10	ENSOARG00000015029		42906101	43141913	10
ENSOARG00000007341		20485907	20503267	4	ENSOARG00000015029	IL4R	42906101	43141913	10	ENSOARG00000015071		43075598	43181328	10
ENSOARG00000008109		20507640	20508110	4	ENSOARG00000015071	IL21R	43075598	43181328	10	ENSOARG00000015136	KLHL1	44398314	44721829	10

ENSOARG00000009641	HDAC9	27257256	27548397	4	ENSOARG00000015098		44186251	44188129	10	ENSOARG00000017100		39646385	39646768	10
ENSOARG00000016894	SEMA3C	39952749	40157229	4	ENSOARG00000015136	U6	44398314	44721829	10	ENSOARG00000017107		40068359	40068595	10
ENSOARG00000017020		40240542	40276695	4	ENSOARG00000017100		39646385	39646768	10	ENSOARG00000022137		45178360	45178470	10
ENSOARG00000022219	7SK	43585193	43585497	4	ENSOARG00000026309	Metazoa_SRP	42878720	42976582	10	ENSOARG00000024940		45300770	45300914	10
ENSOARG00000024498		43608738	43608881	4	ENSOARG00000026330		80937982	80952858	10	ENSOARG00000026309		42878720	42976582	10
ENSOARG00000024559	U1	27510116	27510282	4	ENSOARG00000001311	U6	27586115	27616932	11	ENSOARG00000007848		7641415	8038744	11
ENSOARG00000025221		20439819	20599507	4	ENSOARG00000004334		27553456	27553671	11	ENSOARG00000010714	USH2A	16999001	17981947	12
ENSOARG00000025231		40164548	40177100	4	ENSOARG00000007848	5S_rRNA	7641415	8038744	11	ENSOARG00000011335	ESRRG	18028459	18270543	12
ENSOARG00000014867	MSH3	78070508	78262288	5	ENSOARG00000010120	U6	13398010	13706664	12	ENSOARG00000011440	GPATCH2	19041488	19239239	12
ENSOARG00000016317		47131601	47133514	5	ENSOARG00000010714	KPNA7	16999001	17981947	12	ENSOARG00000012956	MARK1	22148091	22285882	12
ENSOARG00000016394	KDM3B	47153096	47208649	5	ENSOARG00000011335	IL4R	18028459	18270543	12	ENSOARG00000024700	5S_rRNA	11765649	11765767	12
ENSOARG00000016711	FAM172A	90442594	90837397	5	ENSOARG00000011440	IL21R	19041488	19239239	12	ENSOARG00000025432		15688758	15744623	12
ENSOARG00000017471	PFDN1	48825562	48896702	5	ENSOARG00000011583		19934865	20025740	12	ENSOARG00000005021	C11orf49	75082024	75292560	15
ENSOARG00000024846	MIR9-2	85551918	85552004	5	ENSOARG00000012333	U6	21715224	21761741	12	ENSOARG00000022253	U6	75207209	75207281	15
ENSOARG00000010430	PPA2	19864955	19945670	6	ENSOARG00000012654		21760318	21865369	12	ENSOARG00000013279	TRIO	58524569	58764835	16
ENSOARG00000014775	CXCL1	88610487	88611559	6	ENSOARG00000012956	Metazoa_SRP	22148091	22285882	12	ENSOARG00000026449		42640907	42757716	18
ENSOARG00000016907	UNC5C	29146028	29348864	6	ENSOARG00000021397		13457544	13457637	12	ENSOARG00000018783	RPP40	48496563	48508914	20
ENSOARG00000017161	BMPR1B	29361947	29448079	6	ENSOARG00000022476	U6	21735312	21735408	12	ENSOARG00000020118		48472029	48472196	20
ENSOARG00000025038	U6	19846738	19846844	6	ENSOARG00000024577		21735009	21735118	12	ENSOARG00000006933	RSF1	17628601	17727239	21
ENSOARG00000003163	PTPN21	97057628	97126110	7	ENSOARG00000024981	5S_rRNA	19968594	19968693	12	ENSOARG00000007125		17740943	17758684	21
ENSOARG00000003363		97934898	98128215	7	ENSOARG00000025432	U6	15688758	15744623	12	ENSOARG00000007178	AQP11	17772141	17783694	21
ENSOARG00000004872	MPC1	87995562	87996759	8	ENSOARG00000017959	KPNA7	58073348	58092870	13	ENSOARG00000002358		22592000	22601336	22
ENSOARG00000027012		33968090	33980976	8	ENSOARG00000018002	IL4R	58100842	58115189	13	ENSOARG00000002541	AS3MT	22608612	22624523	22
ENSOARG00000004737	CPQ	79583307	80022406	9	ENSOARG00000018039	IL21R	58173131	58255337	13	ENSOARG00000011479	CRTAC1	18239819	18367698	22
ENSOARG00000016708	NKAIN3	40545562	40801846	9	ENSOARG00000013279		58524569	58764835	16	ENSOARG00000021318		18433127	18433187	22
ENSOARG00000023353		38500803	38500929	9	ENSOARG00000013859	U6	61653247	62142024	16	ENSOARG00000000089	GTF3C1	25010620	25089541	24
ENSOARG00000024940		45300770	45300914	10	ENSOARG00000026888		46594647	46727238	20	ENSOARG00000000110	SMURF1	37110700	37133651	24
ENSOARG00000003396	RAB34	19766081	19768816	11	ENSOARG00000002358	Metazoa_SRP	22592000	22601336	22	ENSOARG00000000347	TRRAP	37140796	37227404	24
ENSOARG00000003480	RPL23A	19771126	19774188	11	ENSOARG00000002541		22608612	22624523	22	ENSOARG00000000439	KIAA0556	25089816	25321497	24
ENSOARG00000003633	TLCD1	19774814	19776010	11	ENSOARG00000002693	U6	22638602	22803964	22	ENSOARG00000000602	NPTX2	37374225	37384919	24
ENSOARG00000003806	NEK8	19779100	19786873	11	ENSOARG00000010847		17972993	17975270	22	ENSOARG00000000845		25333567	25379060	24
ENSOARG00000004112	TRAF4	19788399	19794104	11	ENSOARG00000010881	5S_rRNA	17977310	17986653	22	ENSOARG00000001158	LAT	25866851	25870792	24
ENSOARG00000004162	FAM222B	19799477	19808342	11	ENSOARG00000010934	U6	17987695	18009405	22	ENSOARG00000001555	NFATC2IP	25890362	25899904	24

ENSOARG00000004706	FLOT2	19878859	19898145	11	ENSOARG00000010955	KPNA7	17992016	17992681	22	ENSOARG00000001710	CD19	25905178	25915071	24
ENSOARG00000004860	DHRS13	19895557	19899827	11	ENSOARG00000011479	IL4R	18239819	18367698	22	ENSOARG00000001825	RABEP2	25921006	25932126	24
ENSOARG00000005023	PHF12	19899684	19940512	11	ENSOARG00000021318	IL21R	18433127	18433187	22	ENSOARG00000002132	ATP2A1	25931953	25950361	24
ENSOARG00000006006	MYO18A	20058835	20151305	11	ENSOARG00000000089		25010620	25089541	24	ENSOARG00000002503	ATXN2L	25982899	25992849	24
ENSOARG00000006550	CRYBA1	20215842	20222612	11	ENSOARG00000000110	U6	37110700	37133651	24	ENSOARG00000002826		25997816	25998752	24
ENSOARG00000006793	TAOK1	20329392	20426865	11	ENSOARG00000000347		37140796	37227404	24	ENSOARG00000002892		26017951	26038745	24
ENSOARG00000009443	SRCIN1	38713890	38782542	11	ENSOARG00000000439	Metazoa_SRP	25089816	25321497	24	ENSOARG00000002994		26052066	26063735	24
ENSOARG00000011855		45762020	45802167	11	ENSOARG00000000602		37374225	37384919	24	ENSOARG00000003124		26065424	26069206	24
ENSOARG00000011927	EFCAB3	45819006	46286165	11	ENSOARG00000000845	U6	25333567	25379060	24	ENSOARG00000003180	IL27	26070181	26075232	24
ENSOARG00000012039		45819006	45874585	11	ENSOARG00000001555		25890362	25899904	24	ENSOARG00000003465	RADIL	39012202	39064415	24
ENSOARG00000012739	TANC2	46811589	47176828	11	ENSOARG00000001710	5S_rRNA	25905178	25915071	24	ENSOARG00000003582	AP5Z1	39064376	39072830	24
ENSOARG00000021706	SNORD42	19771567	19771634	11	ENSOARG00000001825	U6	25921006	25932126	24	ENSOARG00000003718	MAPK3	26187757	26198895	24
ENSOARG00000022066	SNORD4A	19772880	19772949	11	ENSOARG00000002132	KPNA7	25931953	25950361	24	ENSOARG00000003771	GDPD3	26195548	26200790	24
ENSOARG00000022595	U1	45765834	45765989	11	ENSOARG00000002503	IL4R	25982899	25992849	24	ENSOARG00000003856		39315644	39568156	24
ENSOARG00000023093	SNORD4B	19774020	19774090	11	ENSOARG00000002826	IL21R	25997816	25998752	24	ENSOARG00000003882	YPEL3	26202421	26205022	24
ENSOARG00000023268	5S_rRNA	19809497	19809594	11	ENSOARG00000002892		26017951	26038745	24	ENSOARG00000004040	TBX6	26206479	26210145	24
ENSOARG00000024270	SNORA72	20171313	20171444	11	ENSOARG00000002994	U6	26052066	26063735	24	ENSOARG00000004100	PPP4C	26211200	26218665	24
ENSOARG00000026357		20164604	20214384	11	ENSOARG00000003124		26065424	26069206	24	ENSOARG00000004143		26218820	26227471	24
ENSOARG00000005173		47936746	47937063	12	ENSOARG00000003180	Metazoa_SRP	26070181	26075232	24	ENSOARG00000004356	CARD11	40316370	40355402	24
ENSOARG00000007971		4582491	4601032	12	ENSOARG00000003465		39012202	39064415	24	ENSOARG00000004437	GNA12	40358096	40462023	24
ENSOARG00000009688		42897047	43151892	12	ENSOARG00000003582	U6	39064376	39072830	24	ENSOARG00000004504	AMZ1	40472597	40484725	24
ENSOARG00000009776		43048095	43049619	12	ENSOARG00000003718		26187757	26198895	24	ENSOARG00000004594		40504639	40511497	24
ENSOARG00000014362	COP1	55270060	55448384	12	ENSOARG00000003771	5S_rRNA	26195548	26200790	24	ENSOARG00000004697	IQCE	40511246	40551800	24
ENSOARG00000002139	TTLL9	60717466	60772689	13	ENSOARG00000003856	U6	39315644	39568156	24	ENSOARG00000005030	KCTD13	26354516	26367538	24
ENSOARG00000002416	PDRG1	60775487	60781584	13	ENSOARG00000003882	KPNA7	26202421	26205022	24	ENSOARG00000005071	GRIFIN	40661527	40662687	24
ENSOARG00000002627	ATRN	50871319	51003428	13	ENSOARG00000004040	IL4R	26206479	26210145	24	ENSOARG00000005092	ASPHD1	26368344	26371923	24
ENSOARG00000002824		70279996	70565519	13	ENSOARG00000004100	IL21R	26211200	26218665	24	ENSOARG00000005130	SEZ6L2	26373702	26393367	24
ENSOARG00000004955	PTPRA	51465912	51530581	13	ENSOARG00000004143		26218820	26227471	24	ENSOARG00000005487		26443971	26493101	24
ENSOARG00000005666	CPXM1	51707797	51714249	13	ENSOARG00000004356	U6	40316370	40355402	24	ENSOARG00000005716	QPRT	26506332	26507999	24
ENSOARG00000006341	IDH3B	51851446	51856015	13	ENSOARG00000004437		40358096	40462023	24	ENSOARG00000005724		26526804	26528279	24
ENSOARG00000006470	NOP56	51856440	51861821	13	ENSOARG00000004504	Metazoa_SRP	40472597	40484725	24	ENSOARG00000005865	CD2BP2	26558468	26560111	24
ENSOARG00000016821		36350793	36353304	13	ENSOARG00000004594		40504639	40511497	24	ENSOARG00000005979	TBC1D10B	26563335	26573288	24
ENSOARG00000018039	BMP7	58173131	58255337	13	ENSOARG00000004697	U6	40511246	40551800	24	ENSOARG00000006071	MYLPF	26577862	26579570	24

ENSOARG00000021353	SNORD57	51857762	51857832	13	ENSOARG00000005030		26354516	26367538	24	ENSOARG00000006135	SEPTIN1	26580136	26583513	24
ENSOARG00000021529		51859560	51859695	13	ENSOARG00000005092	5S_rRNA	26368344	26371923	24	ENSOARG00000006258		26603368	26610707	24
ENSOARG00000023567	SNORD86	51858587	51858672	13	ENSOARG00000005130	U6	26373702	26393367	24	ENSOARG00000006284	DCTPP1	26614394	26617494	24
ENSOARG00000023890	SNORD110	51860461	51860532	13	ENSOARG00000005487	KPNA7	26443971	26493101	24	ENSOARG00000006294		26629126	26630460	24
ENSOARG00000024011	SNORD56	51858091	51858161	13	ENSOARG00000005716	IL4R	26506332	26507999	24	ENSOARG00000006423	ITGAL	26679858	26721693	24
ENSOARG00000026237		48604903	48851165	13	ENSOARG00000005724	IL21R	26526804	26528279	24	ENSOARG00000006854	PRR14	26793302	26798414	24
ENSOARG00000002020	NLRP2	59657921	59687108	14	ENSOARG00000005865		26558468	26560111	24	ENSOARG00000006922	FBRS	26801050	26811701	24
ENSOARG00000002099		59701679	59708054	14	ENSOARG00000005979	U6	26563335	26573288	24	ENSOARG00000007922	BCL7C	26980759	26985417	24
ENSOARG00000002108		59711324	59721587	14	ENSOARG00000006071		26577862	26579570	24	ENSOARG00000008040		26987973	26990899	24
ENSOARG00000002120		59732746	59746310	14	ENSOARG00000006135	Metazoa_SRP	26580136	26583513	24	ENSOARG00000008120	FBXL19	27052748	27071677	24
ENSOARG00000002157	NCR1	59754651	59759266	14	ENSOARG00000006258		26603368	26610707	24	ENSOARG00000008151	ORAI3	27073875	27081653	24
ENSOARG00000009370	NECTIN2	51660177	51687767	14	ENSOARG00000006284	U6	26614394	26617494	24	ENSOARG00000008207		27082568	27104615	24
ENSOARG00000009405	TOMM40	51689549	51700871	14	ENSOARG00000006294		26629126	26630460	24	ENSOARG00000008255	HSD3B7	27106043	27108526	24
ENSOARG00000009438	APOE	51709732	51711208	14	ENSOARG00000006423	5S_rRNA	26679858	26721693	24	ENSOARG00000008744	KAT8	27211624	27221382	24
ENSOARG00000003068		2812872	2851062	15	ENSOARG00000006854	U6	26793302	26798414	24	ENSOARG00000008811		27222406	27226048	24
ENSOARG00000006475		48654209	48655153	15	ENSOARG00000006922	KPNA7	26801050	26811701	24	ENSOARG00000008863		27229167	27231806	24
ENSOARG00000006490		48681015	48681971	15	ENSOARG00000007922	IL4R	26980759	26985417	24	ENSOARG00000008869		27259119	27265626	24
ENSOARG00000006579		48921683	48922636	15	ENSOARG00000008040	IL21R	26987973	26990899	24	ENSOARG00000008908	PYCARD	27279014	27280168	24
ENSOARG00000006597		48961624	48962577	15	ENSOARG00000008120		27052748	27071677	24	ENSOARG00000008958	TRIM72	27290363	27297934	24
ENSOARG00000015265	EIF3F	44084299	44091753	15	ENSOARG00000008151	U6	27073875	27081653	24	ENSOARG00000009000	ITGAM	27341076	27384550	24
ENSOARG00000015306		44116583	44132850	15	ENSOARG00000008207		27082568	27104615	24	ENSOARG00000009130	ITGAX	27400568	27419288	24
ENSOARG00000011449	PRLR	38969273	39028126	16	ENSOARG00000008255	Metazoa_SRP	27106043	27108526	24	ENSOARG00000009250		27446741	27473467	24
ENSOARG00000026968		40674484	40697370	16	ENSOARG00000008744		27211624	27221382	24	ENSOARG00000009432	COX6A2	27474854	27475391	24
ENSOARG00000004783	CMKLR1	64260293	64261387	17	ENSOARG00000008811	U6	27222406	27226048	24	ENSOARG00000009484	ARMC5	27490512	27497969	24
ENSOARG00000007059		44135465	44136040	18	ENSOARG00000008863		27229167	27231806	24	ENSOARG00000009529	TGFB1I1	27500166	27506161	24
ENSOARG00000007087		44166626	44194878	18	ENSOARG00000008869	5S_rRNA	27259119	27265626	24	ENSOARG00000009632	SLC5A2	27512274	27518884	24
ENSOARG00000007299	PPP2R3C	44263406	44291760	18	ENSOARG00000008908	U6	27279014	27280168	24	ENSOARG00000009760	RUSF1	27519186	27533843	24
ENSOARG00000007351		44292540	44425667	18	ENSOARG00000008958	KPNA7	27290363	27297934	24	ENSOARG00000009817	SEPTIN14	27566765	27582185	24
ENSOARG00000007502	NFKBIA	44533631	44538036	18	ENSOARG00000009000	IL4R	27341076	27384550	24	ENSOARG00000009864		27613500	27618310	24
ENSOARG00000010742	AGBL1	16183925	16783433	18	ENSOARG00000009130	IL21R	27400568	27419288	24	ENSOARG00000010011		27708661	27717935	24
ENSOARG00000015742	IL16	26027666	26139840	18	ENSOARG00000009250		27446741	27473467	24	ENSOARG00000010109		27719967	27730172	24
ENSOARG00000021308	U6	44547039	44547145	18	ENSOARG00000009432	U6	27474854	27475391	24	ENSOARG00000010150	PHKG1	27731323	27736862	24
ENSOARG00000024702		34383351	34383431	18	ENSOARG00000009484		27490512	27497969	24	ENSOARG00000010228		27743597	27746528	24

ENSOARG00000000458	SFMBT1	47861509	47970530	19	ENSOARG00000009529	Metazoa_SRP	27500166	27506161	24	ENSOARG00000010380	CRCP	27932583	27968175	24
ENSOARG00000001440	WNT7A	57988969	58046629	19	ENSOARG00000009632		27512274	27518884	24	ENSOARG00000010453	TPST1	28032989	28099175	24
ENSOARG00000012867	VGLL4	55462990	55496963	19	ENSOARG00000009760	U6	27519186	27533843	24	ENSOARG00000010507	RABGEF1	28157577	28180855	24
ENSOARG00000015375	ERC2	44591299	45551630	19	ENSOARG00000009817		27566765	27582185	24	ENSOARG00000010673		28285509	28426315	24
ENSOARG00000010300	SUPT3H	18310799	18706618	20	ENSOARG00000009864	5S_rRNA	27613500	27618310	24	ENSOARG00000010710	CALN1	28550611	29016799	24
ENSOARG00000010772	ILRUN	8762327	8806919	20	ENSOARG00000010011	U6	27708661	27717935	24	ENSOARG00000010716		16421625	16421954	24
ENSOARG00000011006		8986174	8993970	20	ENSOARG00000010109	KPNA7	27719967	27730172	24	ENSOARG00000010731		29062550	29089745	24
ENSOARG00000014123	PI16	10946568	10957116	20	ENSOARG00000010150	IL4R	27731323	27736862	24	ENSOARG00000010743		23023376	23024338	24
ENSOARG00000014201		10963849	10980577	20	ENSOARG00000010228	IL21R	27743597	27746528	24	ENSOARG00000010753		23768794	23769432	24
ENSOARG00000022664	5S_rRNA	18421702	18421834	20	ENSOARG00000010380		27932583	27968175	24	ENSOARG00000010757		29716072	29739127	24
ENSOARG00000025135		8644046	8644135	20	ENSOARG00000010453	U6	28032989	28099175	24	ENSOARG00000010767		26561711	26562259	24
ENSOARG00000003523	RAB1B	43626127	43634738	21	ENSOARG00000010507		28157577	28180855	24	ENSOARG00000010858		35467228	35467917	24
ENSOARG00000003549	CNIH2	43635150	43640422	21	ENSOARG00000010673	Metazoa_SRP	28285509	28426315	24	ENSOARG00000010866		17182255	17182446	24
ENSOARG00000003628	YIF1A	43641414	43645241	21	ENSOARG00000010710		28550611	29016799	24	ENSOARG00000010928	SMG1	16411396	16483879	24
ENSOARG00000003837	TMEM151A	43647082	43651665	21	ENSOARG00000010716	U6	16421625	16421954	24	ENSOARG00000011276		32699946	32762145	24
ENSOARG00000003798	PLCE1	14970716	15337763	22	ENSOARG00000010731		29062550	29089745	24	ENSOARG00000011464	SYT17	16671492	16750884	24
ENSOARG00000013080	DOCK1	45364051	45875506	22	ENSOARG00000010743	5S_rRNA	23023376	23024338	24	ENSOARG00000011839	TMC5	16976908	17040297	24
ENSOARG00000026741		14970716	15052778	22	ENSOARG00000010753	U6	23768794	23769432	24	ENSOARG00000012008	GDE1	17049328	17074699	24
ENSOARG00000004732	DCC	52377386	53214438	23	ENSOARG00000010757	KPNA7	29716072	29739127	24	ENSOARG00000012037		17063475	17064629	24
ENSOARG00000024531	U6	18034837	18034933	23	ENSOARG00000010767	IL4R	26561711	26562259	24	ENSOARG00000012082	CCP110	17081831	17098590	24
ENSOARG00000010332	SH2D4B	35539285	35620062	25	ENSOARG00000010816	IL21R	32523290	32571028	24	ENSOARG00000012249	VPS35L	17113331	17243756	24
ENSOARG00000001975		33053238	33156974	26	ENSOARG00000010858		35467228	35467917	24	ENSOARG00000012389	GPRC5B	17416805	17426893	24
ENSOARG00000006442	SBD2	5979383	5981052	26	ENSOARG00000010866	U6	17182255	17182446	24	ENSOARG00000013593		34135113	34165507	24
					ENSOARG00000010928		16411396	16483879	24	ENSOARG00000013650	MDH2	34164879	34177170	24
					ENSOARG00000011464	Metazoa_SRP	16671492	16750884	24	ENSOARG00000013722	SRRM3	34217112	34247460	24
					ENSOARG00000011839		16976908	17040297	24	ENSOARG00000013748	HSPB1	34256246	34257717	24
					ENSOARG00000012008	U6	17049328	17074699	24	ENSOARG00000014682		19098694	19358510	24
					ENSOARG00000012037		17063475	17064629	24	ENSOARG00000014711	COL26A1	35124398	35319320	24
					ENSOARG00000012249	5S_rRNA	17113331	17243756	24	ENSOARG00000014813	IFT22	35319511	35323830	24
					ENSOARG00000012389	U6	17416805	17426893	24	ENSOARG00000015117		35409415	35412574	24
					ENSOARG00000013592	KPNA7	18695046	18702323	24	ENSOARG00000015168		35432755	35438155	24
					ENSOARG00000013593	IL4R	34135113	34165507	24	ENSOARG00000015233	AP1S1	35472189	35475647	24
					ENSOARG00000013650	IL21R	34164879	34177170	24	ENSOARG00000015262	SERPINE1	35490295	35496243	24

ENSOARG00000013722		34217112	34247460	24	ENSOARG00000017218	PALB2	21271399	21297440	24
ENSOARG00000013748	U6	34256246	34257717	24	ENSOARG00000017281	DCTN5	21297798	21325007	24
ENSOARG00000013879		18706424	18938235	24	ENSOARG00000017631	PRKCB	21669605	21883171	24
ENSOARG00000014682	Metazoa_SRP	19098694	19358510	24	ENSOARG00000017677	CACNG3	21924568	22022189	24
ENSOARG00000014711		35124398	35319320	24	ENSOARG00000017741	RBBP6	22202339	22237519	24
ENSOARG00000014813	U6	35319511	35323830	24	ENSOARG00000017950	TNRC6A	22384968	22479794	24
ENSOARG00000015094		19686502	19711770	24	ENSOARG00000018306	ARHGAP17	22575927	22630268	24
ENSOARG00000015117	5S_rRNA	35409415	35412574	24	ENSOARG00000018442	LCMT1	22670317	22755114	24
ENSOARG00000015150	U6	19712463	19726247	24	ENSOARG00000018461		22672598	22676620	24
ENSOARG00000015168	KPNA7	35432755	35438155	24	ENSOARG00000018497	TMEM225B	36704386	36711882	24
ENSOARG00000015233	IL4R	35472189	35475647	24	ENSOARG00000018512		36718853	36726417	24
ENSOARG00000015262	IL21R	35490295	35496243	24	ENSOARG00000018518	ZNF655	36733005	36743576	24
ENSOARG00000015303		19817449	19870913	24	ENSOARG00000018676		36858253	36867852	24
ENSOARG00000015554	U6	19838976	20025969	24	ENSOARG00000018686		23284024	23287342	24
ENSOARG00000016442		20777411	20855050	24	ENSOARG00000018696	PDAP1	36877950	36883201	24
ENSOARG00000017218	Metazoa_SRP	21271399	21297440	24	ENSOARG00000018731	ARPC1B	36885477	36891494	24
ENSOARG00000017281		21297798	21325007	24	ENSOARG00000018797	NSMCE1	24802986	24837628	24
ENSOARG00000017631	U6	21669605	21883171	24	ENSOARG00000018824	KPNA7	36986738	37026203	24
ENSOARG00000017677		21924568	22022189	24	ENSOARG00000018832	IL4R	24897923	24921149	24
ENSOARG00000017741	5S_rRNA	22202339	22237519	24	ENSOARG00000018894	IL21R	24987772	25002160	24
ENSOARG00000018306	U6	22575927	22630268	24	ENSOARG00000022049		23643906	23643986	24
ENSOARG00000018442	KPNA7	22670317	22755114	24	ENSOARG00000022216	SNORD14	30871659	30871748	24
ENSOARG00000018461	IL4R	22672598	22676620	24	ENSOARG00000022333	U6	31075078	31075176	24
ENSOARG00000018497	IL21R	36704386	36711882	24	ENSOARG00000022461		27711344	27711477	24
ENSOARG00000018512		36718853	36726417	24	ENSOARG00000022869	Metazoa_SRP	36737619	36737895	24
ENSOARG00000018518	U6	36733005	36743576	24	ENSOARG00000023381		27715209	27715348	24
ENSOARG00000018676		36858253	36867852	24	ENSOARG00000023493	U6	26483547	26483653	24
ENSOARG00000018686	Metazoa_SRP	23284024	23287342	24	ENSOARG00000024511		26985376	26985458	24
ENSOARG00000018696		36877950	36883201	24	ENSOARG00000024633	5S_rRNA	23323009	23323126	24
ENSOARG00000018709	U6	24665014	24665858	24	ENSOARG00000025882		22192111	22201388	24
ENSOARG00000018731		36885477	36891494	24	ENSOARG00000025885		27237144	27238017	24
ENSOARG00000018797	5S_rRNA	24802986	24837628	24	ENSOARG00000025886		29309747	29489513	24
ENSOARG00000018824	U6	36986738	37026203	24	ENSOARG00000025887		29711793	29843090	24
ENSOARG00000018832	KPNA7	24897923	24921149	24	ENSOARG00000025889		31053456	31074351	24

ENSOARG00000018894	IL4R	24987772	25002160	24
ENSOARG00000022049	IL21R	23643906	23643986	24
ENSOARG00000022333		31075078	31075176	24
ENSOARG00000022461	U6	27711344	27711477	24
ENSOARG00000022869		36737619	36737895	24
ENSOARG00000023381	Metazoa_SRP	27715209	27715348	24
ENSOARG00000023493		26483547	26483653	24
ENSOARG00000024511	U6	26985376	26985458	24
ENSOARG00000024633		23323009	23323126	24
ENSOARG00000025076	5S_rRNA	19850199	19850306	24
ENSOARG00000025882	U6	22192111	22201388	24
ENSOARG00000025884	KPNA7	24661530	24663614	24
ENSOARG00000025885	IL4R	27237144	27238017	24
ENSOARG00000025886	IL21R	29309747	29489513	24
ENSOARG00000025887		29711793	29843090	24
ENSOARG00000025889	U6	31053456	31074351	24
ENSOARG00000025900		38964478	39079933	24
ENSOARG00000025901	Metazoa_SRP	39157209	39160570	24
ENSOARG00000025902		40413481	40551726	24
ENSOARG00000025903	U6	40462329	40463477	24

ENSOARG00000025900		38964478	39079933	24
ENSOARG00000025901		39157209	39160570	24
ENSOARG00000025902		40413481	40551726	24
ENSOARG00000025903		40462329	40463477	24

Supplementary Table S4. Top 1% genes as candidates for selective signals using FLK test on 26 sheep autosomes for: a) IR and IN breeds, b) IR and AF breeds, c) IN and AF breeds.

	a					b					c			
Gene.stable.ID	Gene.name	Gene.start	Gene.end	Chr.name	Gene.stable.ID	Gene.name	Gene.start	Gene.end	Chr.name	Gene.stable.ID	Gene.name	Gene.start	Gene.end	Chr.name
ENSOARG00000000342		101868308	101930299	1	ENSOARG00000000578	CDCA7	135465988	135478982	2	ENSOARG00000000578	CDCA7	135465988	135478982	2
ENSOARG00000000418	PHC3	215239784	215311459	1	ENSOARG00000001516	SERINC2	235085858	235104700	2	ENSOARG00000004384	RPS6KA1	239443298	239467559	2
ENSOARG00000000493		18080828	18124219	1	ENSOARG00000001603	FABP3	235135457	235145925	2	ENSOARG00000005756	BRINP1	4115880	4262567	2
ENSOARG00000000515	ST3GAL3	18120931	18347967	1	ENSOARG00000004384	RPS6KA1	239443298	239467559	2	ENSOARG00000005972	TNFSF15	8934595	8954674	2
ENSOARG00000001069	GOLIM4	217358248	217440402	1	ENSOARG00000006009	WHRN	9200709	9289247	2	ENSOARG00000006009	WHRN	9200709	9289247	2
ENSOARG00000005112	HPS3	237051700	237092922	1	ENSOARG00000006030	AKNA	9311734	9352831	2	ENSOARG00000006030	AKNA	9311734	9352831	2
ENSOARG00000009108	KANK4	37279376	37318481	1	ENSOARG00000006078	COL27A1	9374621	9516130	2	ENSOARG00000006078	COL27A1	9374621	9516130	2
ENSOARG00000009414	ATP2C1	256985186	257068039	1	ENSOARG00000006287	BSPRY	10282879	10310995	2	ENSOARG00000006287	BSPRY	10282879	10310995	2
ENSOARG00000009979		258241417	258581681	1	ENSOARG00000006300	WDR31	10332512	10346064	2	ENSOARG00000006300	WDR31	10332512	10346064	2
ENSOARG00000010761	TMPRSS3	260732616	260756933	1	ENSOARG00000007628		22623391	22636457	2	ENSOARG00000007440	GRIN3A	21252157	21459411	2
ENSOARG00000010806	UBASH3A	260769711	260809438	1	ENSOARG00000007715	KDM1A	242660932	242714290	2	ENSOARG00000008011	GALNT13	153919534	154286087	2
ENSOARG00000014697	GPR160	215320734	215321750	1	ENSOARG00000007814	SYK	25701083	25759453	2	ENSOARG00000008084	WNK2	28059615	28213657	2
ENSOARG00000016027	USP25	139564863	139699601	1	ENSOARG00000008084	WNK2	28059615	28213657	2	ENSOARG00000008659	NEB	156650358	156853665	2
ENSOARG00000019409	SLC6A17	86624899	86660561	1	ENSOARG00000008659	NEB	156650358	156853665	2	ENSOARG00000009898	GTDC1	164635825	164985339	2
ENSOARG00000020378	HIVEP3	15830095	15885701	1	ENSOARG00000009010	UBQLN1	35845653	35897664	2	ENSOARG00000010689	LCT	173878863	173926998	2
ENSOARG00000020761	TNIK	214087639	214487796	1	ENSOARG00000010057	LRP1B	167894700	168966473	2	ENSOARG00000010706	UBXN4	173931295	173953446	2
ENSOARG00000021056		100641877	100643585	1	ENSOARG00000010998	COL15A1	48487007	48588324	2	ENSOARG00000010998	COL15A1	48487007	48588324	2
ENSOARG00000021060	RORC	100648111	100672007	1	ENSOARG00000011055	PADI1	248168842	248189290	2	ENSOARG00000011055	PADI1	248168842	248189290	2
ENSOARG00000021065		100680890	100831184	1	ENSOARG00000011436	MGAT5	175253426	175595136	2	ENSOARG00000011436	MGAT5	175253426	175595136	2
ENSOARG00000021072	THEM4	100734906	100770288	1	ENSOARG00000011504	ZCCHC7	51076086	51317595	2	ENSOARG00000012588	CARNMT1	61274631	61304613	2
ENSOARG00000021983	U6	215303687	215303791	1	ENSOARG00000015710	HERC2	112478388	112720769	2	ENSOARG00000015637	LGSN	111995915	112021524	2
ENSOARG00000025511		17971643	18117954	1	ENSOARG00000019232	ERBB4	212081308	212484401	2	ENSOARG00000015655	OCA2	112126827	112463751	2
ENSOARG00000002545	EPB41	237305059	237487293	2	ENSOARG00000019758		215044960	215045145	2	ENSOARG00000015710	HERC2	112478388	112720769	2
ENSOARG00000003546	ENHO	36820768	36820998	2	ENSOARG00000020677		231918490	231924826	2	ENSOARG00000016969		129907617	130149171	2
ENSOARG00000008634		31239795	31476634	2	ENSOARG00000020868	AK2	233730933	233753041	2	ENSOARG00000019232	ERBB4	212081308	212484401	2
ENSOARG00000008659	NEB	156650358	156853665	2	ENSOARG00000024860	U2	151198443	151198552	2	ENSOARG00000019731		195160444	195160662	2
ENSOARG00000008930	RIF1	156861839	156919308	2	ENSOARG00000025847		215173693	215317344	2	ENSOARG00000019758		215044960	215045145	2
ENSOARG00000009251	DNAI1	36819526	36890350	2	ENSOARG00000002894	RTN4	68869891	68943954	3	ENSOARG00000020437	DOCK10	225910579	226106181	2

ENSOARG00000010960	ALG2	48313731	48317508	2	ENSOARG00000004226	NRXN1	73678833	73847033	3	ENSOARG00000024949	U6	201028585	201028685	2
ENSOARG00000011436	MGAT5	175253426	175595136	2	ENSOARG00000007419	ABL1	5731767	5765473	3	ENSOARG00000002894	RTN4	68869891	68943954	3
ENSOARG00000012027		52695717	52765806	2	ENSOARG00000008162	MAP4K3	84680681	84866029	3	ENSOARG00000004226	NRXN1	73678833	73847033	3
ENSOARG00000014195	TMEFF2	193228532	193510876	2	ENSOARG00000008165	HSD17B6	162593753	162604684	3	ENSOARG00000008165	HSD17B6	162593753	162604684	3
ENSOARG00000014477		91933439	91934623	2	ENSOARG00000009069	ATL2	85684525	85751972	3	ENSOARG00000009069	ATL2	85684525	85751972	3
ENSOARG00000015036	HMBOX1	102231442	102383638	2	ENSOARG00000010376		91050821	91234495	3	ENSOARG00000009740	EIF2AK2	86846562	86871162	3
ENSOARG00000016867	ITGA4	127163497	127254486	2	ENSOARG00000011771	TESPA1	164960462	164984444	3	ENSOARG00000009846	GPATCH11	86879693	86887627	3
ENSOARG00000017979	NBEAL1	204016478	204166655	2	ENSOARG00000012960	DCP1B	212314512	212358765	3	ENSOARG00000009946	HEATR5B	86893694	86992123	3
ENSOARG00000018104		204046005	204047405	2	ENSOARG00000013022	TMEM182	98927197	98974919	3	ENSOARG00000012360	ELK3	165522205	165579438	3
ENSOARG00000022859	U6	91862325	91862426	2	ENSOARG00000013033	MFSD9	98995237	99007482	3	ENSOARG00000012449	CDK17	165586544	165627612	3
ENSOARG00000024062	5S_rRNA	91845862	91845980	2	ENSOARG00000014173	ANO4	169622065	170061610	3	ENSOARG00000012960	DCP1B	212314512	212358765	3
ENSOARG00000024578	U6	193361627	193361713	2	ENSOARG00000014722	UTP20	170228637	170315908	3	ENSOARG00000014173	ANO4	169622065	170061610	3
ENSOARG00000025733		954711	1233648	2	ENSOARG00000015343	GNPTAB	170615182	170667987	3	ENSOARG00000014722	UTP20	170228637	170315908	3
ENSOARG00000025798		114046190	114223286	2	ENSOARG00000017302	HS1BP3	28343752	28391519	3	ENSOARG00000015054	PPP1R12A	115651729	115819054	3
ENSOARG00000025800		115725037	115837201	2	ENSOARG00000017314	GALNT6	134693949	134722893	3	ENSOARG00000016632		108814590	108815243	3
ENSOARG00000025826		170648415	170650818	2	ENSOARG00000017366	LDAH	28411571	28500725	3	ENSOARG00000017381	POU6F1	134849158	134869705	3
ENSOARG00000001759	FANCL	65770635	65858634	3	ENSOARG00000018354		32668067	32874601	3	ENSOARG00000018354		32668067	32874601	3
ENSOARG00000001844	VRK2	65858703	65955917	3	ENSOARG00000019050	NCF4	180081443	180103379	3	ENSOARG00000019050	NCF4	180081443	180103379	3
ENSOARG00000002277		1442982	2818577	3	ENSOARG00000019068		180109028	180109544	3	ENSOARG00000019068		180109028	180109544	3
ENSOARG00000002366	RXRA	1694451	1718246	3	ENSOARG00000019095	CSF2RB	180166727	180179868	3	ENSOARG00000019095	CSF2RB	180166727	180179868	3
ENSOARG00000011846	NTN4	165067364	165195408	3	ENSOARG00000019458	BABAM2	34767758	35168619	3	ENSOARG00000019288	ASB8	138198426	138235446	3
ENSOARG00000014589	ZFC3H1	107556327	107605339	3	ENSOARG00000019471		34958690	34959330	3	ENSOARG00000019300	PFKM	138238132	138280543	3
ENSOARG00000014616	THAP2	107606256	107618590	3	ENSOARG00000019564	TMTC1	184510212	184836655	3	ENSOARG00000019458	BABAM2	34767758	35168619	3
ENSOARG00000015027	SYT1	114906496	115404501	3	ENSOARG00000019999	KRAS	189578775	189614533	3	ENSOARG00000019471		34958690	34959330	3
ENSOARG00000015359	LRRIQ1	121148785	121363705	3	ENSOARG00000020479	RETSAT	57047396	57061814	3	ENSOARG00000019578	ANO6	140942833	141115777	3
ENSOARG00000015702	EPYC	127257338	127295253	3	ENSOARG00000020493	ELMOD3	57065579	57095703	3	ENSOARG00000019622	NELL2	141536582	141928837	3
ENSOARG00000016814	TXNRD1	172982661	173042110	3	ENSOARG00000024456		122925167	122925225	3	ENSOARG00000019909	ITPR2	187771324	188372241	3
ENSOARG00000017296	NUAK1	174550166	174621290	3	ENSOARG00000025992		84593692	84680075	3	ENSOARG00000019970	LRRK2	146551630	146764634	3
ENSOARG00000017344	BIN2	134750526	134787072	3	ENSOARG00000025993		84646174	84681092	3	ENSOARG00000019999	KRAS	189578775	189614533	3
ENSOARG00000017381	POU6F1	134849158	134869705	3	ENSOARG00000026024		131632147	131632909	3	ENSOARG00000020366	PYROXD1	193503056	193528097	3
ENSOARG00000018287		164406274	164407230	3	ENSOARG00000001140		112262485	112438114	4	ENSOARG00000020479	RETSAT	57047396	57061814	3
ENSOARG00000018639	MB	178866680	178877963	3	ENSOARG00000001288		112314512	112321148	4	ENSOARG00000020493	ELMOD3	57065579	57095703	3
ENSOARG00000019288	ASB8	138198426	138235446	3	ENSOARG00000002931	DOCK4	56152727	56624017	4	ENSOARG00000021286		96586892	96586962	3

ENSOARG00000019300	PFKM	138238132	138280543	3	ENSOARG00000006085		95239604	95287750	4	ENSOARG00000022504	5S_rRNA	118496957	118497076	3
ENSOARG00000019311	SENP1	138282207	138331370	3	ENSOARG00000006163	BBS9	63241800	63694790	4	ENSOARG00000026012		108802884	108807540	3
ENSOARG00000019578	ANO6	140942833	141115777	3	ENSOARG00000006427	PLXNA4	95788312	96204055	4	ENSOARG00000002931	DOCK4	56152727	56624017	4
ENSOARG00000019622	NELL2	141536582	141928837	3	ENSOARG00000006901	THSD7A	19499637	19777463	4	ENSOARG00000004500	NAMPT	47349250	47389103	4
ENSOARG00000019623	ERGIC2	184954799	184996084	3	ENSOARG00000007611		118351579	118355387	4	ENSOARG00000005193	KMT2C	113983981	114146610	4
ENSOARG00000019717	ADAMTS20	142961377	143166195	3	ENSOARG00000007716	BPGM	98301817	98320855	4	ENSOARG00000006427	PLXNA4	95788312	96204055	4
ENSOARG00000019994		43016611	43018465	3	ENSOARG00000008903		66590322	66929661	4	ENSOARG00000008198	AGMO	23650918	24047746	4
ENSOARG00000020390	SLCO1A2	193610779	193663029	3	ENSOARG00000010179	TRIM24	101941199	102051242	4	ENSOARG00000009641	HDAC9	27257256	27548397	4
ENSOARG00000020420		193676598	193750422	3	ENSOARG00000013517		3629296	3769877	4	ENSOARG00000009803	TWISTNB	28448451	28458663	4
ENSOARG00000020802	GRIN2B	200689548	201197462	3	ENSOARG00000014903	GRM3	33520799	33628582	4	ENSOARG00000010179	TRIM24	101941199	102051242	4
ENSOARG00000023666		115367304	115367384	3	ENSOARG00000017240	HECW1	77592533	77851748	4	ENSOARG00000013517		3629296	3769877	4
ENSOARG00000024362		200823076	200823162	3	ENSOARG00000017420	GLI3	79029288	79131016	4	ENSOARG00000014903	GRM3	33520799	33628582	4
ENSOARG00000025936		1690528	1699560	3	ENSOARG00000017524	MAGI2	42637311	43299968	4	ENSOARG00000016384	CDK14	7873876	8511553	4
ENSOARG00000026040		174577386	174723159	3	ENSOARG00000018237	TCAF1	106458935	106491976	4	ENSOARG00000016532		8367514	8371580	4
ENSOARG00000002000	BMT2	55507741	55584574	4	ENSOARG00000022797	U6	63293600	63293706	4	ENSOARG00000017240	HECW1	77592533	77851748	4
ENSOARG00000007177	SLC35B4	97934780	97969641	4	ENSOARG00000023737		33541345	33541430	4	ENSOARG00000017420	GLI3	79029288	79131016	4
ENSOARG00000014574	DMTF1	33148768	33195015	4	ENSOARG00000025211		3683214	3745148	4	ENSOARG00000023737		33541345	33541430	4
ENSOARG00000018572	CDK6	9837145	10086661	4	ENSOARG00000001932	COMMD10	35086709	35265384	5	ENSOARG00000025211		3683214	3745148	4
ENSOARG00000002870	GPRIN1	35553311	35556147	5	ENSOARG00000001949	ARHGAP26	51190660	51671936	5	ENSOARG00000025233		47308313	47330698	4
ENSOARG00000002915	SNCB	35574853	35582184	5	ENSOARG00000003045	SNAPC2	14355089	14357459	5	ENSOARG00000003045	SNAPC2	14355089	14357459	5
ENSOARG00000012188	CACTIN	17872074	17883373	5	ENSOARG00000003105	TIMM44	14359615	14371424	5	ENSOARG00000003105	TIMM44	14359615	14371424	5
ENSOARG00000012274	TBXA2R	17893368	17899126	5	ENSOARG00000012373	ADAM19	66427663	66518705	5	ENSOARG00000003640	BRD4	7579941	7664534	5
ENSOARG00000012310	GIPC3	17903084	17906791	5	ENSOARG00000013615	FSTL4	42210002	42682576	5	ENSOARG00000003767	EPHX3	7669684	7673094	5
ENSOARG00000012318	HMG20B	17910323	17915929	5	ENSOARG00000014723	CXCL14	44436779	44440881	5	ENSOARG00000008239	ZNF692	40088840	40096447	5
ENSOARG00000014004	PCDHB15	49709852	49712155	5	ENSOARG00000015800	ZGLP1	12524327	12527107	5	ENSOARG00000015794	NME5	46938902	46961998	5
ENSOARG00000016317		47131601	47133514	5	ENSOARG00000015848		12527749	12531743	5	ENSOARG00000015800	ZGLP1	12524327	12527107	5
ENSOARG00000016394	KDM3B	47153096	47208649	5	ENSOARG00000015886		12533217	12546433	5	ENSOARG00000015840	BRD8	46972869	46993265	5
ENSOARG00000016418		20379609	20597764	5	ENSOARG00000015922	ICAM3	12546272	12554248	5	ENSOARG00000015848		12527749	12531743	5
ENSOARG00000017521	HBEGF	48917000	48951593	5	ENSOARG00000016012	TYK2	12563749	12590812	5	ENSOARG00000015886		12533217	12546433	5
ENSOARG00000018844	PCDHB14	49694520	49706894	5	ENSOARG00000016463		14358880	14359035	5	ENSOARG00000015922	ICAM3	12546272	12554248	5
ENSOARG00000018880	MAN2A1	105984860	106179347	5	ENSOARG00000016808		38445338	38446264	5	ENSOARG00000016012	TYK2	12563749	12590812	5
ENSOARG00000018924	TMEM232	106642215	106911809	5	ENSOARG00000017454	SMARCA4	13107775	13181922	5	ENSOARG00000016463		14358880	14359035	5
ENSOARG00000025296		17857816	17992134	5	ENSOARG00000018880	MAN2A1	105984860	106179347	5	ENSOARG00000016502	ADGRV1	87500637	88022830	5

ENSOARG00000000537	ANK2	12362227	12719843	6	ENSOARG00000023388		13107882	13107939	5	ENSOARG00000016808		38445338	38446264	5
ENSOARG00000003887	MAPK10	100456410	100650949	6	ENSOARG00000024361	U6	51461892	51461998	5	ENSOARG00000017050	SLC27A6	22582095	22657624	5
ENSOARG00000005149	KCNIP4	40156628	40399467	6	ENSOARG00000005149	KCNIP4	40156628	40399467	6	ENSOARG00000018142	LMNB1	24625371	24679085	5
ENSOARG00000007185	STK32B	103332684	103713145	6	ENSOARG00000006066	JAKMIP1	102735767	102808907	6	ENSOARG00000023036		22620569	22620661	5
ENSOARG00000010430	PPA2	19864955	19945670	6	ENSOARG00000007030	EVC2	103130193	103289328	6	ENSOARG00000006066	JAKMIP1	102735767	102808907	6
ENSOARG00000010632	TET2	20055989	20147275	6	ENSOARG00000007954	FGFBP1	110587924	110588628	6	ENSOARG00000007030	EVC2	103130193	103289328	6
ENSOARG00000013238	SLC39A8	22554766	22635293	6	ENSOARG00000009750	NPNT	19341096	19415842	6	ENSOARG00000007954	FGFBP1	110587924	110588628	6
ENSOARG00000018214	CWH43	67594632	67647488	6	ENSOARG00000010430	PPA2	19864955	19945670	6	ENSOARG00000009491	DTHD1	55697169	55784117	6
ENSOARG00000025038	U6	19846738	19846844	6	ENSOARG00000012378	SLC4A4	86143053	86457688	6	ENSOARG00000009750	NPNT	19341096	19415842	6
ENSOARG00000003122	SPATA7	96996666	97038856	7	ENSOARG00000013955	H2AZ1	24826878	24828144	6	ENSOARG00000010172	INTS12	19612772	19634942	6
ENSOARG00000003198		97148925	97186147	7	ENSOARG00000014034	DNAJB14	24829721	24873057	6	ENSOARG00000010289	ARHGEF38	19650992	19793944	6
ENSOARG00000003285	EML5	97190353	97312451	7	ENSOARG00000014880	MTHFD2L	88783856	88905590	6	ENSOARG00000010430	PPA2	19864955	19945670	6
ENSOARG00000003343	TTC8	97364708	97423931	7	ENSOARG00000014945	EPGN	88912478	88918744	6	ENSOARG00000010632	TET2	20055989	20147275	6
ENSOARG00000016888	IQGAP2	7424541	7731473	7	ENSOARG00000023897		97042557	97042639	6	ENSOARG00000012378	SLC4A4	86143053	86457688	6
ENSOARG00000016918	F2RL2	7644832	7650996	7	ENSOARG00000003285	EML5	97190353	97312451	7	ENSOARG00000014436	C4orf54	25056613	25070887	6
ENSOARG00000020851		48514282	48670343	7	ENSOARG00000016161	EPB41L4A	1256282	1531717	7	ENSOARG00000014880	MTHFD2L	88783856	88905590	6
ENSOARG00000020856	LIPC	48680866	48853721	7	ENSOARG00000018537		15365981	15366591	7	ENSOARG00000014945	EPGN	88912478	88918744	6
ENSOARG00000021121	RTN1	68766907	68890740	7	ENSOARG00000018551		15384728	15392155	7	ENSOARG00000021631		19642785	19642861	6
ENSOARG00000021126	PPM1A	69387661	69436582	7	ENSOARG00000020717		42326861	42347419	7	ENSOARG00000023897		97042557	97042639	6
ENSOARG00000022845	U6	63342906	63343012	7	ENSOARG00000020730	OAZ2	42465465	42468293	7	ENSOARG00000003285	EML5	97190353	97312451	7
ENSOARG00000023068	5S_rRNA	48817640	48817752	7	ENSOARG00000020735	ZNF609	42475435	42642760	7	ENSOARG00000016161	EPB41L4A	1256282	1531717	7
ENSOARG00000023637		97026422	97026507	7	ENSOARG00000020996	ATP8B4	57145756	57436036	7	ENSOARG00000017673	PTGER2	11588936	11600675	7
ENSOARG00000000096	BCLAF1	61293172	61313162	8	ENSOARG00000021121	RTN1	68766907	68890740	7	ENSOARG00000020717		42326861	42347419	7
ENSOARG00000001332	VTA1	66565201	66637042	8	ENSOARG00000021191	SLC8A3	78697982	78837399	7	ENSOARG00000020893	PRTG	51635068	51773448	7
ENSOARG00000002348	STXBP5	71462635	71627320	8	ENSOARG00000026710		57124667	57275633	7	ENSOARG00000026728		94660539	94753362	7
ENSOARG00000004773	AGPAT4	83449093	83583173	8	ENSOARG00000001332	VTA1	66565201	66637042	8	ENSOARG00000004895	RPS6KA2	88034242	88188079	8
ENSOARG00000007878	NCOA7	12446609	12579375	8	ENSOARG00000006899	PHIP	5690526	5800596	8	ENSOARG00000006410	COL12A1	1763844	1881188	8
ENSOARG00000007915	HEY2	12637522	12647466	8	ENSOARG00000008929	RFX6	20778551	20830642	8	ENSOARG00000008929	RFX6	20778551	20830642	8
ENSOARG00000014082	ENPP3	56800675	56891139	8	ENSOARG00000009159		21064326	21097808	8	ENSOARG00000009159		21064326	21097808	8
ENSOARG00000027033		55303220	55340228	8	ENSOARG00000009927		26540854	26560164	8	ENSOARG00000009927		26540854	26560164	8
ENSOARG00000004022	TRAPPC9	16049980	16369216	9	ENSOARG00000009968	CDK19	26592432	26760228	8	ENSOARG00000009968	CDK19	26592432	26760228	8
ENSOARG00000015885	TOX	37775458	38082193	9	ENSOARG00000012246		44791678	45077954	8	ENSOARG00000011229		30886393	30887168	8
ENSOARG00000026520		37917875	38082193	9	ENSOARG00000012911	RNGTT	48260082	48482195	8	ENSOARG00000012911	RNGTT	48260082	48482195	8
ENSOARG00000000109	SLAIN1	53317825	53368551	10	ENSOARG00000020008		50458092	50458520	8	ENSOARG00000022716	U6	26579000	26579106	8

ENSOARG00000008448	CAB39L	19236825	19286656	10	ENSOARG00000022716	U6	26579000	26579106	8	ENSOARG00000027019		44471402	44496409	8
ENSOARG00000009964	TRPC4	24289442	24435384	10	ENSOARG00000027025		48627836	48706773	8	ENSOARG00000027025		48627836	48706773	8
ENSOARG00000010770	STARD13	28217820	28275644	10	ENSOARG00000027042		72778000	72795072	8	ENSOARG00000009412	FER1L6	28725879	28908736	9
ENSOARG00000015513	KLF12	48967445	49491521	10	ENSOARG00000009003	TRIQK	83920585	84012541	9	ENSOARG00000009876	ANXA13	29008574	29052918	9
ENSOARG00000021607		19866186	19866516	10	ENSOARG00000009876	ANXA13	29008574	29052918	9	ENSOARG00000010674	EIF3H	60424730	60523273	9
ENSOARG00000022692	7SK	53295513	53295829	10	ENSOARG00000010674	EIF3H	60424730	60523273	9	ENSOARG00000011267	CSMD3	63608850	65007715	9
ENSOARG00000024113	5S_rRNA	19303884	19304000	10	ENSOARG00000011340	MMP16	86978756	87360859	9	ENSOARG00000014889		36111034	36112627	9
ENSOARG00000001617	MYH10	27625138	27747562	11	ENSOARG00000014778	LYN	36031532	36077272	9	ENSOARG00000020180		36132430	36133062	9
ENSOARG00000003275	WFIKKN2	35444765	35453108	11	ENSOARG00000014889		36111034	36112627	9	ENSOARG00000021574	U1	36126742	36126906	9
ENSOARG00000004112	TRAF4	19788399	19794104	11	ENSOARG00000020180		36132430	36133062	9	ENSOARG00000023724	SNORD54	36111884	36111950	9
ENSOARG00000004162	FAM222B	19799477	19808342	11	ENSOARG00000021574	U1	36126742	36126906	9	ENSOARG00000026514		27103578	27106919	9
ENSOARG00000004170		23663655	23664599	11	ENSOARG00000023724	SNORD54	36111884	36111950	9	ENSOARG00000000618	DCT	69452806	69492878	10
ENSOARG00000004204		23761925	23762614	11	ENSOARG00000026514		27103578	27106919	9	ENSOARG00000000718	TGDS	69480374	69590150	10
ENSOARG00000004221		23776332	23777273	11	ENSOARG00000000372	RBM26	54918357	54994260	10	ENSOARG00000006244	TDRD3	1835291	2065511	10
ENSOARG00000005519	PIPOX	20029543	20041627	11	ENSOARG00000000618	DCT	69452806	69492878	10	ENSOARG00000006641		7450054	7547626	10
ENSOARG00000011855		45762020	45802167	11	ENSOARG00000000718	TGDS	69480374	69590150	10	ENSOARG00000008395	DCUN1D2	85997258	86013384	10
ENSOARG00000012131	IKZF3	39697587	39784473	11	ENSOARG00000006515	COL4A2	84344246	84506524	10	ENSOARG00000008433	TMCO3	86021273	86037791	10
ENSOARG00000014197		47626004	47629928	11	ENSOARG00000006641		7450054	7547626	10	ENSOARG00000008441	TFDP1	86049251	86064887	10
ENSOARG00000014212		47641431	47645553	11	ENSOARG00000008395	DCUN1D2	85997258	86013384	10	ENSOARG00000002872	MBTD1	35042066	35112724	11
ENSOARG00000014652	TNS4	40276427	40294223	11	ENSOARG00000008433	TMCO3	86021273	86037791	10	ENSOARG00000004029		21248088	21248303	11
ENSOARG00000015860		18748955	18836988	11	ENSOARG00000008441	TFDP1	86049251	86064887	10	ENSOARG00000007435	PRPSAP1	54397258	54419080	11
ENSOARG00000016186		18969861	18988041	11	ENSOARG00000001819	KIAA0100	19685646	19712677	11	ENSOARG00000007848		7641415	8038744	11
ENSOARG00000016763		23686551	23829312	11	ENSOARG00000002429	SDF2	19713756	19724240	11	ENSOARG00000007910		7997170	7998371	11
ENSOARG00000022595	U1	45765834	45765989	11	ENSOARG00000002727	SUPT6H	19732556	19754769	11	ENSOARG00000008340	TBX21	38271542	38282106	11
ENSOARG00000023268	5S_rRNA	19809497	19809594	11	ENSOARG00000005673	TK1	52963145	52975382	11	ENSOARG00000008406	TBKBP1	38304081	38318431	11
ENSOARG00000023893		27736355	27736449	11	ENSOARG00000005753		52977906	52978917	11	ENSOARG00000009006	NSRP1	20719581	20905075	11
ENSOARG00000024896	Y_RNA	23775149	23775252	11	ENSOARG00000005846	TMC8	53004485	53012803	11	ENSOARG00000009283		20761413	20766654	11
ENSOARG00000026354		18613777	18618841	11	ENSOARG00000006979	RHBDF2	54270255	54278688	11	ENSOARG00000009452	EFCAB5	20769784	20856633	11
ENSOARG00000026373		40159758	40312440	11	ENSOARG00000007435	PRPSAP1	54397258	54419080	11	ENSOARG00000010985	TRARG1	21229125	21240170	11
ENSOARG00000013714	CD34	73589869	73605431	12	ENSOARG00000007848		7641415	8038744	11	ENSOARG00000017855	CHD3	27132435	27152030	11
ENSOARG00000013891		73700588	73737242	12	ENSOARG00000007910		7997170	7998371	11	ENSOARG00000022732	SCARNA21	27146908	27147047	11
ENSOARG00000025427		9383068	9394916	12	ENSOARG00000008340	TBX21	38271542	38282106	11	ENSOARG00000003648	CCNL2	49206920	49216787	12
ENSOARG00000025469		75594465	75601647	12	ENSOARG00000008406	TBKBP1	38304081	38318431	11	ENSOARG00000003838	AURKAIP1	49217180	49222771	12

ENSOARG00000002139	TTLL9	60717466	60772689	13	ENSOARG00000009006	NSRP1	20719581	20905075	11	ENSOARG00000003922	MXRA8	49244103	49247665	12
ENSOARG00000002249	ADAM33	50839347	50852297	13	ENSOARG00000009283		20761413	20766654	11	ENSOARG00000004655	KIF26B	29798435	30115631	12
ENSOARG00000002391	MSRB2	23184943	23206285	13	ENSOARG00000009452	EFCAB5	20769784	20856633	11	ENSOARG00000005151	HMCN1	64798855	65337057	12
ENSOARG00000002416	PDRG1	60775487	60781584	13	ENSOARG00000026386		52845989	52980516	11	ENSOARG00000005190	ACTRT2	48089727	48090860	12
ENSOARG00000002457		50858086	50858543	13	ENSOARG00000026389		54096210	54271589	11	ENSOARG00000012654	RAB3GAP2	21760318	21865369	12
ENSOARG00000002481	XKR7	60797386	60822789	13	ENSOARG00000026390		54225864	54263405	11	ENSOARG00000013652	VAMP4	37302039	37323489	12
ENSOARG00000002564		60835911	60855548	13	ENSOARG00000002070	RGL1	62892208	63060293	12	ENSOARG00000014645	TNFSF18	38815643	38823860	12
ENSOARG00000002627	ATRN	50871319	51003428	13	ENSOARG00000002600	C1orf21	63602428	63754041	12	ENSOARG00000015833	TP73	47455866	47502821	12
ENSOARG00000002820	HCK	60874932	60921921	13	ENSOARG00000004302	C1orf74	71659955	71660764	12	ENSOARG00000022872	5S_rRNA	37974675	37974795	12
ENSOARG00000003062	TM9SF4	60954424	60985439	13	ENSOARG00000005190	ACTRT2	48089727	48090860	12	ENSOARG00000023516	SNORA36B	21811325	21811456	12
ENSOARG00000015308	CNBD2	64673075	64723487	13	ENSOARG00000009688		42897047	43151892	12	ENSOARG00000025437		23567301	23687517	12
ENSOARG00000018635	DZANK1	37599912	37653541	13	ENSOARG00000010714	USH2A	16999001	17981947	12	ENSOARG00000025438		23570015	23657488	12
ENSOARG00000024427		9087698	9087778	13	ENSOARG00000011000	DNAJC11	44817017	44884541	12	ENSOARG00000025467		73101430	73103824	12
ENSOARG00000026248		64684046	64849522	13	ENSOARG00000012654	RAB3GAP2	21760318	21865369	12	ENSOARG00000001355		68972583	68976410	13
ENSOARG00000008034	MAF	5921193	5923225	14	ENSOARG00000012720	IRF6	71642946	71654633	12	ENSOARG00000005583		73399575	73401407	13
ENSOARG00000008495		50531926	50544883	14	ENSOARG00000012918	TRAF3IP3	71661157	71680824	12	ENSOARG00000018399	TTI1	66454593	66484327	13
ENSOARG00000008501		50559956	50563749	14	ENSOARG00000013652	VAMP4	37302039	37323489	12	ENSOARG00000018960	RALGAPB	66983273	67058489	13
ENSOARG00000011355	BICRA	53602524	53676728	14	ENSOARG00000018630	RER1	48497785	48504352	12	ENSOARG00000019100	ADIG	67064926	67070255	13
ENSOARG00000011391	EHD2	53684814	53699975	14	ENSOARG00000018748	MORN1	48507947	48556384	12	ENSOARG00000005978	ACP7	47997870	48011505	14
ENSOARG00000023231		50538118	50538246	14	ENSOARG00000021674	U6	62994383	62994485	12	ENSOARG00000011673		54045348	54057456	14
ENSOARG00000026909		23102469	23275357	14	ENSOARG00000022000		73782644	73782731	12	ENSOARG00000011682		54063550	54086186	14
ENSOARG00000010872	OR9I1	78493970	78662782	15	ENSOARG00000023516	SNORA36B	21811325	21811456	12	ENSOARG00000017887	RPGRIP1L	21426273	21521065	14
ENSOARG00000011446		79226138	79261515	15	ENSOARG00000024409	oar-mir-29b-1	73782083	73782166	12	ENSOARG00000017939	FTO	21524991	21953995	14
ENSOARG00000012905	OR9Q2	78588099	78589037	15	ENSOARG00000024729		63718660	63718749	12	ENSOARG00000005911	INSC	36936849	37000817	15
ENSOARG00000013164		80389134	80412010	15	ENSOARG00000025437		23567301	23687517	12	ENSOARG00000008069	TEAD1	39369981	39631064	15
ENSOARG00000013240	ACER3	54380118	54544096	15	ENSOARG00000025438		23570015	23657488	12	ENSOARG00000008298	PARVA	39785022	39847574	15
ENSOARG00000015424	C1QTNF5	29389458	29390834	15	ENSOARG00000006091		73855033	73868280	13	ENSOARG00000010050	GALNT18	40834788	41017549	15
ENSOARG00000015673	MFRP	29390596	29396511	15	ENSOARG00000011254	NDUFAF5	6872875	6909267	13	ENSOARG00000011592	GLYATL2	79280017	79288198	15
ENSOARG00000015942	USP2	29405834	29421267	15	ENSOARG00000011461	SEL1L2	6917382	7009037	13	ENSOARG00000013785	C11orf53	20958217	20998001	15
ENSOARG00000017054	RNF26	29385024	29386343	15	ENSOARG00000017498	PCK1	57900057	57905307	13	ENSOARG00000013805	COLCA2	21009691	21017209	15
ENSOARG00000019097	IFTAP	65231458	65301531	15	ENSOARG00000017959	RAE1	58073348	58092870	13	ENSOARG00000014095	ANO3	55194747	55486464	15
ENSOARG00000026816		22387770	22390565	15	ENSOARG00000018002	SPO11	58100842	58115189	13	ENSOARG00000015999	IMMP1L	60430839	60496840	15

ENSOARG00000005717		44809323	44809910	16	ENSOARG00000018399	TTI1	66454593	66484327	13	ENSOARG00000018423	UBASH3B	32853387	33003019	15
ENSOARG00000008401	PARP8	28331163	28521803	16	ENSOARG00000005978	ACP7	47997870	48011505	14	ENSOARG00000005937	MAST4	12421835	13041016	16
ENSOARG00000010729	SLC1A3	37483723	37563198	16	ENSOARG00000007080		2787215	3071357	14	ENSOARG00000010187	WDR70	36614984	36892613	16
ENSOARG00000012630	CDH6	42084521	42144160	16	ENSOARG00000011673		54045348	54057456	14	ENSOARG00000014702	ADCY2	65280195	65721184	16
ENSOARG00000012793		48883132	48884676	16	ENSOARG00000011682		54063550	54086186	14	ENSOARG00000003364	DDX55	51651684	51668573	17
ENSOARG00000001443	FSTL5	35976685	36901504	17	ENSOARG00000017887	RPGRIP1L	21426273	21521065	14	ENSOARG00000006474	GLRB	41763480	41856985	17
ENSOARG00000011798	INPP4B	15088639	15543935	17	ENSOARG00000017939	FTO	21524991	21953995	14	ENSOARG00000017566		71881383	72067452	17
ENSOARG00000013508		70559502	70862523	17	ENSOARG00000005911	INSC	36936849	37000817	15	ENSOARG00000025722		71986609	71987687	17
ENSOARG00000014207	ADORA2A	70859260	70868363	17	ENSOARG00000007202	SPON1	38044689	38354554	15	ENSOARG00000004861	STXBP6	33919740	34146066	18
ENSOARG00000015506	STX2	45920664	45945399	17	ENSOARG00000010050	GALNT18	40834788	41017549	15	ENSOARG00000000381		47798272	47835571	19
ENSOARG00000017120	TMEM132B	49885384	50186678	17	ENSOARG00000011592	GLYATL2	79280017	79288198	15	ENSOARG00000005680	WDR82	48598004	48612296	19
ENSOARG00000007087		44166626	44194878	18	ENSOARG00000014095	ANO3	55194747	55486464	15	ENSOARG00000005786		48617258	48622991	19
ENSOARG00000009711	CERS3	5141549	5358514	18	ENSOARG00000014873		21546689	21652451	15	ENSOARG00000005975	TWF2	48631479	48639397	19
ENSOARG00000009906	TTC23	6609859	6721193	18	ENSOARG00000015999	IMMP1L	60430839	60496840	15	ENSOARG00000007668	GRM7	18544027	19149524	19
ENSOARG00000009990		6726371	6750105	18	ENSOARG00000017917	GRIK4	30791427	31136076	15	ENSOARG00000009293	CNTN3	27107120	27401680	19
ENSOARG00000000458	SFMBT1	47861509	47970530	19	ENSOARG00000004394	FAM169A	6714430	6779035	16	ENSOARG00000009783		31470263	31470667	19
ENSOARG00000007465	PLXNA1	60171949	60201198	19	ENSOARG00000006371	ADAMTS6	14045920	14324177	16	ENSOARG00000010162	SACM1L	53478986	53532306	19
ENSOARG00000008494		49530241	49701797	19	ENSOARG00000014702	ADCY2	65280195	65721184	16	ENSOARG00000012724	FLNB	43152605	43292791	19
ENSOARG00000009813	SLC6A20	53424967	53471152	19	ENSOARG00000003095		67923976	67931604	17	ENSOARG00000014390	ASB14	43836052	43850342	19
ENSOARG00000010162	SACM1L	53478986	53532306	19	ENSOARG00000003364	DDX55	51651684	51668573	17	ENSOARG00000014531	APPL1	43854675	43876438	19
ENSOARG00000003613	UBR2	16358563	16459598	20	ENSOARG00000004146	FBXW7	4965419	5185723	17	ENSOARG00000024538	oar-let-7g	48603621	48603703	19
ENSOARG00000006254	DNPH1	16901274	16903616	20	ENSOARG00000019041	GRK3	65098471	65205105	17	ENSOARG00000003926	CARMIL1	31188499	31494237	20
ENSOARG00000006417	TTBK1	16926897	16964261	20	ENSOARG00000004861	STXBP6	33919740	34146066	18	ENSOARG00000005388		16742146	16747111	20
ENSOARG00000006418	HMGCLL1	4979745	5104686	20	ENSOARG00000026453		48601602	48649268	18	ENSOARG00000005475	KLC4	16748932	16763518	20
ENSOARG00000010300	SUPT3H	18310799	18706618	20	ENSOARG00000006974	GRM2	49084945	49093511	19	ENSOARG00000005603	KHDRBS2	204311	1038981	20
ENSOARG00000010416		8003296	8006987	20	ENSOARG00000007668	GRM7	18544027	19149524	19	ENSOARG00000005654	PRIM2	2673361	2977511	20
ENSOARG00000010446	LEMD2	8008193	8024668	20	ENSOARG00000009293	CNTN3	27107120	27401680	19	ENSOARG00000008692		10643936	10644727	20
ENSOARG00000012272	FKBP5	9692040	9766541	20	ENSOARG00000012724	FLNB	43152605	43292791	19	ENSOARG00000010300	SUPT3H	18310799	18706618	20
ENSOARG00000016687	KIF6	13239546	13661152	20	ENSOARG00000014390	ASB14	43836052	43850342	19	ENSOARG00000022664	5S_rRNA	18421702	18421834	20
ENSOARG00000018718		48062157	48322346	20	ENSOARG00000014531	APPL1	43854675	43876438	19	ENSOARG00000026888		46594647	46727238	20
ENSOARG00000022664	5S_rRNA	18421702	18421834	20	ENSOARG00000016371	ARPP21	9569024	9683317	19	ENSOARG00000005183	DLG2	10779194	11743008	21
ENSOARG00000002815	PACS1	43458201	43604405	21	ENSOARG00000003926	CARMIL1	31188499	31494237	20	ENSOARG00000007919		23117728	23624748	21
ENSOARG00000003296	KLC2	43616119	43624384	21	ENSOARG00000005654	PRIM2	2673361	2977511	20	ENSOARG00000002088	GRK5	38323057	38552331	22
ENSOARG00000003523	RAB1B	43626127	43634738	21	ENSOARG00000006418	HMGCLL1	4979745	5104686	20	ENSOARG00000008134	SORCS3	24298347	24944741	22

ENSOARG00000003549	CNIH2	43635150	43640422	21
ENSOARG00000003628	YIF1A	43641414	43645241	21
ENSOARG00000003837	TMEM151A	43647082	43651665	21
ENSOARG00000013080	DOCK1	45364051	45875506	22
ENSOARG00000013216	PCDH15	4372641	5337209	22
ENSOARG00000015307	PANK1	10990194	11046738	22
ENSOARG00000004215	NFATC1	748323	799990	23
ENSOARG00000005109	WDR7	56218845	56550504	23
ENSOARG00000009572	METTL4	37178727	37203415	23
ENSOARG00000009604	NDC80	37208585	37244969	23
ENSOARG00000026144		657340	807916	23
ENSOARG00000018068		36450665	36482827	24
ENSOARG00000018283	CYP3A24	36495420	36645659	24
ENSOARG00000021762	SNORA70	36478706	36478805	24
ENSOARG00000001975		33053238	33156974	26
ENSOARG00000004073	PSD3	37045901	37438136	26
ENSOARG00000006518	GPM6A	6098874	6137326	26

ENSOARG00000008945		28551893	28552753	20
ENSOARG00000008954		28557828	28558730	20
ENSOARG00000009595	CDKAL1	35755504	36365789	20
ENSOARG00000013288	PKHD1	23851546	24291885	20
ENSOARG00000013835	PHACTR1	42250178	42485827	20
ENSOARG00000023426		42320808	42320894	20
ENSOARG00000026888		46594647	46727238	20
ENSOARG00000007919		23117728	23624748	21
ENSOARG00000006805	CFAP43	23775242	23873559	22
ENSOARG00000015874	DPYSL4	49803446	49814634	22
ENSOARG00000015957	SEMA4G	20958608	20967864	22
ENSOARG00000016009	STK32C	49813070	49828736	22
ENSOARG00000016032	MRPL43	20963683	20971186	22
ENSOARG00000016072	TWNK	20971840	20976636	22
ENSOARG00000016108	LZTS2	20984288	20988117	22
ENSOARG00000016146	PDZD7	20989622	21007888	22
ENSOARG00000005288	KIAA1328	20111910	20525120	23
ENSOARG00000005668	SEC11C	58373833	58382590	23
ENSOARG00000005828	NOL4	23148484	23611340	23
ENSOARG00000005981	PIGN	60716730	60819102	23
ENSOARG00000009530	YES1	35920734	35941754	23
ENSOARG00000004594		40504639	40511497	24
ENSOARG00000004697	IQCE	40511246	40551800	24
ENSOARG00000010673		28285509	28426315	24
ENSOARG00000011276		32699946	32762145	24
ENSOARG00000015554	POLR3E	19838976	20025969	24
ENSOARG00000015653	EEF2K	19931674	19979917	24
ENSOARG00000016868	NYAP1	35953645	35960797	24
ENSOARG00000016889	TSC22D4	35965515	35974905	24
ENSOARG00000016895	C7orf61	35976425	35979161	24
ENSOARG00000025880		17739739	17741901	24
ENSOARG00000025902		40413481	40551726	24
ENSOARG00000004227	CCDC6	15389097	15444034	25

ENSOARG00000005288	KIAA1328	20111910	20525120	23
ENSOARG00000005828	NOL4	23148484	23611340	23
ENSOARG00000023248		52091263	52091341	23
ENSOARG00000004594		40504639	40511497	24
ENSOARG00000004697	IQCE	40511246	40551800	24
ENSOARG00000010673		28285509	28426315	24
ENSOARG00000011276		32699946	32762145	24
ENSOARG00000011868	EIF4H	33115721	33286879	24
ENSOARG00000014703	MYL10	35085594	35097624	24
ENSOARG00000014711	COL26A1	35124398	35319320	24
ENSOARG00000015554	POLR3E	19838976	20025969	24
ENSOARG00000015653	EEF2K	19931674	19979917	24
ENSOARG00000016868	NYAP1	35953645	35960797	24
ENSOARG00000016889	TSC22D4	35965515	35974905	24
ENSOARG00000016895	C7orf61	35976425	35979161	24
ENSOARG00000025891		33169760	33290472	24
ENSOARG00000025893		35123737	35257272	24
ENSOARG00000025902		40413481	40551726	24
ENSOARG00000000131	TEX15	25629926	25693596	26
ENSOARG00000003976	SH2D4A	36739622	36795389	26

ENSOARG00000006382	UNC5B	27264279	27284918	25
ENSOARG00000006506	SLC29A3	27298129	27343049	25
ENSOARG00000021769		23978131	23978273	25
ENSOARG00000026405		18960060	19006408	25

Supplementary Table S5. Estimates of Runs of Homozygosity (ROH) for each population in five different length categories

	AFS	ARB	BGA	BGE	BLO	CHA	GAR	GDK	IBL	IDC	LOR	MOG	QEZ	ZEL
0-2 Mb	1136	730	2079	2303	817	1285	2528	753	2341	1606	1656	1052	1102	1840
2-4 Mb	280	187	1070	1109	208	431	1437	179	1308	440	502	214	277	419
4-8 Mb	113	65	451	354	32	150	438	52	991	38	134	46	51	56
8-16 Mb	107	16	196	158	12	116	131	26	577	15	60	34	46	26
<16 Mb	66	2	85	181	3	95	71	8	190	10	23	20	31	22
Sum	1702	1000	3881	4105	1072	2077	4605	1018	5407	2109	2375	1366	1507	2363

Supplementary **Figure S1**. Principal components analysis (PC 1 and PC 2) of among 14 sheep breeds based on autosomes. For breed abbreviations, see Table 1.

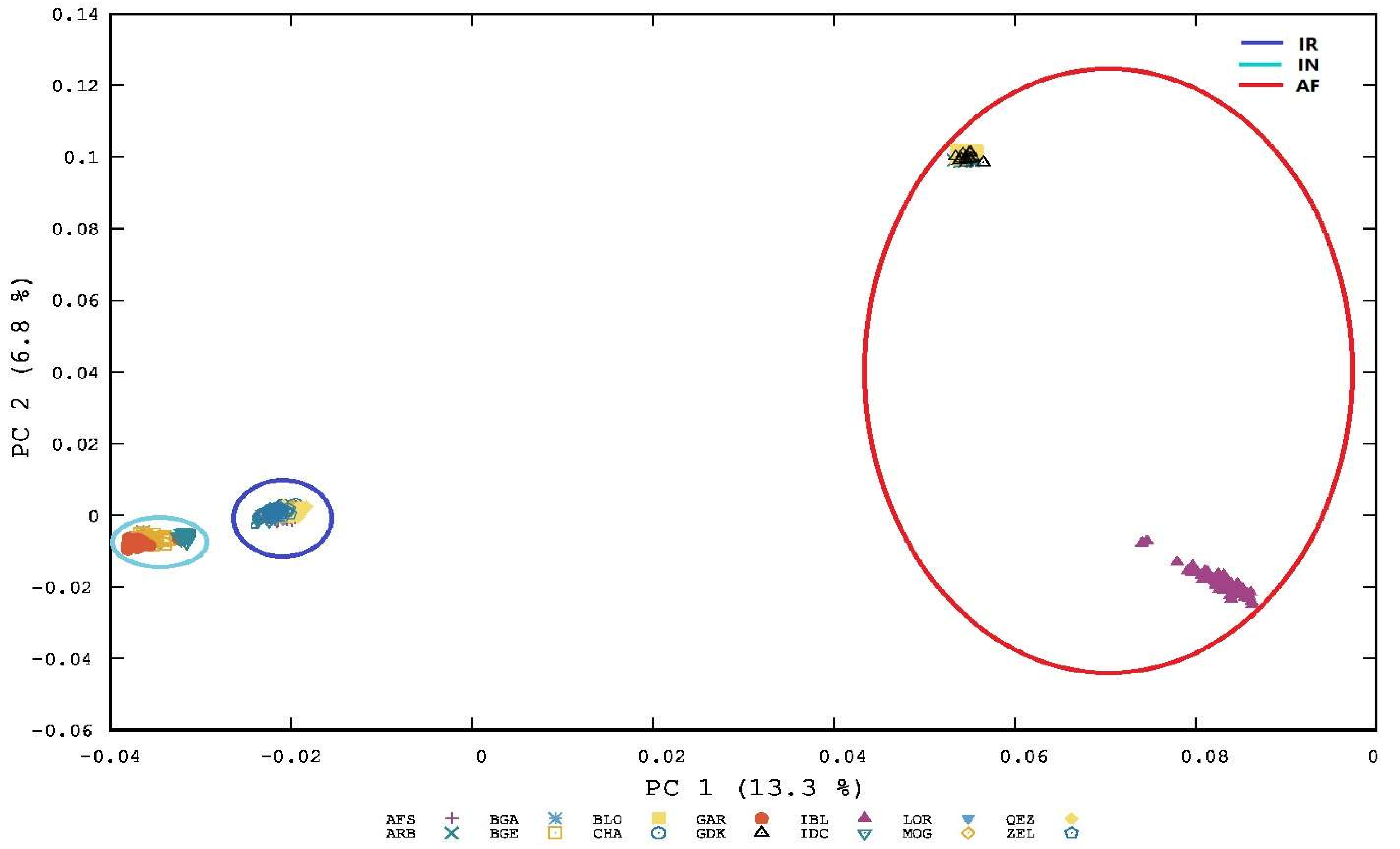

Supplementary **Figure S2**. Principal components analysis (PC 1 and PC 2) of among 10 IR and IN sheep breeds based on autosomes. For breed abbreviations, see Table 1.

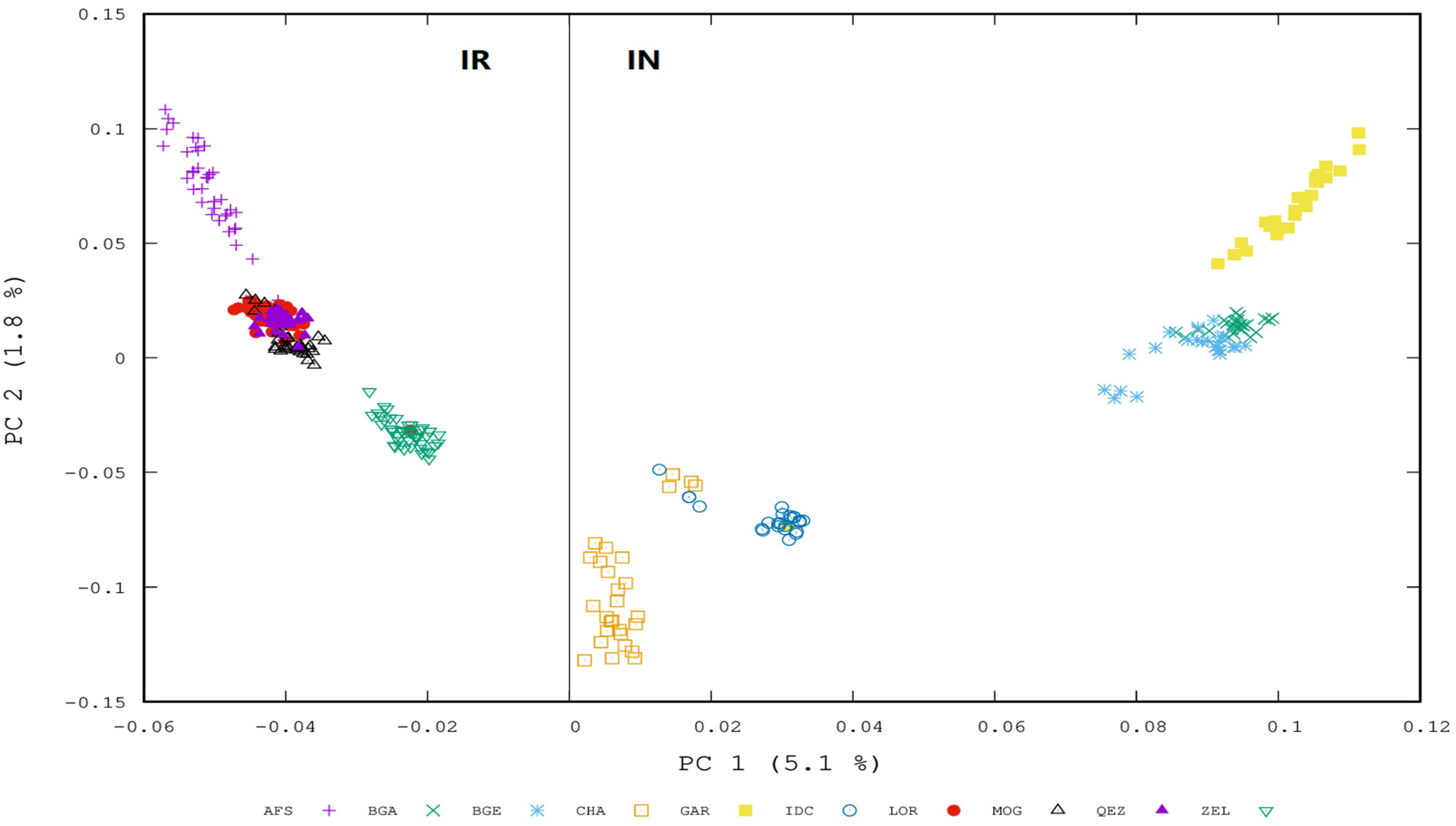

Supplementary **Figure S3**. Linkage disequilibrium decay of the IR (blue), IN (green), and AF(red) sheep breeds.

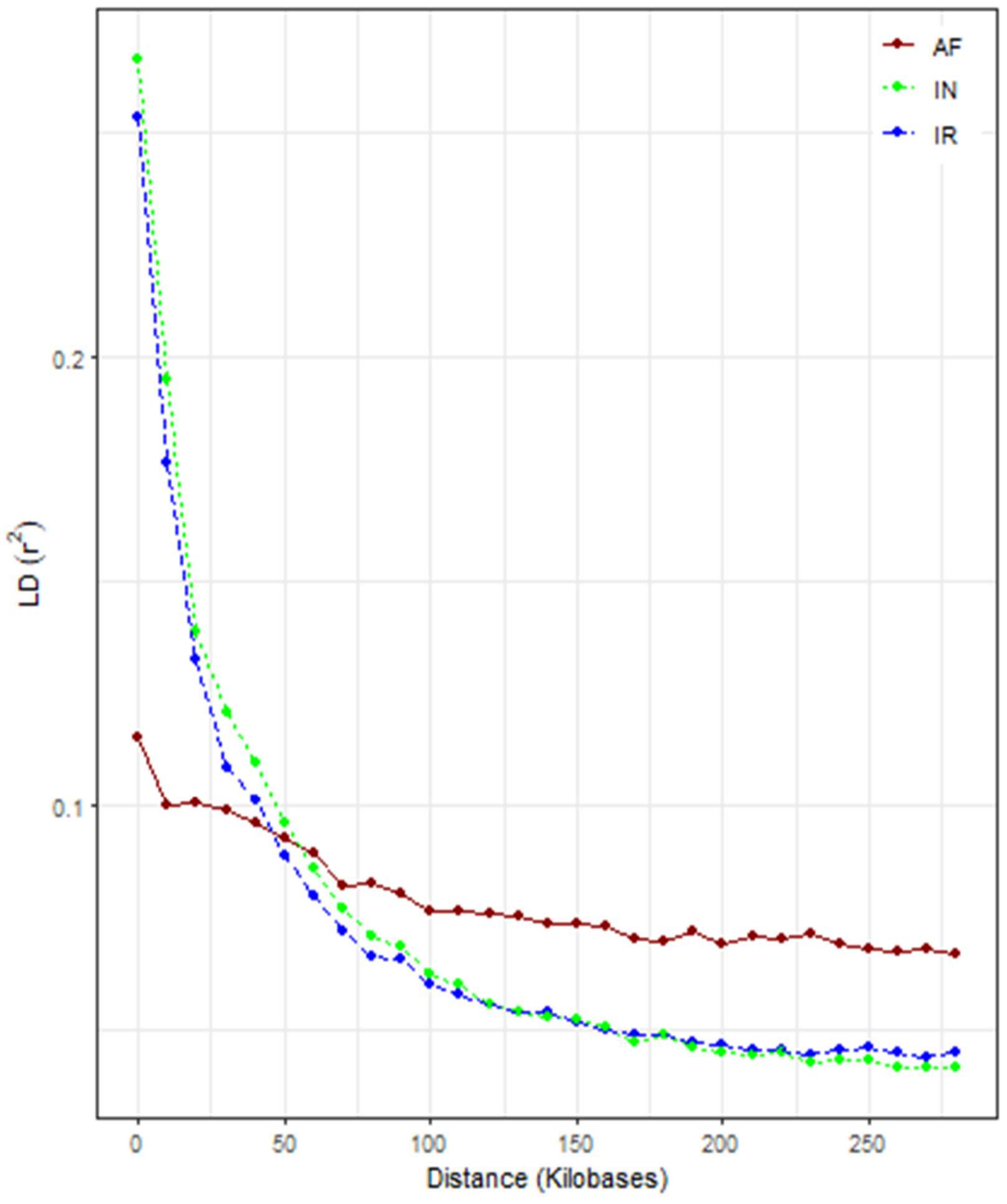

Supplementary Figure S4. The history of inbreeding coefficient in all populations base on the length distribution of ROH segments. For breed abbreviations see Table 1.

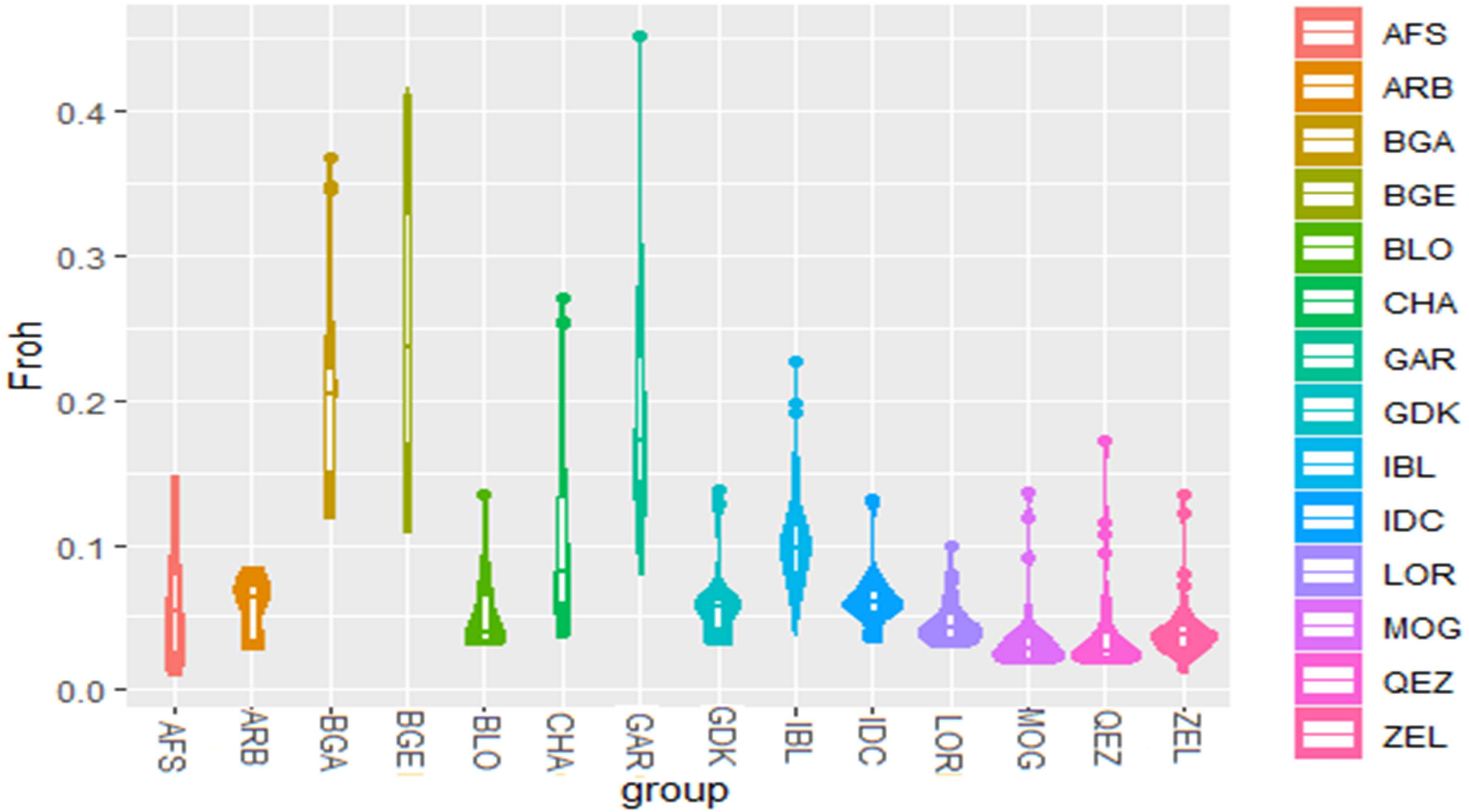

Supplement: Supplementary file 1 — Supplementary Information [file 41598_2021_82625_MOESM1_ESM.pdf]
